# Supplementary material for: Progression from external pilot to definitive randomised controlled trial: a methodological review of progression criteria reporting
Source: BMJ Open. 2021 Jun 28;11(6):e048178. doi: 10.1136/bmjopen-2020-048178 (PMC8240572; doi:10.1136/bmjopen-2020-048178)
Supplement: Supplementary data [file bmjopen-2020-048178supp001.pdf]

SUPPLEMENTARY FILE 1: SEARCH STRATEGY

[PubMed search 06 Jan 2020]

No	Search terms	Results
1	**Pilot[Title]**	68704
2	**Feasibility[Title]**	31478
3	1 OR 2	98358
4	**Trial[Title/Abstract]**	576760
5	**Study[Title/Abstract]**	7478840
6	**Protocol[Title/Abstract]**	300466
7	4 OR 5 OR 6	7872797
8	**(((("Pilot and feasibility studies"[Journal])) OR "Trials"[Journal]) OR "BMJ open"[Journal]) OR "PloS one"[Journal]**	245189
9	3 AND 7 AND 8	2529
10	**9 AND 2018.01.01"[Date - Publication] : "2019.12.31"[Date - Publication]**	1030
