# Supplementary material for: Progression from external pilot to definitive randomised controlled trial: a methodological review of progression criteria reporting
Source: BMJ Open. 2021 Jun 28;11(6):e048178. doi: 10.1136/bmjopen-2020-048178 (PMC8240572; doi:10.1136/bmjopen-2020-048178)
Supplement: Supplementary data [file bmjopen-2020-048178supp002.pdf]

BMJ Publishing Group Limited (BMJ) disclaims all liability and responsibility arising from any reliance placed on this supplemental material which has been supplied by the author(s)


**SUPPLEMENTARY FILE 2: LIST OF INCLUDED STUDIES**

| **First author** | **Year** | **Journal** | **Publication title** |
|---|---|---|---|
| Ali | 2018 | *BMJ Open* | Individual cognitive stimulation therapy for people with intellectual disability and dementia: Protocol of a feasibility randomised controlled trial |
| Battle | 2019 | *BMJ Open* | A multicentre randomised feasibility STUdy evaluating the impact of a prognostic model for Management of BLunt chest wall trauma patients: STUMBL Trial |
| Collings | 2019 | *BMJ Open* | INSoles to Ease Pressure (INSTEP) Study: A multicentre, randomised controlled feasibility study to compare the effectiveness of a novel instant optimised insole with a standard insole for people with diabetic neuropathy: A study protocol |
| Dean | 2018 | *BMJ Open* | Community-based rehabilitation training after stroke: Results of a pilot randomised controlled trial (ReTrain) investigating acceptability and feasibility |
| Dinneen | 2019 | *BMJ Open* | NeuroSAFE robot-assisted laparoscopic prostatectomy versus standard robot-assisted laparoscopic prostatectomy for men with localised prostate cancer (NeuroSAFE PROOF): Protocol for a randomised controlled feasibility study |
| Edwards | 2019 | *BMJ Open* | Novel ACT-based eHealth psychoeducational intervention for students with mental distress: A study protocol for a mixed-methodology pilot trial |
| Froghi | 2019 | *BMJ Open* | Ward-based Goal-Directed Fluid Therapy (GDFT) in Acute Pancreatitis (GAP) trial: Study protocol for a feasibility randomised controlled trial |
| Furlano | 2019 | *BMJ Open* | Feasibility of a 6-month pilot randomised controlled trial of resistance training on cognition and brain health in Canadian older adults at-risk for diabetes: Study protocol |
| Geraghty | 2018 | *BMJ Open* | Using an internet intervention to support self-management of low back pain in primary care: Findings from a randomised controlled feasibility trial (SupportBack) |
| Griffin | 2019 | *BMJ Open* | Healthy Dads, Healthy Kids UK, a weight management programme for fathers: Feasibility RCT |
| Guagliano | 2019 | *BMJ Open* | Whole family-based physical activity promotion intervention: The Families Reporting Every Step to Health pilot randomised controlled trial protocol |
| Harper | 2018 | *BMJ Open* | Treatment of fatigue with physical activity and behavioural change support in vasculitis: Study protocol for an open-label randomised controlled feasibility study |
| Hawley-Hague | 2019 | *BMJ Open* | Can smartphone technology be used to support an effective home exercise intervention to prevent falls amongst community dwelling older adults?: The TOGETHER feasibility RCT study protocol |

| | | | |
|---|---|---|---|
| Hughes | 2018 | *BMJ Open* | Prediabetes in pregnancy, can early intervention improve outcomes? A feasibility study for a parallel randomised clinical trial |
| Jolly | 2018 | *BMJ Open* | Protocol for a feasibility trial for improving breast feeding initiation and continuation: Assets-based infant feeding help before and after birth (ABA) |
| Jones | 2019 | *BMJ Open* | Walk, Talk and Listen: A pilot randomised controlled trial targeting functional fitness and loneliness in older adults with hearing loss |
| Keene | 2019 | *BMJ Open* | Progressive functional exercise versus best practice advice for adults aged 50 years or over after ankle fracture: Protocol for a pilot randomised controlled trial in the UK - The Ankle Fracture Treatment: Enhancing Rehabilitation (AFTER) study |
| Lewis | 2019 | *BMJ Open* | Cuff Leak Test and Airway Obstruction in Mechanically Ventilated ICU Patients (COMIC): A pilot randomised controlled trial protocol |
| Limond | 2019 | *BMJ Open* | Clinical and cost-effectiveness of teen online problem-solving for adolescents who have survived an acquired brain injury in the UK: Protocol for a randomised, controlled feasibility study (TOPS-UK) |
| Lockstone | 2019 | *BMJ Open* | Non-Invasive Positive airway Pressure thErapy to Reduce Postoperative Lung complications following Upper abdominal Surgery (NIPPER PLUS): protocol for a single-centre, pilot, randomised controlled trial |
| Lorenzini | 2019 | *BMJ Open* | Measuring changes in device use of a head-mounted low vision aid after personalised telerehabilitation: Protocol for a feasibility study |
| McIntyre | 2018 | *BMJ Open* | FLUID trial: A protocol for a hospital-wide open-label cluster crossover pragmatic comparative effectiveness randomised pilot trial |
| Mcpherson | 2019 | *BMJ Open* | Children and teens in charge of their health (catch): A protocol for a feasibility randomised controlled trial of solution-focused coaching to foster healthy lifestyles in childhood disability |
| Morris | 2019 | *BMJ Open* | Dietary Approaches to the Management of type 2 Diabetes (DIAMOND): Protocol for a randomised feasibility trial |
| Munce | 2019 | *BMJ Open* | Ontario Brain Injury Association Peer Support Program: A mixed methods protocol for a pilot randomised controlled trial |
| Neves | 2019 | *BMJ Open* | Protocol for a feasibility study of a cohort embedded randomised controlled trial comparing NEphron Sparing Treatment (NEST) for small renal masses |
| O'Connor | 2019 | *BMJ Open* | SAFETEL randomised controlled feasibility trial of a safety planning intervention with follow-up telephone contact to reduce suicidal behaviour: Study protocol |
| Orkin | 2019 | *BMJ Open* | Protocol for a mixed-methods feasibility study for the surviving opioid overdose with naloxone education and resuscitation (SOONER) randomised control trial |

| | | | |
|---|---|---|---|
| Pai | 2019 | *BMJ Open* | Protocol for a double-blind, randomised, placebo-controlled pilot study for assessing the feasibility and efficacy of faecal microbiota transplant in a paediatric Crohn's disease population: PediCRaFT Trial |
| Papathanassoglou | 2019 | *BMJ Open* | Relaxation for Critically ill Patient Outcomes and S tress-coping Enhancement (REPOSE): A protocol for a pilot randomised trial of an integrative intervention to improve critically ill patients' delirium and related outcomes |
| Pennington | 2019 | *BMJ Open* | Internet delivery of intensive speech and language therapy for children with cerebral palsy: A pilot randomised controlled trial |
| Pouw | 2018 | *BMJ Open* | Hospital at Home care for older patients with cognitive impairment: A protocol for a randomised controlled feasibility trial |
| Quraishi | 2019 | *BMJ Open* | STOP-Colitis pilot trial protocol: A prospective, open-label, randomised pilot study to assess two possible routes of faecal microbiota transplant delivery in patients with ulcerative colitis |
| Reddington | 2018 | *BMJ Open* | Does early intervention improve outcomes in the physiotherapy management of lumbar radicular syndrome? Results of the POLAR pilot randomised controlled trial |
| Ribeiro | 2019 | *BMJ Open* | Effectiveness of a tailored rehabilitation versus standard strengthening programme for patients with shoulder pain: A protocol for a feasibility randomised controlled trial (the Otago MASTER trial) |
| Schults | 2018 | *BMJ Open* | Normal saline instillation versus no normal saline instillation and lung Recruitment versus no lung recruitment with paediatric Endotracheal Suction: The NARES trial. A study protocol for a pilot, factorial randomised controlled trial |
| Sharma | 2018 | *BMJ Open* | Pain education for patients with non-specific low back pain in Nepal: Protocol of a feasibility randomised clinical trial (PEN-LBP Trial) |
| Sharma | 2019 | *BMJ Open* | Results of a feasibility randomised clinical trial on pain education for low back pain in Nepal: The Pain Education in Nepal-Low Back Pain (PEN-LBP) feasibility trial |
| Steare | 2019 | *BMJ Open* | App to support Recovery in Early Intervention Services (ARIES) study: Protocol of a feasibility randomised controlled trial of a self-management Smartphone application for psychosis |
| Sugg | 2018 | *BMJ Open* | Morita Therapy for depression (Morita Trial): A pilot randomised controlled trial |
| Thyer | 2018 | *BMJ Open* | Randomised controlled feasibility trial of the Active Communication Education programme plus hearing aid provision versus hearing aid provision alone (ACE to HEAR): A study protocol |
| Wall | 2018 | *BMJ Open* | Safety and feasibility evaluation of tourniquets for total knee replacement (SAFE-TKR): Study protocol |
| Wiangkham | 2019 | *BMJ Open* | Pragmatic cluster randomised double-blind pilot and feasibility trial of an active behavioural physiotherapy intervention for acute non-specific neck pain: A mixed-methods protocol |
| Wootton | 2019 | *BMJ Open* | Telehealth and texting intervention to improve HIV care engagement, mental health and substance use outcomes in youth living with HIV: A pilot feasibility and acceptability study protocol |

| | | | |
|---|---|---|---|
| Yeung | 2019 | *BMJ Open* | Randomised controlled trial to investigate the effectiveness of thoracic epidural and paravertebral blockade in reducing chronic post-thoracotomy pain (TOPIC): A pilot study to assess feasibility of a large multicentre trial |
| Abokhrais | 2018 | *PAFS* | A pilot randomised double blind controlled trial of the efficacy of purified fatty acids for the treatment of women with endometriosis-associated pain (PurFECT): Study protocol |
| Artom | 2019 | *PAFS* | Cognitive-behavioural therapy for the management of inflammatory bowel disease-fatigue: A feasibility randomised controlled trial |
| Aunger | 2019 | *PAFS* | A novel behavioural INTErvention to REduce Sitting Time in older adults undergoing orthopaedic surgery (INTEREST): Protocol for a randomised controlled feasibility study |
| Bérubé | 2019 | *PAFS* | Feasibility of a tapering opioids prescription program for trauma patients at high risk of chronic consumption (TOPPtrauma): Protocol for a pilot randomized controlled trial |
| Bick | 2019 | *PAFS* | Protocol for a two-arm feasibility RCT to support postnatal maternal weight management and positive lifestyle behaviour in women from an ethnically diverse inner city population: The SWAN feasibility trial |
| Bjornstad | 2019 | *PAFS* | Healthy Parent Carers peer-led group-based health promotion intervention for parent carers of disabled children: Protocol for a feasibility study using a parallel group randomised controlled trial design |
| Blanton | 2019 | *PAFS* | A web-based carepartner-integrated rehabilitation program for persons with stroke: Study protocol for a pilot randomized controlled trial |
| Bostrøm | 2019 | *PAFS* | Clinical comparative effectiveness of acupuncture versus manual therapy treatment of lateral epicondylitis: Feasibility randomized clinical trial |
| Bourne | 2019 | *PAFS* | Electrically assisted cycling for individuals with type 2 diabetes mellitus: Protocol for a pilot randomized controlled trial |
| Bowyer-Crane | 2019 | *PAFS* | A randomised controlled feasibility trial and qualitative evaluation of an early years language development intervention: Study protocol of the 'outcomes of Talking Together evaluation and results' (oTTer) project |
| Bryant | 2018 | *PAFS* | Cluster randomised controlled feasibility study of HENRY: A community-based intervention aimed at reducing obesity rates in preschool children |
| Bui | 2019 | *PAFS* | App-based supplemental exercise during inpatient orthopaedic rehabilitation increases activity levels: A pilot randomised control trial |
| Carswell | 2019 | *PAFS* | Implementing an arts-based intervention for patients with end-stage kidney disease whilst receiving haemodialysis: A feasibility study protocol 11 Medical and Health Sciences 1117 Public Health and Health Services 11 Medical and Health Sciences 1103 Clini |
| Clark | 2019 | *PAFS* | Saline versus albumin fluid for extracorporeal removal with slow low efficiency dialysis (SAFER-SLED): Study protocol for a pilot trial |

| | | | |
|---|---|---|---|
| Coe | 2018 | *PAFS* | A protocol for a randomised double-blind placebo-controlled feasibility study to determine whether the daily consumption of flavonoid-rich pure cocoa has the potential to reduce fatigue in people with relapsing and remitting multiple sclerosis (RRMS) |
| Courtier | 2018 | *PAFS* | ACTIVE - A randomised feasibility trial study protocol of a behavioural intervention to reduce fatigue in women undergoing radiotherapy for early breast cancer: Study protocol |
| Cro | 2018 | *PAFS* | Measuring skin necrosis in a randomised controlled feasibility trial of heat preconditioning on wound healing after reconstructive breast surgery: Study protocol and statistical analysis plan for the PREHEAT trial |
| De Oliveira Braga | 2019 | *PAFS* | EMPOWER-PD - A physical therapy intervention to empower the individuals with Parkinson's disease: A study protocol for a feasibility randomized controlled trial |
| Deary | 2018 | *PAFS* | A psychosocial intervention for the management of functional dysphonia: Complex intervention development and pilot randomised trial |
| Ditai | 2019 | *PAFS* | BabyGel pilot: A pilot cluster randomised trial of the provision of alcohol handgel to postpartum mothers to prevent neonatal and young infant infection-related morbidity in the community |
| Downey | 2018 | *PAFS* | Trial of Remote Continuous versus Intermittent NEWS monitoring after major surgery (TRaCINg): Protocol for a feasibility randomised controlled trial |
| Drew | 2019 | *PAFS* | A protocol for a randomised controlled, double-blind feasibility trial investigating fluoxetine treatment in improving memory and learning impairments in patients with mesial temporal lobe epilepsy: Fluoxetine, Learning and Memory in Epilepsy (FLAME trial |
| Duncan | 2018 | *PAFS* | Physical therapy and deep brain stimulation in Parkinson's Disease: Protocol for a pilot randomized controlled trial |
| Dunn | 2019 | *PAFS* | Evaluating Augmented Depression Therapy (ADepT): Study protocol for a pilot randomised controlled trial |
| Fuller | 2018 | *PAFS* | The ACUTE (Ambulance CPAP: Use, Treatment effect and economics) feasibility study: A pilot randomised controlled trial of prehospital CPAP for acute respiratory failure |
| Golla | 2018 | *PAFS* | Home-based balance training using Wii Fit™: A pilot randomised controlled trial with mobile older stroke survivors |
| Hayes | 2019 | *PAFS* | We Can Quit2 (WCQ2): A community-based intervention on smoking cessation for women living in disadvantaged areas of Ireland - Study protocol for a pilot cluster randomised controlled trial |
| Hilari | 2019 | *PAFS* | Adjustment with aphasia after stroke: Study protocol for a pilot feasibility randomised controlled trial for SUpporting wellbeing through PEeR Befriending (SUPERB) |
| Horne | 2019 | *PAFS* | Regaining Confidence after Stroke (RCAS): A feasibility randomised controlled trial (RCT) |
| Jones | 2019 | *PAFS* | Rapid Analgesia for Prehospital hip Disruption (RAPID): Findings from a randomised feasibility study |

| | | | |
|---|---|---|---|
| Kebbe | 2019 | *PAFS* | Feasibility, user experiences, and preliminary effect of Conversation Cards for Adolescents© on collaborative goal-setting and behavior change: Protocol for a pilot randomized controlled trial |
| Kohrt | 2018 | *PAFS* | Reducing stigma among healthcare providers to improve mental health services (RESHAPE): Protocol for a pilot cluster randomized controlled trial of a stigma reduction intervention for training primary healthcare workers in Nepal |
| Lodder | 2019 | *PAFS* | Stigma of living as an autism carer: A brief psycho-social support intervention (SOLACE). Study protocol for a randomised controlled feasibility study |
| Logan | 2018 | *PAFS* | Standing Practice In Rehabilitation Early after Stroke (SPIRES): A functional standing frame programme (prolonged standing and repeated sit to stand) to improve function and quality of life and reduce neuromuscular impairment in people with severe sub-acute stroke-a protocol for a feasibility randomised controlled trial |
| Loughnan | 2019 | *PAFS* | A single-centre, randomised controlled feasibility pilot trial comparing performance of direct laryngoscopy versus videolaryngoscopy for endotracheal intubation in surgical patients |
| Malden | 2019 | *PAFS* | A feasibility cluster randomised controlled trial of a preschool obesity prevention intervention: ToyBox-Scotland |
| McGovern | 2018 | *PAFS* | Promoting Alcohol Reduction in Non- Treatment Seeking parents (PAReNTS): A protocol for a pilot feasibility cluster randomised controlled trial of alcohol screening and brief interventions to reduce parental alcohol use disorders in vulnerable families |
| McIntosh | 2018 | *PAFS* | On the Road to Recovery psychological therapy versus treatment as usual for forensic mental health patients: Study protocol for a randomized controlled feasibility trial |
| Mehta | 2019 | *PAFS* | A randomised controlled feasibility trial to evaluate local heat preconditioning on wound healing after reconstructive breast surgery: The preHEAT trial |
| Meiksin | 2019 | *PAFS* | Protocol for pilot cluster RCT of project respect: A school-based intervention to prevent dating and relationship violence and address health inequalities among young people |
| Milbury | 2018 | *PAFS* | A research protocol for a pilot randomized controlled trial designed to examine the feasibility of a couple-based mind-body intervention for patients with metastatic lung cancer and their partners |
| Milbury | 2019 | *PAFS* | A research protocol for a pilot, randomized controlled trial designed to examine the feasibility of a dyadic versus individual yoga program for family caregivers of glioma patients undergoing radiotherapy |
| Moore | 2018 | *PAFS* | Prehospital recognition and antibiotics for 999 patients with sepsis: Protocol for a feasibility study |
| Morgan | 2019 | *PAFS* | A pilot randomised controlled trial of physical activity facilitation for older adults: Feasibility study findings |
| Morton | 2018 | *PAFS* | Chlorhexidine vaginal preparation versus standard treatment at caesarean section to reduce endometritis and prevent sepsis - A feasibility study protocol (the PREPS trial) |

| | | | |
|---|---|---|---|
| Murphy | 2018 | *PAFS* | Supporting general practitioner-based care for poorly controlled type 2 diabetes mellitus (the DECIDE study): Feasibility study and protocol for a pilot cluster randomised controlled trial |
| Mutedzi | 2019 | *PAFS* | Improving bereavement outcomes in Zimbabwe: Protocol for a feasibility cluster trial of the 9-cell bereavement tool |
| Myers | 2019 | *PAFS* | Accelerometer-based assessment of physical activity within the Fun for Wellness online behavioral intervention: Protocol for a feasibility study |
| Negm | 2018 | *PAFS* | Getting fit for hip and knee replacement: A protocol for the Fit-Joints pilot randomized controlled trial of a multi-modal intervention in frail patients with osteoarthritis |
| Newlands | 2019 | *PAFS* | Pilot randomised controlled trial of Weight Watchers® referral with or without dietitianled group support for weight loss in women treated for breast cancer: The BRIGHT (BReast cancer weIGHT loss) trial |
| O'Regan | 2019 | *PAFS* | An evaluation of an intervention designed to help inactive adults become more active with a peer mentoring component: A protocol for a cluster randomised feasibility trial of the Move for Life programme |
| Paul | 2019 | *PAFS* | Vital sign monitoring with continuous pulse oximetry and wireless clinical notification after surgery (the VIGILANCE pilot study)- A randomized controlled pilot trial |
| Payne | 2018 | *PAFS* | Study protocol for a randomised pilot study of a computer-based, non-pharmacological cognitive intervention for motor slowing and motor fatigue in Parkinson's disease |
| Perman-Howe | 2018 | *PAFS* | The effect of alcohol strength on alcohol consumption: A randomised controlled cross-over pilot trial |
| Philip | 2019 | *PAFS* | A randomised phase II trial to examine feasibility of standardised, early palliative (STEP) care for patients with advanced cancer and their families [ACTRN12617000534381]: A research protocol |
| Pile | 2018 | *PAFS* | A brief early intervention for adolescent depression that targets emotional mental images and memories: Protocol for a feasibility randomised controlled trial (IMAGINE trial) |
| Ponsford | 2018 | *PAFS* | Study protocol for the optimisation, feasibility testing and pilot cluster randomised trial of Positive Choices: A school-based social marketing intervention to promote sexual health, prevent unintended teenage pregnancies and address health inequalities |
| Purcell | 2018 | *PAFS* | Eutectic mixture of local anaesthetics (EMLA®) as a primary dressing on painful chronic leg ulcers: A pilot randomised controlled trial |
| Qurashi | 2019 | *PAFS* | Glycopyrrolate in comparison to hyoscine hydrobromide and placebo in the treatment of hypersalivation induced by clozapine (GOTHIC1): A feasibility study |
| Rowe | 2019 | *PAFS* | A classroom-based intervention targeting working memory, attention and language skills in 4-5 year olds (RECALL): Study protocol for a cluster randomised feasibility trial |

| | | | |
|---|---|---|---|
| Sanfilippo | 2019 | *PAFS* | A study protocol for testing the feasibility of a randomised stepped wedge cluster design to investigate a Community Health Intervention through Musical Engagement (CHIME) for perinatal mental health in the Gambia |
| Sangraula | 2018 | *PAFS* | Protocol for a feasibility study of group-based focused psychosocial support to improve the psychosocial well-being and functioning of adults affected by humanitarian crises in Nepal: Group Problem Management plus (PM+) |
| Schlaeger | 2018 | *PAFS* | Double-blind acupuncture needles: A multi-needle, multi-session randomized feasibility study |
| Schmitz | 2019 | *PAFS* | Impact of endurance exercise and probiotic supplementation on the intestinal microbiota: A cross-over pilot study |
| Shvedko | 2018 | *PAFS* | Physical Activity Intervention for Loneliness (PAIL) in community-dwelling older adults: Protocol for a feasibility study |
| Slobogean | 2019 | *PAFS* | Fixation using alternative implants for the treatment of hip fractures (FAITH-2): Design and rationale for a pilot multi-centre 2 × 2 factorial randomized controlled trial in young femoral neck fracture patients |
| Snowden | 2018 | *PAFS* | Preoperative Behavioural Intervention versus standard care to Reduce Drinking before elective orthopaedic Surgery (PRE-OP BIRDS): Protocol for a multicentre pilot randomised controlled trial |
| Sosnowski | 2018 | *PAFS* | A feasibility study of a randomised controlled trial to examine the impact of the ABCDE bundle on quality of life in ICU survivors |
| Tan | 2019 | *PAFS* | The efficacy of foot orthoses in individuals with patellofemoral osteoarthritis: A randomised feasibility trial |
| Timko | 2018 | *PAFS* | Cognitive remediation therapy (CRT) as a pretreatment intervention for adolescents with anorexia nervosa during medical hospitalization: A pilot randomized controlled trial protocol |
| Totty | 2019 | *PAFS* | Assessing the effectiveness of dialkylcarbamoylchloride (DACC)-coated post-operative dressings versus standard care in the prevention of surgical site infection in clean or clean-contaminated, vascular surgery (the DRESSINg trial): Study protocol for a pilot feasibility randomised controlled trial |
| Volkmer | 2018 | *PAFS* | The 'Better Conversations with Primary Progressive Aphasia (BCPPA)' program for people with PPA (Primary Progressive Aphasia): Protocol for a randomised controlled pilot study |
| Vranceanu | 2019 | *PAFS* | Results of a feasibility randomized controlled trial (RCT) of the Toolkit for Optimal Recovery (TOR): A live video program to prevent chronic pain in at-risk adults with orthopedic injuries |
| Whitehead | 2019 | *PAFS* | HATRIC: A study of Pelargonium sidoides root extract EPs®7630 (Kaloba®) for the treatment of acute cough due to lower respiratory tract infection in adults-study protocol for a double blind, placebocontrolled randomised feasibility trial |
| Wiggins | 2018 | *PAFS* | Testing the effectiveness of REACH Pregnancy Circles group antenatal care: Protocol for a randomised controlled pilot trial |

| | | | |
|---|---|---|---|
| Wong | 2018 | *PAFS* | Thiamine versus placebo in older heart failure patients: Study protocol for a randomized controlled crossover feasibility trial (THIAMINE-HF) |
| Wurz | 2019 | *PAFS* | Exploring the feasibility and acceptability of a mixed-methods pilot randomized controlled trial testing a 12-week physical activity intervention with adolescent and young adult cancer survivors |
| Hilton | 2018 | *PLoS ONE* | Randomised feasibility trial to compare three standard of care chemotherapy regimens for early stage triple-negative breast cancer (REaCT-TNBC trial) |
| Karlsson | 2019 | *PLoS ONE* | Feasibility of preoperative supervised home-based exercise in older adults undergoing colorectal cancer surgery – A randomized controlled design |
| Wiangkham | 2019 | *PLoS ONE* | A cluster randomised, double-blind pilot and feasibility trial of an active behavioural physiotherapy intervention for acute whiplash-associated disorder (WAD)II |
| Ahnfeldt | 2019 | *Trials* | FortiColos - A multicentre study using bovine colostrum as a fortifier to human milk in very preterm infants: Study protocol for a randomised controlled pilot trial |
| Barrett | 2018 | *Trials* | Feasibility of a physical activity programme embedded into the daily lives of older adults living in nursing homes: Protocol for a randomised controlled pilot feasibility study |
| Brennan | 2018 | *Trials* | Prevention of striae gravidarum: Study protocol for a pilot randomised controlled trial |
| Browne | 2019 | *Trials* | Probiotics in pregnancy: Protocol of a double-blind randomized controlled pilot trial for pregnant women with depression and anxiety (PIP pilot trial) |
| Burroughs | 2018 | *Trials* | A feasibility study for NOn-Traditional providers to support the management of Elderly People with Anxiety and Depression: The NOTEPAD study Protocol |
| Cao | 2018 | *Trials* | Aerobic exercise-based cardiac rehabilitation in Chinese patients with coronary heart disease: Study protocol for a pilot randomized controlled trial |
| Chhetri | 2019 | *Trials* | Repetitive vascular occlusion stimulus (RVOS) versus standard care to prevent muscle wasting in critically ill patients (ROSProx):a study protocol for a pilot randomised controlled trial |
| Crawford | 2018 | *Trials* | Psychological Support for Personality (PSP) versus treatment as usual: Study protocol for a feasibility randomized controlled trial of a low intensity intervention for people with personality disorder |
| Deb | 2018 | *Trials* | Aggression Following Traumatic brain injury: Effectiveness of Risperidone (AFTER): Study protocol for a feasibility randomised controlled trial |
| Forster | 2018 | *Trials* | An intervention to support stroke survivors and their carers in the longer term (LoTS2Care): Study protocol for a cluster randomised controlled feasibility trial |

| | | | |
|---|---|---|---|
| Froghi | 2018 | *Trials* | Cardiac output Optimisation following Liver Transplant (COLT) trial: Study protocol for a feasibility randomised controlled trial |
| Greenwood | 2018 | *Trials* | The U&I study: Study protocol for a feasibility randomised controlled trial of a pre-cognitive behavioural therapy digital 'informed choice' intervention to improve attitudes towards uptake and implementation of CBT for psychosis |
| He | 2018 | *Trials* | Xue-Fu-Zhu-Yu capsule in the treatment of qi stagnation and blood stasis syndrome: a study protocol for a randomised controlled pilot and feasibility trial |
| Hutchings | 2018 | *Trials* | CONTRACT Study - CONservative TReatment of Appendicitis in Children (feasibility): Study protocol for a randomised controlled Trial |
| Lee | 2018 | *Trials* | Effect and safety of acupuncture for Hwa-byung, an anger syndrome: A study protocol of a randomized controlled pilot trial |
| Linnemayr | 2018 | *Trials* | Behavioral economics-based incentives supported by mobile technology on HIV knowledge and testing frequency among Latino/a men who have sex with men and transgender women: Protocol for a randomized pilot study to test intervention feasibility and acceptability |
| Littlewood | 2019 | *Trials* | Protocol for a multi-centre pilot and feasibility randomised controlled trial with a nested qualitative study: Rehabilitation following rotator cuff repair (the RaCeR study) |
| Macken | 2018 | *Trials* | Palliative long-term abdominal drains versus repeated drainage in individuals with untreatable ascites due to advanced cirrhosis: Study protocol for a feasibility randomised controlled trial |
| Marsh | 2018 | *Trials* | A novel integrated dressing to secure peripheral intravenous catheters in an adult acute hospital: A pilot randomised controlled trial |
| Marsh | 2018 | *Trials* | Expert versus generalist inserters for peripheral intravenous catheter insertion: A pilot randomised controlled trial |
| Mayo-Wilson | 2019 | *Trials* | Microenterprise intervention to reduce sexual risk behaviors and increase employment and HIV preventive practices in economically-vulnerable African-American young adults (EMERGE): Protocol for a feasibility randomized clinical trial |
| Nymberg | 2018 | *Trials* | Pilot study on increased adherence to physical activity on prescription (PAP) through mindfulness: Study protocol |
| Pace | 2019 | *Trials* | Cognitively-Based Compassion Training versus cancer health education to improve health-related quality of life in survivors of solid tumor cancers and their informal caregivers: Study protocol for a randomized controlled pilot trial |
| Payne Riches | 2019 | *Trials* | The Salt Swap intervention to reduce salt intake in people with high blood pressure: Protocol for a feasibility randomised controlled trial |

| | | | |
|---|---|---|---|
| Poolman | 2019 | *Trials* | CARer-ADministration of as-needed subcutaneous medication for breakthrough symptoms in homebased dying patients (CARiAD): Study protocol for a UK-based open randomised pilot trial |
| Pressman | 2019 | *Trials* | Conducting a pilot randomized controlled trial of community-based mindfulness-based stress reduction versus usual care for moderate-to-severe migraine: Protocol for the Mindfulness and Migraine Study (M&M) |
| Pyle | 2019 | *Trials* | Study protocol for a randomised controlled trial of CBT vs antipsychotics vs both in 14-18-year-olds: Managing Adolescent first episode Psychosis: A feasibility study (MAPS) |
| Russell | 2018 | *Trials* | Feasibility of an online mindfulness-based program for patients with melanoma: Study protocol for a randomised controlled trial |
| Selfe | 2019 | *Trials* | Acceptability and feasibility of a 12-week yoga vs. educational film program for the management of restless legs syndrome (RLS): Study protocol for a randomized controlled trial |
| Taylor | 2019 | *Trials* | Protocol for a randomised controlled feasibility study examining the efficacy of brief cognitive therapy for the Treatment of Anxiety Disorders in Adolescents (TAD-A) |
| Van Oostveen | 2018 | *Trials* | Prevention of Infections in Cardiac Surgery study (PICS): Study protocol for a pragmatic cluster-randomized factorial crossover pilot trial |
| Watt | 2019 | *Trials* | A counseling intervention to address HIV stigma at entry into antenatal care in Tanzania (Maisha): Study protocol for a pilot randomized controlled trial |
| Wright | 2018 | *Trials* | The clinical and cost effectiveness of adapted dialectical behaviour therapy (DBT) for bipolar mood instability in primary care (ThrIVe-B programme): A feasibility study |
| Youssef | 2019 | *Trials* | Addition of a new three-dimensional adjustable cervical thoracic orthosis to a multi-modal program in the treatment of nonspecific neck pain: Study protocol for a randomised pilot trial |
| Zeng | 2019 | *Trials* | Si-ni-tang (a Chinese herbal formula) for improving immunofunction in sepsis: Study protocol for a pilot randomized controlled trial |

*BMJ*: *British Medical Journal*; *PAFS*: *Pilot and Feasibility Studies; PLoS*: *Public Library of Science*
