# Supplementary material for: Progression from external pilot to definitive randomised controlled trial: a methodological review of progression criteria reporting
Source: BMJ Open. 2021 Jun 28;11(6):e048178. doi: 10.1136/bmjopen-2020-048178 (PMC8240572; doi:10.1136/bmjopen-2020-048178)
Supplement: Supplementary data [file bmjopen-2020-048178supp003.pdf]

SUPPLEMENTARY FILE 3: INDICATOR OF FEASIBILITY CONTRIBUTING TO PROGRESSION CRITERIA

Domain	Indicator of feasibility contributing to progression criteria	Number of publications
Management	Data completion or missing data	38
	Barriers or challenges to intervention implementation	7
	Develop or test training materials	4
	Protocol components work together	2
	Data analysis	2
	Data collection methods are suitable/fit for purpose	1
	Data management	1
	Time to Ethics approvals at each site	1
	Time to readiness to initiate the clinical trial	1
Process	Recruitment rates	113
	Retention or attrition rate	106
	Non(compliance) or adherence rate (participants)	66
	Intervention acceptability or evaluation (patients)	33
	Withdrawal or completion rate (trial or intervention)	22
	Intervention acceptability or evaluation (non-patients)	17
	Consent or refusal rate	16
	Randomisation acceptability or rate	15
	Intervention fidelity	15
	Understanding or acceptability of data collection tools/assessments/methods	14
	Non(compliance) or adherence rates (non-participants)	12
	Participant identification or screening	10
	Eligibility rate	9
	Trial acceptability or evaluation (patients)	8
	Characteristics or properties of trial outcome measures	7
	Randomisation adequacy	5
	Crossover or contamination between arms	4
	Enrolment rate	5
	Uptake or engagement rate	3
	Definitive study sample size is achievable	3
	Trial acceptability or evaluation (non-patients)	2
	Success or failure rate	2
	Understanding or acceptability of study instructions	2
	Blinding procedures	1
	Eligibility criteria	1
	Describe control group	2
	Recruitment process	2
	Understanding or acceptability of study information	1
	Allocation concealment	1
	Intervention credibility	1
	Intervention suitability	1
	Completeness of biological sample collection	1
	Interest in using the intervention post-study	1
	Positive expected net gain of sampling from a definitive trial	1

Resources	Centre or investigator recruitment, willingness or capacity	21
	Determining process time	7
	Collection of outcomes relevant to future economic evaluation	4
	Equipment or resource reliability	2
	Venue, location or setting appropriate	1
	Determining capacity	1
	Intervention agreement between methods	1
Scientific	Safety, adverse events, unintended consequences or harms	25
	Estimate of treatment effect	11
	Estimate of variance of treatment effect	6
	Patient response	5
	Signal of efficacy	4
	Estimate intracluster correlation coefficient	1
	Context and mechanisms of action	1
	Intervention tolerability	1
