# Supplementary material for: Progression from external pilot to definitive randomised controlled trial: a methodological review of progression criteria reporting
Source: BMJ Open. 2021 Jun 28;11(6):e048178. doi: 10.1136/bmjopen-2020-048178 (PMC8240572; doi:10.1136/bmjopen-2020-048178)
Supplement: Author's manuscript [file bmjopen-2020-048178.draft_revisions.pdf]

BMJ Open is committed to open peer review. As part of this commitment we make the peer review history of every article we publish publicly available.

When an article is published we post the peer reviewers' comments and the authors' responses online. We also post the versions of the paper that were used during peer review. These are the versions that the peer review comments apply to.

The versions of the paper that follow are the versions that were submitted during the peer review process. They are not the versions of record or the final published versions. They should not be cited or distributed as the published version of this manuscript.

BMJ Open is an open access journal and the full, final, typeset and author-corrected version of record of the manuscript is available on our site with no access controls, subscription charges or pay-per-view fees (http://bmjopen.bmj.com).

If you have any questions on BMJ Open's open peer review process please email info.bmjopen@bmj.com

BMJ Open

Progression from external pilot to definitive randomised controlled trial are not adequately reported: A methodological review of progression criteria reporting

Journal:	*BMJ Open*
Manuscript ID	bmjopen-2020-048178
Article Type:	Original research
Date Submitted by the Author:	04-Jan-2021

Complete List of Authors:	Mellor, Katie; University of Oxford, Centre for Statistics in Medicine, Nuffield Department of Orthopaedics, Rheumatology and Musculoskeletal Sciences<br>Eddy, Saskia; Queen Mary University of London, Institute of Population Health Sciences, Barts and the London School of Medicine and Dentistry<br>Peckham, Nicholas; University of Oxford, Centre for Statistics in Medicine, Nuffield Department of Orthopaedics, Rheumatology and Musculoskeletal Sciences<br>Bond, Christine; University of Aberdeen, Institute of Applied Health Sciences<br>Campbell, Michael; The University of Sheffield, School of Health and Related Research<br>Lancaster, Gillian; Keele University, School of Medicine & Keele Clinical Trials Unit (CTU)<br>Thabane, Lehana ; McMaster University, Department of Health Research Methods, Evidence and Impact<br>Eldridge, Sandra; Queen Mary University of London, Institute of Population Health Sciences, Barts and the London School of Medicine and Dentistry<br>Dutton, Susan; University of Oxford, Centre for Statistics in Medicine, Nuffield Department of Orthopaedics, Rheumatology and Musculoskeletal Sciences<br>Hopewell, Sally; University of Oxford, Centre for Statistics in Medicine, Nuffield Department of Orthopaedics, Rheumatology and Musculoskeletal Sciences

Keywords:	STATISTICS & RESEARCH METHODS, Protocols & guidelines < HEALTH SERVICES ADMINISTRATION & MANAGEMENT, Clinical trials < THERAPEUTICS

SCHOLARONE™
Manuscripts

I, the Submitting Author has the right to grant and does grant on behalf of all authors of the Work (as defined in the below author licence), an exclusive licence and/or a non-exclusive licence for contributions from authors who are: i) UK Crown employees; ii) where BMJ has agreed a CC-BY licence shall apply, and/or iii) in accordance with the terms applicable for US Federal Government officers or employees acting as part of their official duties; on a worldwide, perpetual, irrevocable, royalty-free basis to BMJ Publishing Group Ltd ("BMJ") its licensees and where the relevant Journal is co-owned by BMJ to the co-owners of the Journal, to publish the Work in this journal and any other BMJ products and to exploit all rights, as set out in our licence.

The Submitting Author accepts and understands that any supply made under these terms is made by BMJ to the Submitting Author unless you are acting as an employee on behalf of your employer or a postgraduate student of an affiliated institution which is paying any applicable article publishing charge ("APC") for Open Access articles. Where the Submitting Author wishes to make the Work available on an Open Access basis (and intends to pay the relevant APC), the terms of reuse of such Open Access shall be governed by a Creative Commons licence – details of these licences and which Creative Commons licence will apply to this Work are set out in our licence referred to above.

Other than as permitted in any relevant BMJ Author's Self Archiving Policies, I confirm this Work has not been accepted for publication elsewhere, is not being considered for publication elsewhere and does not duplicate material already published. I confirm all authors consent to publication of this Work and authorise the granting of this licence.

For peer review only

BMJ Open manuscript draft v1.0_18Dec2020

Progression from external pilot to definitive randomised controlled trial are not adequately reported: A methodological review of progression criteria reporting

Authors

Katie Mellor[1], Saskia Eddy[2], Nicholas Peckham[1], Christine M Bond[3], Michael J Campbell[4], Gillian A Lancaster[5], Lehana Thabane[6], Sandra M Eldridge[2], Susan J Dutton[1], Sally Hopewell[1]

Author information

[1]Centre for Statistics in Medicine, Nuffield Department of Orthopaedics, Rheumatology and Musculoskeletal Sciences, University of Oxford, Oxford, England

[2]Institute of Population Health Sciences, Barts and the London School of Medicine and Dentistry, Queen Mary University of London, London, England

[3]Institute of Applied Health Sciences, University of Aberdeen, Aberdeen, Scotland

[4]School of Health and Related Research, University of Sheffield, Sheffield, England

[5]School of Medicine & Keele Clinical Trials Unit (CTU), Keele University, Newcastle under Lyme, England

[6]Department of Health Research Methods, Evidence and Impact, McMaster University, Hamilton, Ontario, Canada

Authors emails

katie.mellor@ndorms.ox.ac.uk*

s.eddy@qmul.ac.uk

nicholas.peckham@ndorms.ox.ac.uk

c.m.bond@abdn.ac.uk

m.j.campbell@sheffield.ac.uk

g.Lancaster@keele.ac.uk

ThabanL@mcmaster.ca

s.eldridge@qmul.ac.uk

susan.dutton@csm.ox.ac.uk

sally.hopewell@csm.ox.ac.uk

*Corresponding author

For peer review only - http://bmjopen.bmj.com/site/about/guidelines.xhtml

ABSTRACT

Objectives: Prespecified progression criteria can inform the decision to progress from an external randomised pilot trial to a definitive randomised controlled trial. We assessed the characteristics of progression criteria reported in external randomised pilot trial protocols and results publications, including whether progression criteria were specified a priori and mentioned in prepublication peer review reports.

Study design: Methodological review.

Methods: We searched four journals through PubMed: *British Medical Journal Open, Pilot and Feasibility Studies, Trials and Public Library of Science One*. Eligible publications reported external randomised pilot trial protocols or results, were published between January 2018 and December 2019, and reported progression criteria. We double-data extracted 25% of the included publications. Here we report the progression criteria characteristics.

Results: We included 160 publications (123 protocols and 37 completed trials). Recruitment and retention were the most frequent indicators contributing to progression criteria. Progression criteria were mostly reported as distinct thresholds (e.g., achieving a specific target; 133/160, 83%). Less than a third of the planned and completed pilot trials that included qualitative research reported how these findings would contribute towards progression criteria (34/108, 31%). The publications seldom stated who established the progression criteria (12/160, 7.5%) or provided rationale or justification for progression criteria (44/160, 28%). Most completed pilot trials reported the intention to proceed to a definitive trial (30/37, 81%), but less than half strictly met all of their progression criteria (17/37, 46%). Prepublication peer review reports were available for 153/160 publications (96%). Peer review reports for 86/153 (56%) publications mentioned progression criteria, with peer reviewers of 35 publications commenting that progression criteria appeared not to be specified.

Conclusions: Many external randomised pilot trial publications did not adequately report or propose prespecified progression criteria to inform whether to proceed to a future definitive randomised controlled trial.

Review registration: Open Science Framework osf.io/bn35k

For peer review only

For peer review only - http://bmjopen.bmj.com/site/about/guidelines.xhtml

STRENGTHS AND LIMITATIONS OF THIS STUDY

- We conducted a large, recent assessment of the use and reporting of progression criteria in publications reporting external randomised pilot trial protocols and results.
- As this study only investigated external randomised pilot trial publications, it is unclear whether the findings can be generalised to other external feasibility study designs such as non-randomised pilot trials and non-pilot feasibility studies
- One researcher independently screened all publications, assessed eligibility, and extracted data from all included publications, while other members of the research team provided a second data extraction for 25% of the included publications.

For peer review only

For peer review only - http://bmjopen.bmj.com/site/about/guidelines.xhtml

INTRODUCTION

Pilot trials aim to determine whether a future definitive randomised controlled trial (RCT) is feasible.[1] Internal pilot trials are embedded in the RCT design forming its first phase.[2] In contrast, external pilot trials are small stand-alone studies conducted before a definitive RCT. Prespecified progression criteria can help researchers interpret the findings of an external randomised pilot to decide whether the future definitive RCT is or is not feasible, and whether changes should be made to the trial design. Progression criteria should be specified before the pilot trial begins (a priori) to avoid introducing bias associated with establishing progression criteria once external pilot trial findings are known.

A 2019 review found that less than 20% of randomised pilot trial protocols published between 2013 and 2017 reported clear progression criteria. Trial features, such as a more recent publication year and certain countries of origin were associated with reporting progression criteria.[3] The 2016 Consolidated Standards of Reporting Trials (CONSORT) extension for reporting randomised pilot and feasibility trials advises that "at a minimum there should be something reported to suggest how the decision to progress to the definitive study will be made".[4] The extent to which this guidance has improved progression criteria reporting in more recently published pilot trials is unclear. Although previous research has investigated whether progression criteria are reported, however the quality of progression criteria reporting – including how the criteria are established during pilot trial design and assessed upon pilot trial completion – has not yet been investigated.

We conducted a methodological review to investigate the application and reporting of progression criteria in a recent sample of external randomised pilot trial protocol and results publications. The primary objective was to describe the reporting of progression criteria, including the areas of feasibility that progression criteria were based on as described in a published framework of reasons for conducting pilot trials[5], their rationale or justification, and who established and assessed the progression criteria. One set of secondary objectives were to assess whether the progression criteria reported in pilot trial results publications were specified a priori in a published protocol or trial registration and whether the results publication reported the intention to progress to a definitive RCT. We also assessed the extent and context in which progression criteria were discussed in prepublication peer review reports where available for the protocol and results publications.

METHODS

Protocol and registration

A protocol for this research is registered on the Open Science Framework: osf.io/bn35k.[6] A summary of the methods used is detailed below. This review is reported following the PRISMA statement.[7]

Eligibility criteria

We included all protocol and results publications for external randomised pilot trials, that reported progression criteria and were published between January 2018 and December 2019 inclusive. Progression criteria were defined as criteria to inform the decision to progress to a definitive RCT. Included publications were published in the English language and were not restricted by intervention, health-related context or setting.

Information sources

For peer review only - http://bmjopen.bmj.com/site/about/guidelines.xhtml

Four journals were searched through PubMed: *British Medical Journal (BMJ) Open, Pilot and Feasibility Studies (PAFS), Trials and Public Library of Science (PLoS) One*. These journals were chosen because they are known to publish pilot trial protocol and results publications and had published the most PubMed indexed publications that included the terms 'pilot' or 'feasibility and 'trial' or 'protocol' in their title within 2018 and 2019. All included journals direct authors to the CONSORT statement[8] reporting guideline: BMJ Open and Trials advise authors to use the most appropriate statement extension, and the PAFS journal directs authors to the CONSORT Extension to Pilot and Feasibility Trials.[4]

Search terms included 'pilot' or 'feasibility' in the title, and 'trial', 'study' or 'protocol' in the title or abstract. See Supplementary file 1 for the full search strategy which was last used on 6 January 2020.

Study selection

Titles and abstracts of identified publications were screened against inclusion criteria. Full texts were retrieved for those that appeared relevant and screened against a predefined eligibility checklist by KM. All included publications were saved in EndNote X9 for Windows. Where both the protocol and corresponding pilot trial result publication were identified both were included.

Data collection

Data extraction forms produced in Microsoft Excel (Office16) were pre-piloted on the first 10 trials ordered alphabetically to ensure usability and completeness (the data extraction form used can be obtained from osf.io/fxv4n). One researcher (KM) extracted the data for all included publications. Other team members (SEd and NP) conducted a second data extraction for a randomly selected 25% sample. As we found minimal differences between the two data extractions, we decided not to conduct double data extraction for all of the included publications.

From trial protocol and results publications, we extracted: trial characteristics (including author, year, journal, country, randomisation design, therapeutic area, intervention type, sample size target, number of arms, and single or multicentre); feasibility objectives, outcomes, and instances of hypothesis testing; progression criteria details (wording, rationale or justification, format, process for establishing, and process for assessing); and references to progression criteria in prepublication peer review reports, where these were published online and linked to the publication.

For completed pilot trial results publications, we also extracted: whether progression criteria were met; any reported intention to progress to a definitive RCT; any proposed changes to the definitive RCT design; any refinement of hypotheses; any comment on data quality; and whether progression criteria had changed from the corresponding protocol or trial registration publication, if a published protocol was not available.

Synthesis of results

Descriptive statistics (frequencies and the mean, median and interquartile range for trial sample sizes) were produced to describe trial characteristics and address our primary and secondary objectives. Data were analysed using Stata version 15.0, StataCorp.[9] We report the frequency with which different feasibility uncertainties contributed to progression criteria using pre-specified domains of reasons for conducting pilot trials: process, resource, management and scientific.[5] The mean number of progression criteria specified per trial was also calculated.

We used narrative synthesis to describe the context in which progression criteria were mentioned in publicly available prepublication peer reviewer reports (synthesised by KM). We did not use a

For peer review only - http://bmjopen.bmj.com/site/about/guidelines.xhtml

predefined checklist to formally assess peer reviewer reports and we do not comment on the quality of peer review. Instead we simply looked for any mention that progression criteria were not present in the prepublication manuscript, and any queries about rationale for progression criteria used.

We did not aim to comment on the quality of the evidence from the studied randomised pilot trials. We aimed to comment only on the quality of reporting of progression criteria in this sample of external randomised pilot trial protocol and results publications.

Patient and public involvement

Patients or the public were not involved in the design, conduct, reporting, or dissemination plans of this research.

RESULTS

Study selection

Our search strategy identified 1030 publications. We screened their titles and abstracts, then assessed the full texts of 679 publications for eligibility. One hundred and sixty publications were eligible for our study. Figure 1 shows the full PRISMA flow chart of publications included and excluded at each stage. Supplementary file 2 lists the included publications describing external pilot trial protocols and results. We found two instances where both the completed trial publication and protocol were identified. In these instances, both were included.

Study characteristics

Table 1 summarises the characteristics of the included publications. Most of the publications were pilot trial protocols (123/160, 77%) rather than completed pilot trial results (37/160, 23%). The journal with the most eligible publications was *Pilot and Feasibility Studies* (77/160, 48%). Most publications described external pilot trials that were two-arm (143/160, 89%), multicentre (102/160, 64%), non-industry funded (147/160, 92%) trials of counselling, lifestyle, or physiotherapy interventions (94/160, 59%). The reported trials covered 27 therapeutic areas and trials were from 18 countries, mostly from the UK (87/160, 54%).

Primary feasibility objectives were explicitly stated in 71/160 (44%) publications, and most publications reported feasibility outcomes in the methods that addressed all of the stated feasibility objectives (109/160, 68%). In 50/160 (31%) of the publications, the stated feasibility outcomes only somewhat addressed trial objectives, often because the objective stated was broad (e.g., "to determine whether a future trial is feasible") and did not define specific aspects of feasibility being assessed. With respect to data collection and assessment of feasibility outcomes, completely defined prespecified assessments or measurements were often stated (140/160, 88%). Most of the pilot trial publications that reported the intention to conduct hypothesis testing stated that this was exploratory or advised caution in interpretation. All but one publication reported multiple feasibility outcomes. The place in the publication where the specific uncertainties related to trial feasibility were first reported varied, but most often this was within the pilot trial feasibility objectives (72/160, 45%), or within the data collection section describing the feasibility outcomes (26/160, 16%) or pilot trial assessments or measurements (23/160, 14%).

Characteristics of progression criteria

For peer review only - http://bmjopen.bmj.com/site/about/guidelines.xhtml

Characteristics of progression criteria are presented in Table 2. The reported progression criteria generally addressed some (99/160, 62%) or all (53/160, 33%) of the pilot trial's feasibility outcomes. The pilot trial publications reported a mean of 4 (mean 4.05) progression criteria targets per pilot trial. Recruitment (113/160) and retention (106/160) were the most commonly reported indicators of feasibility to inform progression criteria. In total we identified 58 distinct areas of trial feasibility that contributed to progression criteria, which we grouped into four domains: process, resource, management and scientific. The domains and areas are listed in Supplementary file 3. Most of the areas were process uncertainties (34/58, 59%), which dealt with the feasibility of processes that are key to the success of the future definitive RCT.[5]

Four publications reported progression criteria that were based on detecting potential efficacy, including determining non-inferiority of the intervention compared to a comparator, determining intervention superiority at follow-up, and finding a trend for difference between the intervention and comparator groups on clinical outcomes.

Progression criteria and quantitative indicators of feasibility

All of the pilot trial protocol and result publications reported using quantitative indicators of trial feasibility (e.g., rate of recruitment and amount of missing data) to inform at least one of the trial's progression criteria, with 78% (125/160) basing all progression criteria on quantitative indicators.

All but seven publications reported quantifiable numerical thresholds that were, or would be, used to assess the progression criteria. The seven remaining publications did not report specific quantifiable targets for progression criteria, but did report how the decision to progress from pilot to definitive RCT would be made and the feasibility indicators that would be considered when making this decision.[10]

The quantifiable numerical targets used were most often reported as a distinct threshold (e.g., achieving a specified rate of recruitment, retention or data completion) (133/160, 83%). This was followed by a traffic light approach to reporting progression criteria (20/160, 13%) with thresholds correlating to different domains (e.g. above a higher threshold (green) indicating the definitive trial is feasible/proceed, within a mid/acceptable threshold (amber) indicating that changes to definitive trial are required, and below a lower threshold (red) indicating that the definitive trial is not feasible/not proceed). Figure 2 shows an example of the traffic light approach.[11]

Progression criteria and qualitative indicators of feasibility

Many publications reported planned or completed qualitative research as part of the randomised pilot trial (108/160, 68%). Although the findings from qualitative research conducted as part of a pilot trial are often reported in a separate publication, the intention to conduct qualitative research as part of a pilot trial should be included in the pilot trial protocol. The intention to conduct qualitative research was reported in protocols (89/123, 72%) more often than the results of qualitative research were reported in pilot trial result publications (19/37, 51%). However, qualitative indicators of trial feasibility, such as participants' or researchers' views of the acceptability of the trial or intervention collected in interviews, only informed progression criteria in 34 of the 108 (31%) publications that reported planned or completed qualitative research.

Two protocols reported multiple progression criteria for individual feasibility indicators, for example reporting both a target for number of participants recruited, and another target for number of participants recruited in a given timeframe. In one of these instances the authors reported that all criteria would need to be met or met within reasonable limits (within the green or amber traffic light

For peer review only - http://bmjopen.bmj.com/site/about/guidelines.xhtml

domain) to progress to a full trial without major study re-design. It was unclear in the other protocol whether meeting one criterion for each indicator of feasibility was sufficient justification for progression.

Process for establishing progression criteria

12 pilot trial publications reported how the progression criteria had been established, with most involving a trial steering or oversight committee (10/12, 83%), working with funders (3/12, 25%) and/or a trial management group (5/12, 42%). Other examples included agreeing progression criteria with a data monitoring and ethics committee (1/12, 8%) or study physicians (1/12, 8%) or establishing progression criteria based on clinical experience (1/12, 8%).

Forty-four of the 160 publications (28%) reported rationale or justification for all or some of the stated progression criteria. For 29 publications the stated justification was previous related research, with 25 providing references to previous studies. Thirteen publications referenced sources of guidance and methodological research,[4,10,12–21] including three references to published guidance for internal pilot trials.[2] Four publications reported that contextual considerations had informed progression criteria (such as what would be an achievable recruitment rate, or intervention timeframe in the definitive trial), and three reported that clinical considerations had informed criteria (including medical chart reviews, clinical advice, and the nature of the population). Most of the pilot trial publications (116/160, 73%) did not report any rationale or justification for choice of progression criteria.

Process for assessing progression criteria

Nearly half of the publications (74/160, 46%) reported how progression criteria had or would inform the decision to progress to a future definitive RCT. This included whether changes to definitive RCT design would be considered if criteria were not strictly met (e.g., were met within reasonable limits or within the aforementioned "amber" traffic light range), or who was or would be involved in assessing progression criteria.

One publication reported a two-stage decision making process with different criteria assessed at each stage. Stage 1 was to decide on the best intervention route, and stage 2 was to decide whether to take the optimal intervention route forward to a definitive RCT. Another publication described the intention to hold a consensus conference of key stakeholders (patients, surgeons, public representatives and researchers) to agree whether a definitive RCT was feasible. Four pilot trials referred to the ADePT framework[10] to facilitate progression decision-making.

Nearly a quarter of publications reported who would be involved in assessing progression criteria (35/160, 22%). An independent trial steering committee was most commonly involved (26/35, 74%). Other reported parties included the research team or trial management group (13/35, 37%), data monitoring committee (7/35, 20%), trial sponsor (2/35, 6%), funder (1/35 3%), independent statistician (1/35, 3%), and other stakeholders, such as patients, clinicians and public representatives (3/35, 9%).

Intentions of completed randomised pilot trial publications

Most completed pilot trials reported that a future RCT would be feasible or the intention to proceed (30/37, 81%), including the seventeen completed pilot trials which met all of their progression criteria (Table 3). Thirteen pilot trials met some of their progression criteria, of these nine reported that a future RCT would be feasible, two reported that they would not proceed to a definitive RCT and two reported the intention to conduct further feasibility assessment. Four pilot trials did not meet their

For peer review only - http://bmjopen.bmj.com/site/about/guidelines.xhtml

progression criteria, of which three reported that a future RCT would still be feasible with changes to study design. The extent to which progression criteria was met was unclear for three trials, of these two reported the intention to conduct further feasibility assessment, and one reported that a future RCT would be feasible.

All but two of the completed pilot trials that reported that a future RCT would be feasible planned to make changes to their definitive RCT design (28/30, 93%). Of these, four reported the implications of the pilot trial findings in a table format, alongside whether progression criteria had or had not been met. Proposed changes included altering eligibility criteria, recruitment strategies (e.g. number of sites, recruitment materials, recruitment setting), randomisation design, blinding, outcome measures, follow up schedules and duration and seeking additional research team support (such as a dedicated trial manager, research coordinator and administrative team). It was unclear for two pilot trials whether changes would be made.

Four pilot trials reported definitive RCT funding intentions: Two NIHR Health Technology Assessment, one European and Developing Countries Clinical Trials Partnership, and one reported that a funding application had been prepared and submitted but did not specify the funder. One trial reported an anticipated progression timeframe, specifying a recruitment start year for the definitive RCT.

A priori progression criteria reporting of included randomised pilot trial publications

Trial protocols were available for 16 of the 37 completed randomised pilot trial publications (Table 3). Trial registrations were identified for 20 of the trials without a published protocol. We were unable to identify a published protocol or trial registration for the one remaining completed pilot trial.

Twenty-two published protocols or trial registrations for the 37 included completed pilot trials did not report progression criteria. An additional four protocols or trial registrations reported different progression criteria to the pilot trial result publication. Only one completed trial publication explained why the progression criteria had changed from the protocol: as the qualitative findings were reported in a separate publication, the progression criteria associated with acceptability were not included in the completed pilot trial result publication.

Progression criteria in prepublication peer reviewer reports

Prepublication peer review reports were available for 153 of the 160 included external pilot publications. Peer reviewer reports were not publicly available for the three *PLoS One* publications and peer review was not commissioned for four of the pilot trial protocols published in *BMJ Open*.

Table 2 shows that over half of the prepublication peer reviewer reports commented on progression criteria (86/153, 56%). Peer reviewer reports for 35 pilot trial publications (6 completed, 29 protocol) indicated that progression criteria were not reported in the submitted prepublication manuscript. Whether progression criteria were reported in the submitted prepublication manuscript was unclear for another five pilot trial publications.

Peer reviewer reports for 20 of the publications referred to the rationale or justification given for progression criteria. For example, they asked why a specific progression criterion was set, why progression criteria were given for specific outcomes, how the progression criteria were established, and how the progression decision was or will be made.

Peer reviewer reports for 26 pilot trials publications mentioned other aspects of progression criteria. For example, they mentioned changing where the progression criteria were reported in the manuscript (such as including the progression criteria in the publication abstract and not solely within

For peer review only - http://bmjopen.bmj.com/site/about/guidelines.xhtml

a supplementary file), clarifying ambiguous wording, adding percentages in brackets for clarity, correcting inconsistencies in the manuscript, and clarifying how specific criteria will be assessed. Reviewers also complemented authors for describing progression criteria well.

Not every author opted to update or add progression criteria to their manuscript after prepublication peer review. The authors of one publication argued that they could not alter their progression criteria because these criteria had been agreed by the trial management group, trial steering committee, and ethics committee. Other reasons that authors gave in response to peer review for not reporting quantifiable numerical targets for progression criteria included: they were not set during trial design; strict thresholds might be influenced by contextual variations that may not affect a future trial; progression criteria are best viewed as guidelines in line with the CONSORT extension statement; different perspectives could not be successfully captured by a set of criteria, and the trial is not an internal pilot.

DISCUSSION

Summary of main findings

Our study provides an assessment of the reporting of progression criteria in a large sample of external randomised pilot trials. We found that progression criteria varied widely and were not often justified, which agrees with recent research assessing the use of progression criteria in internal pilot trials.[22,23] Like internal pilots, many of the studied external randomised pilot trials also reported the intention to proceed to a definitive RCT even when they had not strictly met all progression criteria. It was also unclear within the studied publications how, or by who, progression criteria are established and assessed.

Our findings suggest that guidance is needed to facilitate transparent and complete reporting of progression criteria a priori in external pilot trial protocols[3] and in pilot trial results publications.[24]

Strengths and weaknesses of the study

A study strength is our extensive recent sample of 160 publications reporting external randomised pilot trials in four key journals that are known to publish pilot trials.

A limitation of this study is that single screening was used, and double data extraction was only conducted for 25% of the included publications. However, since only minimal data extraction differences were identified we decided not to conduct double data extraction for all included publications.

We only included external randomised pilot trials and it is unclear whether these findings are generalisable to other external feasibility study designs, such as non-randomised pilot trials and non-pilot feasibility studies.

In addition, our review of peer reviewer reports to assess the context in which progression criteria were mentioned was subject to interpretation. Prepublication peer review reports were not available for all included publications: *PLoS One* allows authors to opt-in to publish peer review reports, and peer review was not commissioned for four pilot trial protocols published in *BMJ Open* that had already been peer reviewed for ethical and funding approval before submission. Progression criteria might also have been added or altered based on editorial review before peer review. Unlike peer reviewer reports, it is not common practice to make editorial review publicly available.

For peer review only - http://bmjopen.bmj.com/site/about/guidelines.xhtml

Meaning of the study: Possible explanations and implications for clinicians and policymakers

Our findings suggest that the research community are uncertain about how progression criteria should be applied to external randomised pilot trials and how this should be reported in protocol and results publications. We identified one instance within a peer reviewers report for a pilot trial protocol where authors had stated that progression criteria were not set because the trial was not an internal pilot and as such would not immediately progress to a fully powered RCT.

We found recruitment and retention rates to be the most common feasibility uncertainties to contribute towards progression criteria. This result is supported by a recent review finding recruitment to be the most common uncertainty evaluated in surgical pilot and feasibility studies,[25] and is unsurprising considering that recruiting to target is a challenge for many RCTs.[26] Fairhurst and colleagues suggested that researchers conducting feasibility studies might focus on feasibility uncertainties that are perceived to be important to funders.[25] In support of this suggestion, we found that other feasibility uncertainties that are equally as important to trial success, such as intervention acceptability, contributed to progression criteria much less often than recruitment and retention.

We found that peer review improved the reporting of pilot trials, for example by prompting authors to explain their progression criteria and rationale. However, we also identified instances where new progression criteria were likely added as a result of peer review, in both protocols and pilot trial result manuscripts. Adding post-hoc progression criteria, could introduce bias since progression criteria might be based on targets that have been met or exceeded to justify progression to a definitive RCT.

Unanswered questions and future research

The processes for establishing and assessing progression criteria were not commonly reported, leaving unanswered questions about how the decision to progress from pilot to definitive RCT is made in practice. The views of researchers who design and conduct external randomised pilot trials are required to understand these processes. Our findings also highlight the importance of journal editor and peer reviewer endorsement of evidence-based guidelines to improve reporting standards. The development of evidence-based guidance specific to the application and reporting of progression criteria in external pilot trials, for both protocols and completed trials, is a research priority. This finding is timely, as the UK's biggest funder of pilot and feasibility studies, NIHR Research for Patient Benefit, now stipulates that a clear route of progression criteria should be included in pilot and feasibility study funding applications.[27] A further possible area of investigation is whether research ethics committees can and should comment on progression criteria in research ethics applications. Researchers have an ethical obligation to conduct research with integrity and transparency. Defining a priori progression criteria and adequately reporting them helps to uphold the integrity and transparency of the external randomised pilot trial's progression.

Conclusions

We found heterogeneity in the reporting of progression criteria in external randomised pilot trial publications. It was often unclear how progression criteria were established, upon what justification or rationale they were based, how they were or will be assessed, and who is involved at each stage. Peer reviewers often commented on progression criteria, questioning whether these criteria were established a priori, as is recommended for good practice. Clear, evidence-based recommendations for the use and reporting of progression criteria in external randomised pilot trials is required. Guidance to this effect would benefit researchers, peer reviewers, journal editors, and funders of external randomised pilot trials, and inform the design of subsequent definitive trials.

For peer review only - http://bmjopen.bmj.com/site/about/guidelines.xhtml

ACKNOWLEDGEMENTS

We acknowledge Dr Jennifer A de Beyer, Centre for Statistics in Medicine, University of Oxford for English language editing.

FOOTNOTES

Twitter: @katiejmellor

Contributors: This study was conceived and designed as part of KMs doctoral thesis under the supervision of SH, SD and SE. CB, MC, GL and LT provided feedback on the study design during protocol development. KM collected and analysed the data. SEd and NP conducted double data extraction. KM drafted the manuscript. All authors reviewed and commented on manuscript drafts. All authors approved the final manuscript.

Funding: This work was supported by Medical Research Council Doctoral Training Partnership funding awarded to KM.

Competing interests: KM, SEd, CB, MC, GL, LT, SE and SH contribute to a Pilot and Feasibility Studies working group. GL and LT are Editors in Chief of the Pilot and Feasibility Studies journal. SE, MC and CB are editors of the Pilot and Feasibility Studies journal.

Data sharing: Data will be made available upon request and included in a DPhil thesis published open access through the Oxford University Archive upon KM's DPhil completion.

For peer review only - http://bmjopen.bmj.com/site/about/guidelines.xhtml

FIGURES AND TABLES

Figure 1 PRISMA flow chart

Figure 2 Example of the traffic light system for progression criteria. Table 3 reproduced from Ahnfeldt AM, Hyldig N, Li Y, et al. *Trials* 2019;20:279 (CC BY 4.0)

For peer review only

For peer review only - http://bmjopen.bmj.com/site/about/guidelines.xhtml

Table 1 Characteristics of the studied external randomised pilot trial publications

	Completed (n=37) n (%)	Protocol (n=123) n (%)	Total (n=160) n (%)
**Journal**			
British Medical Journal (BMJ) Open	11 (30%)	34 (28%)	45 (28%)
Pilot and Feasibility Studies (PAFS)	21 (57%)	56 (46%)	77 (48%)
Trials	2 (5%)	33 (27%)	35 (22%)
Public Library of Science (PLoS) One	3 (8%)	0 (0%)	3 (2%)
**Country**			
Australia	6 (16%)	4 (3%)	10 (6%)
Brazil	0 (0%)	1 (1%)	1 (1%)
Canada	4 (11%)	15 (12%)	19 (12%)
China	0 (0%)	4 (3%)	4 (3%)
Denmark	0 (0%)	1 (1%)	1 (1%)
Germany	1 (3%)	1 (1%)	2 (1%)
Korea	0 (0%)	1 (1%)	1 (1%)
Nepal	1 (3%)	2 (2%)	3 (2%)
New Zealand	2 (5%)	1 (1%)	3 (2%)
Norway	1 (3%)	0 (0%)	1 (1%)
Republic of Ireland	0 (0%)	5 (4%)	5 (3%)
Sweden	1 (3%)	1 (1%)	2 (1%)
Tanzania	0 (0%)	1 (1%)	1 (1%)
Thailand	0 (0%)	1 (1%)	1 (1%)
The Netherlands	0 (0%)	2 (2%)	2 (1%)
UK	19 (51%)	68 (55%)	87 (54%)
USA	2 (5%)	14 (11%)	16 (10%)
Zimbabwe	0 (0%)	1 (1%)	1 (1%)
**Funder**			
Industry	2 (5%)	2 (2%)	4 (3%)
Non-industry	32 (86%)	115 (94%)	147 (92%)
A combination	1 (3%)	4 (3%)	5 (3%)
Unknown	2 (5%)	1 (1%)	3 (2%)
Trial did not receive funding	0 (0%)	1 (1%)	1 (1%)
**Therapeutic areas**			
Alternative medicine	0 (0%)	1 (1%)	1 (1%)
Anaesthesia	1 (3%)	1 (1%)	2 (1%)
Cardiology	0 (0%)	3 (2%)	3 (2%)
Critical Care	1 (3%)	7 (6%)	8 (5%)
Endocrinology	0 (0%)	6 (5%)	6 (4%)
Gastroenterology	1 (3%)	3 (2%)	4 (3%)
Geriatrics	1 (3%)	4 (3%)	5 (3%)
Hepatology	0 (0%)	1 (1%)	1 (1%)
Infectious diseases	0 (0%)	3 (2%)	3 (2%)
Musculoskeletal	6 (16%)	4 (3%)	10 (6%)
Nephrology	0 (0%)	1 (1%)	1 (1%)
Neurology	3 (8%)	12 (10%)	15 (9%)
Obstetrics/Gynaecology	2 (5%)	6 (5%)	8 (5%)
Oncology	4 (11%)	7 (6%)	11 (7%)
Ophthalmology	0 (0%)	1 (1%)	1 (1%)
Orthopaedics	2 (5%)	1 (1%)	3 (2%)

For peer review only

For peer review only - http://bmjopen.bmj.com/site/about/guidelines.xhtml

Other	2 (5%)	3 (2%)	5 (3%)
Otolaryngology (ENT)	2 (5%)	1 (1%)	3 (2%)
Paediatrics	2 (5%)	3 (2%)	5 (3%)
Pain	1 (3%)	0 (0%)	1 (1%)
Palliative care	0 (0%)	3 (2%)	3 (2%)
Psychiatry/Psychology	2 (5%)	19 (15%)	21 (13%)
Public Health	2 (5%)	15 (12%)	17 (11%)
Respiratory	0 (0%)	2 (2%)	2 (1%)
Rheumatology	0 (0%)	1 (1%)	1 (1%)
Surgery	3 (8%)	8 (7%)	11 (7%)
Trauma	2 (5%)	7 (6%)	9 (6%)
**Intervention type**			
Drug	4 (11%)	9 (7%)	13 (8%)
Surgery/procedure	6 (16%)	13 (11%)	19 (12%)
Counselling/lifestyle/physiotherapy	22 (59%)	72 (59%)	94 (59%)
Equipment	4 (11%)	5 (4%)	9 (6%)
Other	1 (3%)	24 (20%)	25 (16%)
**Sample size target ***			
Mean (SD)	72.8 (62.5)	258.5 (1215.7)	217.3 (1074.9)
Median (IQR)	60 (32-90)	60 (40-100)	60 (40-100)
Min-Max	6-300	20-12000	6-12000
**Cluster randomised pilot studies**	**(n=3)**	**(n=18)**	**(n=21)**
Mean number of clusters	7.3 (2.3)	9.7 (11.6)	9.3 (10.7)
Median (IQR)	6 (6-10)	6 (3-10)	6 (4-10)
Min-Max	6-10	2-45	2-45
**Number of arms**			
2	32 (86%)	111 (90%)	143 (89%)
>2	5 (14%)	12 (10%)	17 (11%)
**Number of centres**			
Single centre	19 (51%)	36 (29%)	55 (34%)
Multi-centre	18 (49%)	84 (68%)	102 (64%)
Unclear	0 (0%)	3 (2%)	3 (2%)
**Feasibility objective/s explicitly described as primary**			
Yes	9 (24%)	62 (50%)	71 (44%)
No	28 (76%)	61 (50%)	89 (56%)
**Trial outcomes address trial objectives**			
Yes	18 (49%)	91 (74%)	109 (68%)
No	0 (0%)	1 (1%)	1 (1%)
Somewhat**	19 (51%)	31 (25%)	50 (31%)
**Completely defined prespecified assessments or measurements stated**			
Yes	27 (73%)	113 (92%)	140 (88%)
Not for every outcome	10 (27%)	10 (8%)	20 (13%)
**Hypothesis testing**			
Yes	2 (5%)	16 (13%)	18 (11%)
Yes, exploratory/caution advised	18 (49%)	43 (35%)	61 (38%)
No	17 (46%)	64 (52%)	81 (51%)
**Number of uncertainties reported**			

For peer review only - http://bmjopen.bmj.com/site/about/guidelines.xhtml

One	0 (0%)	1 (1%)	1 (1%)
Multiple	37 (100%)	122 (99%)	159 (99%)
**Where uncertainties are first reported (excluding abstract)**			
Introduction	0 (0%)	1 (1%)	1 (1%)
Research question(s)	2 (5%)	4 (3%)	6 (4%)
Aim(s)	5 (14%)	16 (13%)	21 (13%)
Objective(s)	10 (27%)	62 (50%)	72 (45%)
Outcome(s)	9 (24%)	17 (14%)	26 (16%)
Outcome measure(s)	7 (19%)	16 (13%)	23 (14%)
Analysis	0 (0%)	2 (2%)	2 (1%)
Within the text under a feasibility/uncertainty heading	2 (5%)	1 (1%)	3 (2%)
Throughout the text, not in one specific area	2 (5%)	4 (3%)	6 (4%)

*Where publications reported a sample size target range (e.g. 12-16 participants), the lower bound of the target is included. A sample size target was not reported in two publications (both reporting completed pilot trials and including the actual number of recruited participants).

**Trial objective was vague (e.g., to “assess feasibility”) and the specific areas of feasibility were not explicitly stated.

For peer review only

For peer review only - http://bmjopen.bmj.com/site/about/guidelines.xhtml

Table 2 Progression criteria characteristics reported in external randomised pilot trial publications

	Completed (n=37) n (%)	Protocol (n=123) n (%)	Total (n=160) n (%)
**Feasibility outcomes informing progression criteria**			
All	14 (38%)	39 (32%)	53 (33%)
Some	22 (59%)	77 (63%)	99 (62%)
None	1 (3%)	2 (2%)	3 (2%)
Unclear*	0 (0%)	5 (4%)	5 (3%)
**Reported process for establishing progression criteria**			
**Who decided on progression criteria**			
Reported	4 (11%)	8 (6%)	12 (8%)
Not reported	33 (89%)	115 (94%)	148 (93%)
**Rationale for progression criteria**			
Reported for all progression criteria	8 (22%)	20 (16%)	28 (18%)
Reported for some criteria only	4 (11%)	12 (10%)	16 (10%)
Not reported	25 (68%)	91 (74%)	116 (73%)
**Progression criteria format**			
**Research method informing progression criteria**			
Quantitative	32 (86%)	93 (76%)	125 (78%)
Quantitative and qualitative (mixed methods)	5 (14%)	29 (24%)	34 (21%)
Unclear	0 (0%)	1 (1%)	1 (1%)
**Qualitative research contribution**			
Informs progression criteria	5 (14%)	29 (24%)	34 (21%)
Does not inform progression criteria	14 (38%)	60 (49%)	74 (46%)
Qualitative research methodology not used	18 (49%)	34 (28%)	52 (33%)
**Quantitative progression criteria target format**			
Distinct threshold	34 (92%)	99 (80%)	133 (83%)
Traffic light system	2 (5%)	18 (15%)	20 (13%)
Other	1 (3%)	6 (5%)	7 (4%)
**Reported process for assessing progression criteria to inform the progression decision**			
**Process for progression decision making**			
Reported	16 (43%)	58 (47%)	74 (46%)
Not reported	21 (57%)	65 (53%)	86 (54%)
**Who is involved in assessing progression criteria**			
Reported	5 (14%)	30 (24%)	35 (22%)
Not reported	32 (86%)	93 (76%)	125 (78%)
**Peer reviewers' reports**			
**Progression criteria mentioned in peer reviewers report**			
Yes	19 (51%)	67 (54%)	86 (54%)
Peer review comment theme			
Progression criteria were not specified	*6 (32%)*	*29 (44%)*	*35 (41%)*
Unclear whether progression criteria were specified	*1 (5%)*	*4 (6%)*	*5 (6%)*
Progression criteria rationale or justification query	*5 (26%)*	*15 (22%)*	*20 (23%)*
Other	*7 (37%)*	*19 (29%)*	*26 (30%)*
No	15 (41%)	52 (42%)	67 (42%)
Peer reviewers report unavailable	3 (8%)	4 (3%)	7 (4%)

**Feasibility uncertainties are not completely defined in the objectives and outcomes; key methodological uncertainties have been identified from those stipulated in the progression criteria*

For peer review only - http://bmjopen.bmj.com/site/about/guidelines.xhtml

Table 3 Intensions reported in completed external randomised pilot trial results publications

Data	Completed (n=37) n (%)
**Progression criteria met**	
All	17 (46%)
None	4 (11%)
Some	13 (35%)
Unclear	3 (8%)
**Progression decision**	
Proceed/future RCT is feasible	30 (81%)
With intended design	*0 (0%)*
With amendments	*28 (93%)*
Not reported whether changes will be made to definitive RCT design	*2 (7%)*
Funding intentions	
Funding for definitive RCT identified	*4 (13%)*
Non-industry	*3 (75%)*
Unclear	*1 (25%)*
Expected funding for definitive RCT not reported	*26 (87%)*
Timing intentions	
Time frame of expected progression reported	*1 (3%)*
Time frame for expected progression not reported	*29 (97%)*
Conduct further pilot/feasibility work	4 (11%)
Not proceed/future RCT is not feasible	3 (8%)
**Justification reported for the progression decision reported**	
Yes	36 (97%)
No	1 (3%)
**Comment on data quality (e.g. proportion of missing/incomplete data from questionnaires or results)**	
Yes	27 (73%)
No	10 (27%)
**Comment on refinement of hypotheses**	
Yes	1 (3%)
No	36 (97%)
**Published protocol available**	
Yes	16 (43%)
No	1 (3%)
Alternative available e.g. trial registration or REC submission	20 (54%)
**Progression criteria in earlier trial record (protocol or registration)**	
No change	10 (28%)
Yes changed	26 (72%)
Reasons for change reported	*1 (4%)*
No reason for change reported	*3 (12%)*
Progression criteria were not reported in the earlier trial record	*22 (85%)*

For peer review only

For peer review only - http://bmjopen.bmj.com/site/about/guidelines.xhtml

REFERENCES

1 Eldridge SM, Lancaster GA, Campbell MJ, *et al.* Defining Feasibility and Pilot Studies in Preparation for Randomised Controlled Trials: Development of a Conceptual Framework. *PLoS One* 2016;**11**:e0150205. doi:10.1371/journal.pone.0150205

2 Avery KNL, Williamson PR, Gamble C, *et al.* Informing efficient randomised controlled trials: Exploration of challenges in developing progression criteria for internal pilot studies. *BMJ Open* 2017;**7**. doi:10.1136/bmjopen-2016-013537

3 Mbuagbaw L, Kosa SD, Lawson DO, *et al.* The reporting of progression criteria in protocols of pilot trials designed to assess the feasibility of main trials is insufficient: a meta-epidemiological study. *Pilot feasibility Stud* 2019;**5**:120. doi:10.1186/s40814-019-0500-z

4 Eldridge SM, Chan CL, Campbell MJ, *et al.* CONSORT 2010 statement: Extension to randomised pilot and feasibility trials. *BMJ* 2016;**355**:i5239. doi:10.1136/bmj.i5239

5 Thabane L, Ma J, Chu R, *et al.* A tutorial on pilot studies: the what, why and how. *BMC Med Res Methodol* 2010;**10**:1. doi:10.1186/1471-2288-10-1

6 Mellor K, Hopewell S. An investigation of the current use of progression criteria in external randomised pilot studies: a systematic review protocol. OSF. 2020. doi:10.17605/OSF.IO/BN35K

7 Moher D, Liberati A, Tetzlaff J, *et al.* Preferred Reporting Items for Systematic Reviews and Meta-Analyses: The PRISMA Statement. *PLoS Med* 2009;**6**:e1000097. doi:10.1371/journal.pmed.1000097

8 Schulz KF, Altman DG, Moher D. CONSORT 2010 Statement: Updated guidelines for reporting parallel group randomised trials. *BMJ* 2010;**340**:698–702. doi:10.1136/bmj.c332

9 Stata Statistical Software: Release 15. StataCorp. 2017.

10 Bugge C, Williams B, Hagen S, *et al.* A process for Decision-making after Pilot and feasibility Trials (ADePT): Development following a feasibility study of a complex intervention for pelvic organ prolapse. *Trials* 2013;**14**:1. doi:10.1186/1745-6215-14-353

11 Ahnfeldt AM, Hyldig N, Li Y, *et al.* FortiColos - A multicentre study using bovine colostrum as a fortifier to human milk in very preterm infants: Study protocol for a randomised controlled pilot trial. *Trials* 2019;**20**:279. doi:10.1186/s13063-019-3367-7

12 Perepletchikova F, Kazdin AE. Treatment integrity and therapeutic change: Issues and research recommendations. *Clin Psychol Sci Pract* 2005;**12**:365–83.

13 Sidani S, Braden CJ. *Design, evaluation, and translation of nursing interventions*. John Wiley & Sons 2011.

14 Tickle-degnen L. Nuts and bolts of conducting feasibility studies. *Am J Occup Ther* 2013;**67**:1–7. doi:10.5014/ajot.2013.006270

15 Hertzog MA. Considerations in Determining Sample Size for Pilot Studies. *Wiley Period Inc Res Nurs Heal* 2008;:180–191. doi:10.1002/nur.20247

16 Lee EC, Whitehead AL, Jacques RM, *et al.* The statistical interpretation of pilot trials: Should significance thresholds be reconsidered? *BMC Med Res Methodol* 2014;**14**. doi:10.1186/1471-2288-14-41

17 Julious S, Tan SB, Machin D. *An Introduction to Statistics in Early Phase Trials - Steven Julious,*

For peer review only - http://bmjopen.bmj.com/site/about/guidelines.xhtml

Say Beng Tan, David Machin - Google Books. Wiley-Blackwell 2010.

18 Eldridge SM, Costelloe CE, Kahan BC, *et al.* How big should the pilot study for my cluster randomised trial be? *Stat Methods Med Res* 2016;**25**:1039–56. doi:10.1177/0962280215588242

19 Altman DG, Bland JM. Measurement in Medicine: The Analysis of Method Comparison Studies. *J R Stat Soc Ser D (The Stat* 1983;**32**:307–17. doi:10.2307/2987937

20 Baltussen R, Niessen L. Priority setting of health interventions: The need for multi-criteria decision analysis. *Cost Eff Resour Alloc* 2006;**4**:14. doi:10.1186/1478-7547-4-14

21 Polit DF, Beck CT. *Nursing research: Principles and methods*. Lippincott Williams & Wilkins 2004.

22 Herbert E, Julious SA, Goodacre S. Progression criteria in trials with an internal pilot: an audit of publicly funded randomised controlled trials. *Trials* 2019;**20**:1–9. doi:10.1186/s13063-019-3578-y

23 Rosala-Hallas A, Gamble C, Blazeby J, *et al.* A review of current practice in the design and assessment of internal pilots in UK NIHR clinical trials. *Trials* 2019;**20**:1–5. doi:10.1186/s13063-019-3669-9

24 Hallingberg B, Turley R, Segrott J, *et al.* Exploratory studies to decide whether and how to proceed with full-scale evaluations of public health interventions: a systematic review of guidance. *Pilot Feasibility Stud* 2018;**4**:104. doi:10.1186/s40814-018-0290-8

25 Fairhurst K, Blazeby JM, Potter S, *et al.* Value of surgical pilot and feasibility study protocols. *Br J Surg* 2019;**106**:968–78. doi:10.1002/bjs.11167

26 Walters SJ, Dos Anjos Henriques-Cadby IB, Bortolami O, *et al.* Recruitment and retention of participants in randomised controlled trials: A review of trials funded and published by the United Kingdom Health Technology Assessment Programme. *BMJ Open* 2017;**7**:e015276. doi:10.1136/bmjopen-2016-015276

27 NIHR. Guidance on applying for feasibility studies. v1.0 Jul 2017. 2019.https://www.nihr.ac.uk/documents/nihr-research-for-patient-benefit-rfpb-programme-guidance-on-applying-for-feasibility-studies/20474 (accessed 13 Nov 2019).

For peer review only

For peer review only - http://bmjopen.bmj.com/site/about/guidelines.xhtml

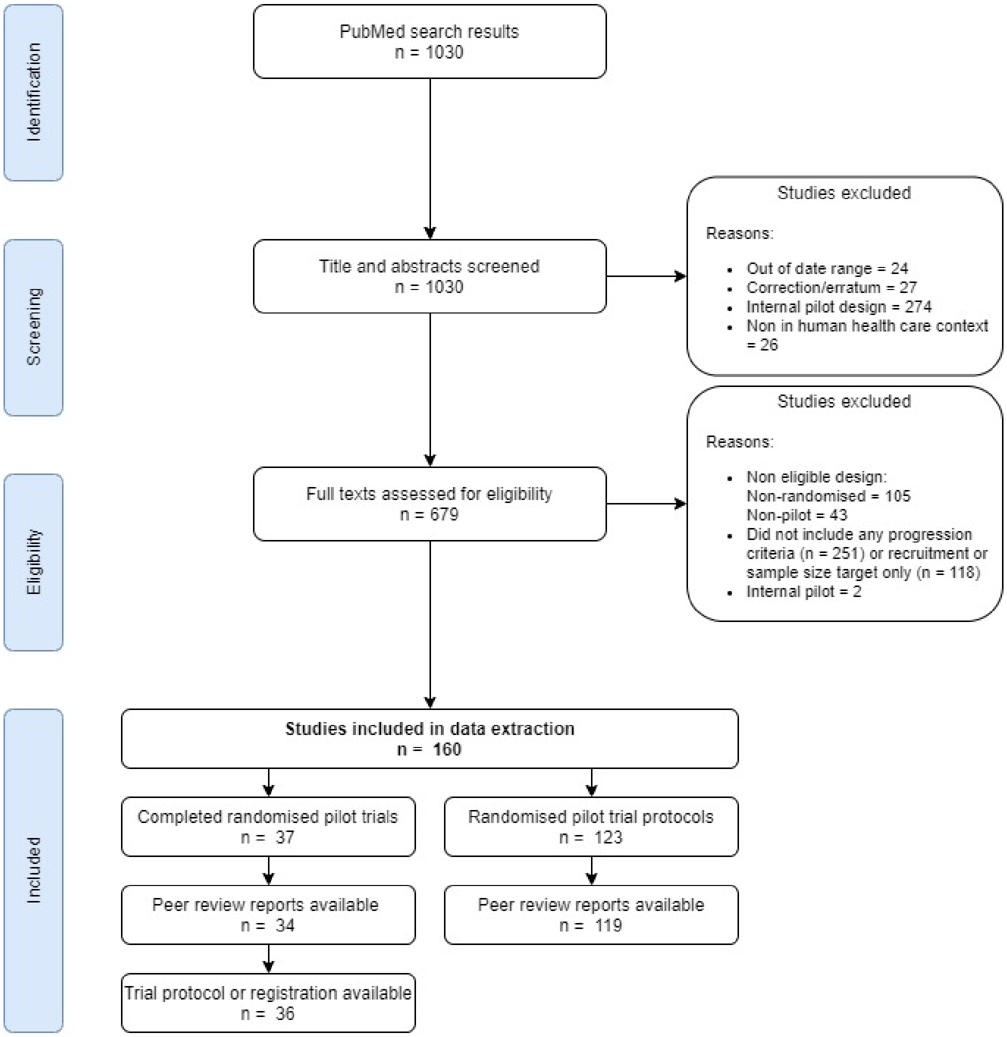

Figure 1 PRISMA flow chart

240x247mm (72 x 72 DPI)

SUPPLEMENTARY FILE 1: SEARCH STRATEGY

[PubMed search 06 Jan 2020]

No	Search terms	Results
1	**Pilot[Title]**	68704
2	**Feasibility[Title]**	31478
3	1 OR 2	98358
4	**Trial[Title/Abstract]**	576760
5	**Study[Title/Abstract]**	7478840
6	**Protocol[Title/Abstract]**	300466
7	4 OR 5 OR 6	7872797
8	**(((("Pilot and feasibility studies"[Journal])) OR "Trials"[Journal]) OR "BMJ open"[Journal]) OR "PloS one"[Journal]**	245189
9	3 AND 7 AND 8	2529
10	**9 AND 2018.01.01"[Date - Publication] : "2019.12.31"[Date - Publication]**	1030

For peer review only

SUPPLEMENTARY FILE 2: LIST OF INCLUDED STUDIES

First author	Year	Journal	Publication title
Ali	2018	*BMJ Open*	Individual cognitive stimulation therapy for people with intellectual disability and dementia: Protocol of a feasibility randomised controlled trial
Battle	2019	*BMJ Open*	A multicentre randomised feasibility STUdy evaluating the impact of a prognostic model for Management of BLunt chest wall trauma patients: STUMBL Trial
Collings	2019	*BMJ Open*	INSoles to Ease Pressure (INSTEP) Study: A multicentre, randomised controlled feasibility study to compare the effectiveness of a novel instant optimised insole with a standard insole for people with diabetic neuropathy: A study protocol
Dean	2018	*BMJ Open*	Community-based rehabilitation training after stroke: Results of a pilot randomised controlled trial (ReTrain) investigating acceptability and feasibility
Dinneen	2019	*BMJ Open*	NeuroSAFE robot-assisted laparoscopic prostatectomy versus standard robot-assisted laparoscopic prostatectomy for men with localised prostate cancer (NeuroSAFE PROOF): Protocol for a randomised controlled feasibility study
Edwards	2019	*BMJ Open*	Novel ACT-based eHealth psychoeducational intervention for students with mental distress: A study protocol for a mixed-methodology pilot trial
Froghi	2019	*BMJ Open*	Ward-based Goal-Directed Fluid Therapy (GDFT) in Acute Pancreatitis (GAP) trial: Study protocol for a feasibility randomised controlled trial
Furlano	2019	*BMJ Open*	Feasibility of a 6-month pilot randomised controlled trial of resistance training on cognition and brain health in Canadian older adults at-risk for diabetes: Study protocol
Geraghty	2018	*BMJ Open*	Using an internet intervention to support self-management of low back pain in primary care: Findings from a randomised controlled feasibility trial (SupportBack)
Griffin	2019	*BMJ Open*	Healthy Dads, Healthy Kids UK, a weight management programme for fathers: Feasibility RCT
Guagliano	2019	*BMJ Open*	Whole family-based physical activity promotion intervention: The Families Reporting Every Step to Health pilot randomised controlled trial protocol
Harper	2018	*BMJ Open*	Treatment of fatigue with physical activity and behavioural change support in vasculitis: Study protocol for an open-label randomised controlled feasibility study
Hawley-Hague	2019	*BMJ Open*	Can smartphone technology be used to support an effective home exercise intervention to prevent falls amongst community dwelling older adults?: The TOGETHER feasibility RCT study protocol

For peer review only

Hughes	2018	*BMJ Open*	Prediabetes in pregnancy, can early intervention improve outcomes? A feasibility study for a parallel randomised clinical trial
Jolly	2018	*BMJ Open*	Protocol for a feasibility trial for improving breast feeding initiation and continuation: Assets-based infant feeding help before and after birth (ABA)
Jones	2019	*BMJ Open*	Walk, Talk and Listen: A pilot randomised controlled trial targeting functional fitness and loneliness in older adults with hearing loss
Keene	2019	*BMJ Open*	Progressive functional exercise versus best practice advice for adults aged 50 years or over after ankle fracture: Protocol for a pilot randomised controlled trial in the UK - The Ankle Fracture Treatment: Enhancing Rehabilitation (AFTER) study
Lewis	2019	*BMJ Open*	Cuff Leak Test and Airway Obstruction in Mechanically Ventilated ICU Patients (COMIC): A pilot randomised controlled trial protocol
Limond	2019	*BMJ Open*	Clinical and cost-effectiveness of teen online problem-solving for adolescents who have survived an acquired brain injury in the UK: Protocol for a randomised, controlled feasibility study (TOPS-UK)
Lockstone	2019	*BMJ Open*	Non-Invasive Positive airway Pressure thErapy to Reduce Postoperative Lung complications following Upper abdominal Surgery (NIPPER PLUS): protocol for a single-centre, pilot, randomised controlled trial
Lorenzini	2019	*BMJ Open*	Measuring changes in device use of a head-mounted low vision aid after personalised telerehabilitation: Protocol for a feasibility study
McIntyre	2018	*BMJ Open*	FLUID trial: A protocol for a hospital-wide open-label cluster crossover pragmatic comparative effectiveness randomised pilot trial
Mcpherson	2019	*BMJ Open*	Children and teens in charge of their health (catch): A protocol for a feasibility randomised controlled trial of solution-focused coaching to foster healthy lifestyles in childhood disability
Morris	2019	*BMJ Open*	Dietary Approaches to the Management of type 2 Diabetes (DIAMOND): Protocol for a randomised feasibility trial
Munce	2019	*BMJ Open*	Ontario Brain Injury Association Peer Support Program: A mixed methods protocol for a pilot randomised controlled trial
Neves	2019	*BMJ Open*	Protocol for a feasibility study of a cohort embedded randomised controlled trial comparing NEphron Sparing Treatment (NEST) for small renal masses
O'Connor	2019	*BMJ Open*	SAFETEL randomised controlled feasibility trial of a safety planning intervention with follow-up telephone contact to reduce suicidal behaviour: Study protocol
Orkin	2019	*BMJ Open*	Protocol for a mixed-methods feasibility study for the surviving opioid overdose with naloxone education and resuscitation (SOONER) randomised control trial

For peer review only

Pai	2019	*BMJ Open*	Protocol for a double-blind, randomised, placebo-controlled pilot study for assessing the feasibility and efficacy of faecal microbiota transplant in a paediatric Crohn's disease population: PediCRaFT Trial
Papathanassoglou	2019	*BMJ Open*	Relaxation for Critically ill Patient Outcomes and S tress-coping Enhancement (REPOSE): A protocol for a pilot randomised trial of an integrative intervention to improve critically ill patients' delirium and related outcomes
Pennington	2019	*BMJ Open*	Internet delivery of intensive speech and language therapy for children with cerebral palsy: A pilot randomised controlled trial
Pouw	2018	*BMJ Open*	Hospital at Home care for older patients with cognitive impairment: A protocol for a randomised controlled feasibility trial
Quraishi	2019	*BMJ Open*	STOP-Colitis pilot trial protocol: A prospective, open-label, randomised pilot study to assess two possible routes of faecal microbiota transplant delivery in patients with ulcerative colitis
Reddington	2018	*BMJ Open*	Does early intervention improve outcomes in the physiotherapy management of lumbar radicular syndrome? Results of the POLAR pilot randomised controlled trial
Ribeiro	2019	*BMJ Open*	Effectiveness of a tailored rehabilitation versus standard strengthening programme for patients with shoulder pain: A protocol for a feasibility randomised controlled trial (the Otago MASTER trial)
Schults	2018	*BMJ Open*	Normal saline instillation versus no normal saline instillation and lung Recruitment versus no lung recruitment with paediatric Endotracheal Suction: The NARES trial. A study protocol for a pilot, factorial randomised controlled trial
Sharma	2018	*BMJ Open*	Pain education for patients with non-specific low back pain in Nepal: Protocol of a feasibility randomised clinical trial (PEN-LBP Trial)
Sharma	2019	*BMJ Open*	Results of a feasibility randomised clinical trial on pain education for low back pain in Nepal: The Pain Education in Nepal-Low Back Pain (PEN-LBP) feasibility trial
Steare	2019	*BMJ Open*	App to support Recovery in Early Intervention Services (ARIES) study: Protocol of a feasibility randomised controlled trial of a self-management Smartphone application for psychosis
Sugg	2018	*BMJ Open*	Morita Therapy for depression (Morita Trial): A pilot randomised controlled trial
Thyer	2018	*BMJ Open*	Randomised controlled feasibility trial of the Active Communication Education programme plus hearing aid provision versus hearing aid provision alone (ACE to HEAR): A study protocol
Wall	2018	*BMJ Open*	Safety and feasibility evaluation of tourniquets for total knee replacement (SAFE-TKR): Study protocol
Wiangkham	2019	*BMJ Open*	Pragmatic cluster randomised double-blind pilot and feasibility trial of an active behavioural physiotherapy intervention for acute non-specific neck pain: A mixed-methods protocol
Wootton	2019	*BMJ Open*	Telehealth and texting intervention to improve HIV care engagement, mental health and substance use outcomes in youth living with HIV: A pilot feasibility and acceptability study protocol

For peer review only

Yeung	2019	*BMJ Open*	Randomised controlled trial to investigate the effectiveness of thoracic epidural and paravertebral blockade in reducing chronic post-thoracotomy pain (TOPIC): A pilot study to assess feasibility of a large multicentre trial
Abokhrais	2018	*PAFS*	A pilot randomised double blind controlled trial of the efficacy of purified fatty acids for the treatment of women with endometriosis-associated pain (PurFECT): Study protocol
Artom	2019	*PAFS*	Cognitive-behavioural therapy for the management of inflammatory bowel disease-fatigue: A feasibility randomised controlled trial
Aunger	2019	*PAFS*	A novel behavioural INTErvention to REduce Sitting Time in older adults undergoing orthopaedic surgery (INTEREST): Protocol for a randomised controlled feasibility study
Bérubé	2019	*PAFS*	Feasibility of a tapering opioids prescription program for trauma patients at high risk of chronic consumption (TOPPtrauma): Protocol for a pilot randomized controlled trial
Bick	2019	*PAFS*	Protocol for a two-arm feasibility RCT to support postnatal maternal weight management and positive lifestyle behaviour in women from an ethnically diverse inner city population: The SWAN feasibility trial
Bjornstad	2019	*PAFS*	Healthy Parent Carers peer-led group-based health promotion intervention for parent carers of disabled children: Protocol for a feasibility study using a parallel group randomised controlled trial design
Blanton	2019	*PAFS*	A web-based carepartner-integrated rehabilitation program for persons with stroke: Study protocol for a pilot randomized controlled trial
Bostrøm	2019	*PAFS*	Clinical comparative effectiveness of acupuncture versus manual therapy treatment of lateral epicondylitis: Feasibility randomized clinical trial
Bourne	2019	*PAFS*	Electrically assisted cycling for individuals with type 2 diabetes mellitus: Protocol for a pilot randomized controlled trial
Bowyer-Crane	2019	*PAFS*	A randomised controlled feasibility trial and qualitative evaluation of an early years language development intervention: Study protocol of the 'outcomes of Talking Together evaluation and results' (oTTer) project
Bryant	2018	*PAFS*	Cluster randomised controlled feasibility study of HENRY: A community-based intervention aimed at reducing obesity rates in preschool children
Bui	2019	*PAFS*	App-based supplemental exercise during inpatient orthopaedic rehabilitation increases activity levels: A pilot randomised control trial
Carswell	2019	*PAFS*	Implementing an arts-based intervention for patients with end-stage kidney disease whilst receiving haemodialysis: A feasibility study protocol 11 Medical and Health Sciences 1117 Public Health and Health Services 11 Medical and Health Sciences 1103 Clini
Clark	2019	*PAFS*	Saline versus albumin fluid for extracorporeal removal with slow low efficiency dialysis (SAFER-SLED): Study protocol for a pilot trial

For peer review only

Coe	2018	*PAFS*	A protocol for a randomised double-blind placebo-controlled feasibility study to determine whether the daily consumption of flavonoid-rich pure cocoa has the potential to reduce fatigue in people with relapsing and remitting multiple sclerosis (RRMS)
Courtier	2018	*PAFS*	ACTIVE - A randomised feasibility trial study protocol of a behavioural intervention to reduce fatigue in women undergoing radiotherapy for early breast cancer: Study protocol
Cro	2018	*PAFS*	Measuring skin necrosis in a randomised controlled feasibility trial of heat preconditioning on wound healing after reconstructive breast surgery: Study protocol and statistical analysis plan for the PREHEAT trial
De Oliveira Braga	2019	*PAFS*	EMPOWER-PD - A physical therapy intervention to empower the individuals with Parkinson's disease: A study protocol for a feasibility randomized controlled trial
Deary	2018	*PAFS*	A psychosocial intervention for the management of functional dysphonia: Complex intervention development and pilot randomised trial
Ditai	2019	*PAFS*	BabyGel pilot: A pilot cluster randomised trial of the provision of alcohol handgel to postpartum mothers to prevent neonatal and young infant infection-related morbidity in the community
Downey	2018	*PAFS*	Trial of Remote Continuous versus Intermittent NEWS monitoring after major surgery (TRaCINg): Protocol for a feasibility randomised controlled trial
Drew	2019	*PAFS*	A protocol for a randomised controlled, double-blind feasibility trial investigating fluoxetine treatment in improving memory and learning impairments in patients with mesial temporal lobe epilepsy: Fluoxetine, Learning and Memory in Epilepsy (FLAME trial
Duncan	2018	*PAFS*	Physical therapy and deep brain stimulation in Parkinson's Disease: Protocol for a pilot randomized controlled trial
Dunn	2019	*PAFS*	Evaluating Augmented Depression Therapy (ADepT): Study protocol for a pilot randomised controlled trial
Fuller	2018	*PAFS*	The ACUTE (Ambulance CPAP: Use, Treatment effect and economics) feasibility study: A pilot randomised controlled trial of prehospital CPAP for acute respiratory failure
Golla	2018	*PAFS*	Home-based balance training using Wii Fit™: A pilot randomised controlled trial with mobile older stroke survivors
Hayes	2019	*PAFS*	We Can Quit2 (WCQ2): A community-based intervention on smoking cessation for women living in disadvantaged areas of Ireland - Study protocol for a pilot cluster randomised controlled trial
Hilari	2019	*PAFS*	Adjustment with aphasia after stroke: Study protocol for a pilot feasibility randomised controlled trial for SUpporting wellbeing through PEeR Befriending (SUPERB)
Horne	2019	*PAFS*	Regaining Confidence after Stroke (RCAS): A feasibility randomised controlled trial (RCT)
Jones	2019	*PAFS*	Rapid Analgesia for Prehospital hip Disruption (RAPID): Findings from a randomised feasibility study

For peer review only

Kebbe	2019	*PAFS*	Feasibility, user experiences, and preliminary effect of Conversation Cards for Adolescents© on collaborative goal-setting and behavior change: Protocol for a pilot randomized controlled trial
Kohrt	2018	*PAFS*	Reducing stigma among healthcare providers to improve mental health services (RESHAPE): Protocol for a pilot cluster randomized controlled trial of a stigma reduction intervention for training primary healthcare workers in Nepal
Lodder	2019	*PAFS*	Stigma of living as an autism carer: A brief psycho-social support intervention (SOLACE). Study protocol for a randomised controlled feasibility study
Logan	2018	*PAFS*	Standing Practice In Rehabilitation Early after Stroke (SPIRES): A functional standing frame programme (prolonged standing and repeated sit to stand) to improve function and quality of life and reduce neuromuscular impairment in people with severe sub-acute stroke-a protocol for a feasibility randomised controlled trial
Loughnan	2019	*PAFS*	A single-centre, randomised controlled feasibility pilot trial comparing performance of direct laryngoscopy versus videolaryngoscopy for endotracheal intubation in surgical patients
Malden	2019	*PAFS*	A feasibility cluster randomised controlled trial of a preschool obesity prevention intervention: ToyBox-Scotland
McGovern	2018	*PAFS*	Promoting Alcohol Reduction in Non- Treatment Seeking parents (PAReNTS): A protocol for a pilot feasibility cluster randomised controlled trial of alcohol screening and brief interventions to reduce parental alcohol use disorders in vulnerable families
McIntosh	2018	*PAFS*	On the Road to Recovery psychological therapy versus treatment as usual for forensic mental health patients: Study protocol for a randomized controlled feasibility trial
Mehta	2019	*PAFS*	A randomised controlled feasibility trial to evaluate local heat preconditioning on wound healing after reconstructive breast surgery: The preHEAT trial
Meiksin	2019	*PAFS*	Protocol for pilot cluster RCT of project respect: A school-based intervention to prevent dating and relationship violence and address health inequalities among young people
Milbury	2018	*PAFS*	A research protocol for a pilot randomized controlled trial designed to examine the feasibility of a couple-based mind-body intervention for patients with metastatic lung cancer and their partners
Milbury	2019	*PAFS*	A research protocol for a pilot, randomized controlled trial designed to examine the feasibility of a dyadic versus individual yoga program for family caregivers of glioma patients undergoing radiotherapy
Moore	2018	*PAFS*	Prehospital recognition and antibiotics for 999 patients with sepsis: Protocol for a feasibility study
Morgan	2019	*PAFS*	A pilot randomised controlled trial of physical activity facilitation for older adults: Feasibility study findings
Morton	2018	*PAFS*	Chlorhexidine vaginal preparation versus standard treatment at caesarean section to reduce endometritis and prevent sepsis - A feasibility study protocol (the PREPS trial)

For peer review only

Murphy	2018	*PAFS*	Supporting general practitioner-based care for poorly controlled type 2 diabetes mellitus (the DECIDE study): Feasibility study and protocol for a pilot cluster randomised controlled trial
Mutedzi	2019	*PAFS*	Improving bereavement outcomes in Zimbabwe: Protocol for a feasibility cluster trial of the 9-cell bereavement tool
Myers	2019	*PAFS*	Accelerometer-based assessment of physical activity within the Fun for Wellness online behavioral intervention: Protocol for a feasibility study
Negm	2018	*PAFS*	Getting fit for hip and knee replacement: A protocol for the Fit-Joints pilot randomized controlled trial of a multi-modal intervention in frail patients with osteoarthritis
Newlands	2019	*PAFS*	Pilot randomised controlled trial of Weight Watchers® referral with or without dietitianled group support for weight loss in women treated for breast cancer: The BRIGHT (BReast cancer weIGHT loss) trial
O'Regan	2019	*PAFS*	An evaluation of an intervention designed to help inactive adults become more active with a peer mentoring component: A protocol for a cluster randomised feasibility trial of the Move for Life programme
Paul	2019	*PAFS*	Vital sign monitoring with continuous pulse oximetry and wireless clinical notification after surgery (the VIGILANCE pilot study)- A randomized controlled pilot trial
Payne	2018	*PAFS*	Study protocol for a randomised pilot study of a computer-based, non-pharmacological cognitive intervention for motor slowing and motor fatigue in Parkinson's disease
Perman-Howe	2018	*PAFS*	The effect of alcohol strength on alcohol consumption: A randomised controlled cross-over pilot trial
Philip	2019	*PAFS*	A randomised phase II trial to examine feasibility of standardised, early palliative (STEP) care for patients with advanced cancer and their families [ACTRN12617000534381]: A research protocol
Pile	2018	*PAFS*	A brief early intervention for adolescent depression that targets emotional mental images and memories: Protocol for a feasibility randomised controlled trial (IMAGINE trial)
Ponsford	2018	*PAFS*	Study protocol for the optimisation, feasibility testing and pilot cluster randomised trial of Positive Choices: A school-based social marketing intervention to promote sexual health, prevent unintended teenage pregnancies and address health inequalities
Purcell	2018	*PAFS*	Eutectic mixture of local anaesthetics (EMLA®) as a primary dressing on painful chronic leg ulcers: A pilot randomised controlled trial
Qurashi	2019	*PAFS*	Glycopyrrolate in comparison to hyoscine hydrobromide and placebo in the treatment of hypersalivation induced by clozapine (GOTHIC1): A feasibility study
Rowe	2019	*PAFS*	A classroom-based intervention targeting working memory, attention and language skills in 4-5 year olds (RECALL): Study protocol for a cluster randomised feasibility trial

For peer review only

Sanfilippo	2019	*PAFS*	A study protocol for testing the feasibility of a randomised stepped wedge cluster design to investigate a Community Health Intervention through Musical Engagement (CHIME) for perinatal mental health in the Gambia
Sangraula	2018	*PAFS*	Protocol for a feasibility study of group-based focused psychosocial support to improve the psychosocial well-being and functioning of adults affected by humanitarian crises in Nepal: Group Problem Management plus (PM+)
Schlaeger	2018	*PAFS*	Double-blind acupuncture needles: A multi-needle, multi-session randomized feasibility study
Schmitz	2019	*PAFS*	Impact of endurance exercise and probiotic supplementation on the intestinal microbiota: A cross-over pilot study
Shvedko	2018	*PAFS*	Physical Activity Intervention for Loneliness (PAIL) in community-dwelling older adults: Protocol for a feasibility study
Slobogean	2019	*PAFS*	Fixation using alternative implants for the treatment of hip fractures (FAITH-2): Design and rationale for a pilot multi-centre 2 × 2 factorial randomized controlled trial in young femoral neck fracture patients
Snowden	2018	*PAFS*	Preoperative Behavioural Intervention versus standard care to Reduce Drinking before elective orthopaedic Surgery (PRE-OP BIRDS): Protocol for a multicentre pilot randomised controlled trial
Sosnowski	2018	*PAFS*	A feasibility study of a randomised controlled trial to examine the impact of the ABCDE bundle on quality of life in ICU survivors
Tan	2019	*PAFS*	The efficacy of foot orthoses in individuals with patellofemoral osteoarthritis: A randomised feasibility trial
Timko	2018	*PAFS*	Cognitive remediation therapy (CRT) as a pretreatment intervention for adolescents with anorexia nervosa during medical hospitalization: A pilot randomized controlled trial protocol
Totty	2019	*PAFS*	Assessing the effectiveness of dialkylcarbamoylchloride (DACC)-coated post-operative dressings versus standard care in the prevention of surgical site infection in clean or clean-contaminated, vascular surgery (the DRESSINg trial): Study protocol for a pilot feasibility randomised controlled trial
Volkmer	2018	*PAFS*	The 'Better Conversations with Primary Progressive Aphasia (BCPPA)' program for people with PPA (Primary Progressive Aphasia): Protocol for a randomised controlled pilot study
Vranceanu	2019	*PAFS*	Results of a feasibility randomized controlled trial (RCT) of the Toolkit for Optimal Recovery (TOR): A live video program to prevent chronic pain in at-risk adults with orthopedic injuries
Whitehead	2019	*PAFS*	HATRIC: A study of Pelargonium sidoides root extract EPs®7630 (Kaloba®) for the treatment of acute cough due to lower respiratory tract infection in adults-study protocol for a double blind, placebocontrolled randomised feasibility trial
Wiggins	2018	*PAFS*	Testing the effectiveness of REACH Pregnancy Circles group antenatal care: Protocol for a randomised controlled pilot trial

For peer review only

Wong	2018	*PAFS*	Thiamine versus placebo in older heart failure patients: Study protocol for a randomized controlled crossover feasibility trial (THIAMINE-HF)
Wurz	2019	*PAFS*	Exploring the feasibility and acceptability of a mixed-methods pilot randomized controlled trial testing a 12-week physical activity intervention with adolescent and young adult cancer survivors
Hilton	2018	*PLoS ONE*	Randomised feasibility trial to compare three standard of care chemotherapy regimens for early stage triple-negative breast cancer (REaCT-TNBC trial)
Karlsson	2019	*PLoS ONE*	Feasibility of preoperative supervised home-based exercise in older adults undergoing colorectal cancer surgery – A randomized controlled design
Wiangkham	2019	*PLoS ONE*	A cluster randomised, double-blind pilot and feasibility trial of an active behavioural physiotherapy intervention for acute whiplash-associated disorder (WAD)II
Ahnfeldt	2019	*Trials*	FortiColos - A multicentre study using bovine colostrum as a fortifier to human milk in very preterm infants: Study protocol for a randomised controlled pilot trial
Barrett	2018	*Trials*	Feasibility of a physical activity programme embedded into the daily lives of older adults living in nursing homes: Protocol for a randomised controlled pilot feasibility study
Brennan	2018	*Trials*	Prevention of striae gravidarum: Study protocol for a pilot randomised controlled trial
Browne	2019	*Trials*	Probiotics in pregnancy: Protocol of a double-blind randomized controlled pilot trial for pregnant women with depression and anxiety (PIP pilot trial)
Burroughs	2018	*Trials*	A feasibility study for NOn-Traditional providers to support the management of Elderly People with Anxiety and Depression: The NOTEPAD study Protocol
Cao	2018	*Trials*	Aerobic exercise-based cardiac rehabilitation in Chinese patients with coronary heart disease: Study protocol for a pilot randomized controlled trial
Chhetri	2019	*Trials*	Repetitive vascular occlusion stimulus (RVOS) versus standard care to prevent muscle wasting in critically ill patients (ROSProx):a study protocol for a pilot randomised controlled trial
Crawford	2018	*Trials*	Psychological Support for Personality (PSP) versus treatment as usual: Study protocol for a feasibility randomized controlled trial of a low intensity intervention for people with personality disorder
Deb	2018	*Trials*	Aggression Following Traumatic brain injury: Effectiveness of Risperidone (AFTER): Study protocol for a feasibility randomised controlled trial
Forster	2018	*Trials*	An intervention to support stroke survivors and their carers in the longer term (LoTS2Care): Study protocol for a cluster randomised controlled feasibility trial

For peer review only

Froghi	2018	*Trials*	Cardiac output Optimisation following Liver Transplant (COLT) trial: Study protocol for a feasibility randomised controlled trial
Greenwood	2018	*Trials*	The U&I study: Study protocol for a feasibility randomised controlled trial of a pre-cognitive behavioural therapy digital 'informed choice' intervention to improve attitudes towards uptake and implementation of CBT for psychosis
He	2018	*Trials*	Xue-Fu-Zhu-Yu capsule in the treatment of qi stagnation and blood stasis syndrome: a study protocol for a randomised controlled pilot and feasibility trial
Hutchings	2018	*Trials*	CONTRACT Study - CONservative TReatment of Appendicitis in Children (feasibility): Study protocol for a randomised controlled Trial
Lee	2018	*Trials*	Effect and safety of acupuncture for Hwa-byung, an anger syndrome: A study protocol of a randomized controlled pilot trial
Linnemayr	2018	*Trials*	Behavioral economics-based incentives supported by mobile technology on HIV knowledge and testing frequency among Latino/a men who have sex with men and transgender women: Protocol for a randomized pilot study to test intervention feasibility and acceptability
Littlewood	2019	*Trials*	Protocol for a multi-centre pilot and feasibility randomised controlled trial with a nested qualitative study: Rehabilitation following rotator cuff repair (the RaCeR study)
Macken	2018	*Trials*	Palliative long-term abdominal drains versus repeated drainage in individuals with untreatable ascites due to advanced cirrhosis: Study protocol for a feasibility randomised controlled trial
Marsh	2018	*Trials*	A novel integrated dressing to secure peripheral intravenous catheters in an adult acute hospital: A pilot randomised controlled trial
Marsh	2018	*Trials*	Expert versus generalist inserters for peripheral intravenous catheter insertion: A pilot randomised controlled trial
Mayo-Wilson	2019	*Trials*	Microenterprise intervention to reduce sexual risk behaviors and increase employment and HIV preventive practices in economically-vulnerable African-American young adults (EMERGE): Protocol for a feasibility randomized clinical trial
Nymberg	2018	*Trials*	Pilot study on increased adherence to physical activity on prescription (PAP) through mindfulness: Study protocol
Pace	2019	*Trials*	Cognitively-Based Compassion Training versus cancer health education to improve health-related quality of life in survivors of solid tumor cancers and their informal caregivers: Study protocol for a randomized controlled pilot trial
Payne Riches	2019	*Trials*	The Salt Swap intervention to reduce salt intake in people with high blood pressure: Protocol for a feasibility randomised controlled trial

For peer review only

Poolman	2019	*Trials*	CARer-ADministration of as-needed subcutaneous medication for breakthrough symptoms in homebased dying patients (CARiAD): Study protocol for a UK-based open randomised pilot trial
Pressman	2019	*Trials*	Conducting a pilot randomized controlled trial of community-based mindfulness-based stress reduction versus usual care for moderate-to-severe migraine: Protocol for the Mindfulness and Migraine Study (M&M)
Pyle	2019	*Trials*	Study protocol for a randomised controlled trial of CBT vs antipsychotics vs both in 14-18-year-olds: Managing Adolescent first episode Psychosis: A feasibility study (MAPS)
Russell	2018	*Trials*	Feasibility of an online mindfulness-based program for patients with melanoma: Study protocol for a randomised controlled trial
Selfe	2019	*Trials*	Acceptability and feasibility of a 12-week yoga vs. educational film program for the management of restless legs syndrome (RLS): Study protocol for a randomized controlled trial
Taylor	2019	*Trials*	Protocol for a randomised controlled feasibility study examining the efficacy of brief cognitive therapy for the Treatment of Anxiety Disorders in Adolescents (TAD-A)
Van Oostveen	2018	*Trials*	Prevention of Infections in Cardiac Surgery study (PICS): Study protocol for a pragmatic cluster-randomized factorial crossover pilot trial
Watt	2019	*Trials*	A counseling intervention to address HIV stigma at entry into antenatal care in Tanzania (Maisha): Study protocol for a pilot randomized controlled trial
Wright	2018	*Trials*	The clinical and cost effectiveness of adapted dialectical behaviour therapy (DBT) for bipolar mood instability in primary care (ThrIVe-B programme): A feasibility study
Youssef	2019	*Trials*	Addition of a new three-dimensional adjustable cervical thoracic orthosis to a multi-modal program in the treatment of nonspecific neck pain: Study protocol for a randomised pilot trial
Zeng	2019	*Trials*	Si-ni-tang (a Chinese herbal formula) for improving immunofunction in sepsis: Study protocol for a pilot randomized controlled trial

BMJ: *British Medical Journal*; *PAFS*: *Pilot and Feasibility Studies; PLoS*: *Public Library of Science*

For peer review only

SUPPLEMENTARY FILE 3: INDICATOR OF FEASIBILITY CONTRIBUTING TO PROGRESSION CRITERIA

Domain	Indicator of feasibility contributing to progression criteria	Number of publications
Management	Data completion or missing data	38
	Barriers or challenges to intervention implementation	7
	Develop or test training materials	4
	Protocol components work together	2
	Data analysis	2
	Data collection methods are suitable/fit for purpose	1
	Data management	1
	Time to Ethics approvals at each site	1
	Time to readiness to initiate the clinical trial	1
Process	Recruitment rates	113
	Retention or attrition rate	106
	Non(compliance) or adherence rate (participants)	66
	Intervention acceptability or evaluation (patients)	33
	Withdrawal or completion rate (trial or intervention)	22
	Intervention acceptability or evaluation (non-patients)	17
	Consent or refusal rate	16
	Randomisation acceptability or rate	15
	Intervention fidelity	15
	Understanding or acceptability of data collection tools/assessments/methods	14
	Non(compliance) or adherence rates (non-participants)	12
	Participant identification or screening	10
	Eligibility rate	9
	Trial acceptability or evaluation (patients)	8
	Characteristics or properties of trial outcome measures	7
	Randomisation adequacy	5
	Crossover or contamination between arms	4
	Enrolment rate	5
	Uptake or engagement rate	3
	Definitive study sample size is achievable	3
	Trial acceptability or evaluation (non-patients)	2
	Success or failure rate	2
	Understanding or acceptability of study instructions	2
	Blinding procedures	1
	Eligibility criteria	1
	Describe control group	2
	Recruitment process	2
	Understanding or acceptability of study information	1
	Allocation concealment	1
	Intervention credibility	1
	Intervention suitability	1
	Completeness of biological sample collection	1
	Interest in using the intervention post-study	1
	Positive expected net gain of sampling from a definitive trial	1

For peer review only

Resources	Centre or investigator recruitment, willingness or capacity	21
	Determining process time	7
	Collection of outcomes relevant to future economic evaluation	4
	Equipment or resource reliability	2
	Venue, location or setting appropriate	1
	Determining capacity	1
	Intervention agreement between methods	1
Scientific	Safety, adverse events, unintended consequences or harms	25
	Estimate of treatment effect	11
	Estimate of variance of treatment effect	6
	Patient response	5
	Signal of efficacy	4
	Estimate intracluster correlation coefficient	1
	Context and mechanisms of action	1
	Intervention tolerability	1

For peer review only

PRISMA 2009 Checklist

Section/topic	#	Checklist item	Reported on page #
**TITLE**			
Title	1	Identify the report as a systematic review, meta-analysis, or both.	1
**ABSTRACT**			
Structured summary	2	Provide a structured summary including, as applicable: background; objectives; data sources; study eligibility criteria, participants, and interventions; study appraisal and synthesis methods; results; limitations; conclusions and implications of key findings; systematic review registration number.	2
**INTRODUCTION**			
Rationale	3	Describe the rationale for the review in the context of what is already known.	4
Objectives	4	Provide an explicit statement of questions being addressed with reference to participants, interventions, comparisons, outcomes, and study design (PICOS).	4
**METHODS**			
Protocol and registration	5	Indicate if a review protocol exists, if and where it can be accessed (e.g., Web address), and, if available, provide registration information including registration number.	4
Eligibility criteria	6	Specify study characteristics (e.g., PICOS, length of follow-up) and report characteristics (e.g., years considered, language, publication status) used as criteria for eligibility, giving rationale.	4
Information sources	7	Describe all information sources (e.g., databases with dates of coverage, contact with study authors to identify additional studies) in the search and date last searched.	5
Search	8	Present full electronic search strategy for at least one database, including any limits used, such that it could be repeated.	5
Study selection	9	State the process for selecting studies (i.e., screening, eligibility, included in systematic review, and, if applicable, included in the meta-analysis).	5
Data collection process	10	Describe method of data extraction from reports (e.g., piloted forms, independently, in duplicate) and any processes for obtaining and confirming data from investigators.	5
Data items	11	List and define all variables for which data were sought (e.g., PICOS, funding sources) and any assumptions and simplifications made.	5
Risk of bias in individual studies	12	Describe methods used for assessing risk of bias of individual studies (including specification of whether this was done at the study or outcome level), and how this information is to be used in any data synthesis.	N/A
Summary measures	13	State the principal summary measures (e.g., risk ratio, difference in means).	5
Synthesis of results	14	Describe the methods of handling data and combining results of studies, if done, including measures of consistency (e.g., I^2) for each meta-analysis.	5

For peer review only - http://bmjopen.bmj.com/site/about/guidelines.xhtml

PRISMA 2009 Checklist

Section/topic	#	Checklist item	Reported on page #
Risk of bias across studies	15	Specify any assessment of risk of bias that may affect the cumulative evidence (e.g., publication bias, selective reporting within studies).	N/A
Additional analyses	16	Describe methods of additional analyses (e.g., sensitivity or subgroup analyses, meta-regression), if done, indicating which were pre-specified.	N/A
**RESULTS**			
Study selection	17	Give numbers of studies screened, assessed for eligibility, and included in the review, with reasons for exclusions at each stage, ideally with a flow diagram.	6
Study characteristics	18	For each study, present characteristics for which data were extracted (e.g., study size, PICOS, follow-up period) and provide the citations.	6
Risk of bias within studies	19	Present data on risk of bias of each study and, if available, any outcome level assessment (see item 12).	N/A
Results of individual studies	20	For all outcomes considered (benefits or harms), present, for each study: (a) simple summary data for each intervention group (b) effect estimates and confidence intervals, ideally with a forest plot.	6-10
Synthesis of results	21	Present results of each meta-analysis done, including confidence intervals and measures of consistency.	N/A
Risk of bias across studies	22	Present results of any assessment of risk of bias across studies (see Item 15).	N/A
Additional analysis	23	Give results of additional analyses, if done (e.g., sensitivity or subgroup analyses, meta-regression [see Item 16]).	N/A
**DISCUSSION**			
Summary of evidence	24	Summarize the main findings including the strength of evidence for each main outcome; consider their relevance to key groups (e.g., healthcare providers, users, and policy makers).	10
Limitations	25	Discuss limitations at study and outcome level (e.g., risk of bias), and at review-level (e.g., incomplete retrieval of identified research, reporting bias).	10
Conclusions	26	Provide a general interpretation of the results in the context of other evidence, and implications for future research.	11
**FUNDING**			
Funding	27	Describe sources of funding for the systematic review and other support (e.g., supply of data); role of funders for the systematic review.	12

From: Moher D, Liberati A, Tetzlaff J, Altman DG, The PRISMA Group (2009). Preferred Reporting Items for Systematic Reviews and Meta-Analyses: The PRISMA Statement. PLoS Med 6(7): e1000097. doi:10.1371/journal.pmed1000097

For more information, visit: **www.prisma-statement.org**.

For peer review only - http://bmjopen.bmj.com/site/about/guidelines.xhtml

BMJ Open

Progression from external pilot to definitive randomised controlled trial: A methodological review of progression criteria reporting

Journal:	*BMJ Open*
Manuscript ID	bmjopen-2020-048178.R1
Article Type:	Original research
Date Submitted by the Author:	26-May-2021
Complete List of Authors:	Mellor, Katie; University of Oxford, Centre for Statistics in Medicine, Nuffield Department of Orthopaedics, Rheumatology and Musculoskeletal Sciences<br>Eddy, Saskia; Queen Mary University of London, Institute of Population Health Sciences, Barts and the London School of Medicine and Dentistry<br>Peckham, Nicholas; University of Oxford, Centre for Statistics in Medicine, Nuffield Department of Orthopaedics, Rheumatology and Musculoskeletal Sciences<br>Bond, Christine; University of Aberdeen, Institute of Applied Health Sciences<br>Campbell, Michael; The University of Sheffield, School of Health and Related Research<br>Lancaster, Gillian; Keele University, School of Medicine & Keele Clinical Trials Unit (CTU)<br>Thabane, Lehana ; McMaster University, Department of Health Research Methods, Evidence and Impact<br>Eldridge, Sandra; Queen Mary University of London, Institute of Population Health Sciences, Barts and the London School of Medicine and Dentistry<br>Dutton, Susan; University of Oxford, Centre for Statistics in Medicine, Nuffield Department of Orthopaedics, Rheumatology and Musculoskeletal Sciences<br>Hopewell, Sally; University of Oxford, Centre for Statistics in Medicine, Nuffield Department of Orthopaedics, Rheumatology and Musculoskeletal Sciences
**Primary Subject Heading**:	Research methods
Secondary Subject Heading:	Medical publishing and peer review
Keywords:	STATISTICS & RESEARCH METHODS, Protocols & guidelines < HEALTH SERVICES ADMINISTRATION & MANAGEMENT, Clinical trials < THERAPEUTICS

SCHOLARONE™
Manuscripts

I, the Submitting Author has the right to grant and does grant on behalf of all authors of the Work (as defined in the below author licence), an exclusive licence and/or a non-exclusive licence for contributions from authors who are: i) UK Crown employees; ii) where BMJ has agreed a CC-BY licence shall apply, and/or iii) in accordance with the terms applicable for US Federal Government officers or employees acting as part of their official duties; on a worldwide, perpetual, irrevocable, royalty-free basis to BMJ Publishing Group Ltd ("BMJ") its licensees and where the relevant Journal is co-owned by BMJ to the co-owners of the Journal, to publish the Work in this journal and any other BMJ products and to exploit all rights, as set out in our licence.

The Submitting Author accepts and understands that any supply made under these terms is made by BMJ to the Submitting Author unless you are acting as an employee on behalf of your employer or a postgraduate student of an affiliated institution which is paying any applicable article publishing charge ("APC") for Open Access articles. Where the Submitting Author wishes to make the Work available on an Open Access basis (and intends to pay the relevant APC), the terms of reuse of such Open Access shall be governed by a Creative Commons licence – details of these licences and which Creative Commons licence will apply to this Work are set out in our licence referred to above.

Other than as permitted in any relevant BMJ Author's Self Archiving Policies, I confirm this Work has not been accepted for publication elsewhere, is not being considered for publication elsewhere and does not duplicate material already published. I confirm all authors consent to publication of this Work and authorise the granting of this licence.

For peer review only

BMJ Open manuscript v2.0, resubmission

Progression from external pilot to definitive randomised controlled trial: A methodological review of progression criteria reporting

Authors

Katie Mellor[1], Saskia Eddy[2], Nicholas Peckham[1], Christine M Bond[3], Michael J Campbell[4], Gillian A Lancaster[5], Lehana Thabane[6], Sandra M Eldridge[2], Susan J Dutton[1], Sally Hopewell[1]

Author information

[1]Centre for Statistics in Medicine, Nuffield Department of Orthopaedics, Rheumatology and Musculoskeletal Sciences, University of Oxford, Oxford, England

[2]Institute of Population Health Sciences, Barts and the London School of Medicine and Dentistry, Queen Mary University of London, London, England

[3]Institute of Applied Health Sciences, University of Aberdeen, Aberdeen, Scotland

[4]School of Health and Related Research, University of Sheffield, Sheffield, England

[5]School of Medicine & Keele Clinical Trials Unit (CTU), Keele University, Newcastle under Lyme, England

[6]Department of Health Research Methods, Evidence and Impact, McMaster University, Hamilton, Ontario, Canada

Authors emails

katie.mellor@ndorms.ox.ac.uk*

s.eddy@qmul.ac.uk

nicholas.peckham@ndorms.ox.ac.uk

c.m.bond@abdn.ac.uk

m.j.campbell@sheffield.ac.uk

g.Lancaster@keele.ac.uk

ThabanL@mcmaster.ca

s.eldridge@qmul.ac.uk

susan.dutton@csm.ox.ac.uk

sally.hopewell@csm.ox.ac.uk

*Corresponding author

For peer review only

For peer review only - http://bmjopen.bmj.com/site/about/guidelines.xhtml

ABSTRACT

Objectives: Prespecified progression criteria can inform the decision to progress from an external randomised pilot trial to a definitive randomised controlled trial. We assessed the characteristics of progression criteria reported in external randomised pilot trial protocols and results publications, including whether progression criteria were specified a priori and mentioned in prepublication peer review reports.

Study design: Methodological review.

Methods: We searched four journals through PubMed: *British Medical Journal Open, Pilot and Feasibility Studies, Trials and Public Library of Science One*. Eligible publications reported external randomised pilot trial protocols or results, were published between January 2018 and December 2019, and reported progression criteria. We double-data extracted 25% of the included publications. Here we report the progression criteria characteristics.

Results: We included 160 publications (123 protocols and 37 completed trials). Recruitment and retention were the most frequent indicators contributing to progression criteria. Progression criteria were mostly reported as distinct thresholds (e.g., achieving a specific target; 133/160, 83%). Less than a third of the planned and completed pilot trials that included qualitative research reported how these findings would contribute towards progression criteria (34/108, 31%). The publications seldom stated who established the progression criteria (12/160, 7.5%) or provided rationale or justification for progression criteria (44/160, 28%). Most completed pilot trials reported the intention to proceed to a definitive trial (30/37, 81%), but less than half strictly met all of their progression criteria (17/37, 46%). Prepublication peer review reports were available for 153/160 publications (96%). Peer review reports for 86/153 (56%) publications mentioned progression criteria, with peer reviewers of 35 publications commenting that progression criteria appeared not to be specified.

Conclusions: Many external randomised pilot trial publications did not adequately report or propose prespecified progression criteria to inform whether to proceed to a future definitive randomised controlled trial.

Review registration: Open Science Framework osf.io/bn35k

For peer review only

For peer review only - http://bmjopen.bmj.com/site/about/guidelines.xhtml

STRENGTHS AND LIMITATIONS OF THIS STUDY

- We conducted a large, recent assessment of the use and reporting of progression criteria in publications reporting external randomised pilot trial protocols and results.
- As this study only investigated external randomised pilot trial publications, it is unclear whether the findings can be generalised to other external feasibility study designs such as non-randomised pilot trials and non-pilot feasibility studies.
- One researcher independently screened all publications, assessed eligibility, and extracted data from all included publications, while other members of the research team provided a second data extraction for 25% of the included publications.

For peer review only

For peer review only - http://bmjopen.bmj.com/site/about/guidelines.xhtml

INTRODUCTION

Pilot trials aim to determine whether a future definitive randomised controlled trial (RCT) is feasible.[1] Internal pilot trials are embedded in the RCT design forming its first phase.[2] In contrast, external pilot trials are small stand-alone studies conducted before a definitive RCT. Prespecified progression criteria can help researchers interpret the findings of an external randomised pilot to decide whether the future definitive RCT is or is not feasible, and whether changes should be made to the trial design. Progression criteria should be specified before the pilot trial begins (a priori) to avoid introducing bias associated with establishing progression criteria once external pilot trial findings are known.

A 2019 review found that less than 20% of randomised pilot trial protocols published between 2013 and 2017 reported clear progression criteria. Trial features, such as a more recent publication year and certain countries of origin were associated with reporting progression criteria.[3] The 2016 Consolidated Standards of Reporting Trials (CONSORT) extension for reporting randomised pilot and feasibility trials advises that “at a minimum there should be something reported to suggest how the decision to progress to the definitive study will be made”.[4] The extent to which this guidance has improved progression criteria reporting in more recently published pilot trials is unclear. Although previous research has investigated whether progression criteria are reported, however the quality of progression criteria reporting – including how the criteria are established during pilot trial design and assessed upon pilot trial completion – has not yet been investigated.

We conducted a methodological review to investigate the application and reporting of progression criteria in a recent sample of external randomised pilot trial protocol and results publications. The primary objective was to describe the reporting of progression criteria, including the areas of feasibility that progression criteria were based on as described in a published framework of reasons for conducting pilot trials[5], their rationale or justification, and who established and assessed the progression criteria. One set of secondary objectives were to assess whether the progression criteria reported in pilot trial results publications were specified a priori in a published protocol or trial registration and whether the results publication reported the intention to progress to a definitive RCT. We also assessed the extent and context in which progression criteria were discussed in prepublication peer review reports where available for the protocol and results publications.

METHODS

Protocol and registration

A protocol for this research is registered on the Open Science Framework: osf.io/bn35k.[6] A summary of the methods used is detailed below. This review is reported following the PRISMA statement.[7]

Eligibility criteria

We included all protocol and results publications for external randomised pilot trials, that reported progression criteria and were published between January 2018 and December 2019 inclusive. Progression criteria were defined as criteria to inform the decision to progress to a definitive RCT. Included publications were published in the English language and were not restricted by intervention, health-related context or setting.

Information sources

For peer review only - http://bmjopen.bmj.com/site/about/guidelines.xhtml

Four journals were searched through PubMed: *British Medical Journal (BMJ) Open, Pilot and Feasibility Studies (PAFS), Trials and Public Library of Science (PLoS) One*. These journals were chosen because they are known to publish pilot trial protocol and results publications and had published the most PubMed indexed publications that included the terms 'pilot' or 'feasibility and 'trial' or 'protocol' in their title within 2018 and 2019. All included journals direct authors to the CONSORT statement[8] reporting guideline: BMJ Open and Trials advise authors to use the most appropriate statement extension, and the PAFS journal directs authors to the CONSORT Extension to Pilot and Feasibility Trials.[4]

Search terms included 'pilot' or 'feasibility' in the title, and 'trial', 'study' or 'protocol' in the title or abstract. See Supplementary file 1 for the full search strategy which was last used on 6 January 2020.

Study selection

Titles and abstracts of identified publications were screened against inclusion criteria. Full texts were retrieved for those that appeared relevant and screened against a predefined eligibility checklist by KM. All included publications were saved in EndNote X9 for Windows. Where both the protocol and corresponding pilot trial result publication were identified both were included.

Data collection

Data extraction forms produced in Microsoft Excel (Office16) were pre-piloted on the first 10 trials ordered alphabetically to ensure usability and completeness (the data extraction form used can be obtained from osf.io/fxv4n). One researcher (KM) extracted the data for all included publications. Other team members (SEd and NP) conducted a second data extraction for a randomly selected 25% sample. As we found minimal differences between the two data extractions, we decided not to conduct double data extraction for all of the included publications.

From trial protocol and results publications, we extracted: trial characteristics (including author, year, journal, country, randomisation design, therapeutic area, intervention type, sample size target, number of arms, and single or multicentre); feasibility objectives, outcomes, and instances of hypothesis testing; progression criteria details (wording, rationale or justification, format, process for establishing, and process for assessing); and references to progression criteria in prepublication peer review reports, where these were published online and linked to the publication.

For completed pilot trial results publications, we also extracted: whether progression criteria were met; any reported intention to progress to a definitive RCT; any proposed changes to the definitive RCT design; any refinement of hypotheses; any comment on data quality; and whether progression criteria had changed from the corresponding protocol or trial registration publication, if a published protocol was not available.

Synthesis of results

Descriptive statistics (frequencies and the mean, median and interquartile range for trial sample sizes) were produced to describe trial characteristics and address our primary and secondary objectives. Data were analysed using Stata version 15.0, StataCorp.[9] We report the frequency with which different feasibility uncertainties contributed to progression criteria using pre-specified domains of reasons for conducting pilot trials: process, resource, management and scientific.[5] The mean number of progression criteria specified per trial was also calculated.

We used narrative synthesis to describe the context in which progression criteria were mentioned in publicly available prepublication peer reviewer reports (synthesised by KM). We did not use a

For peer review only - http://bmjopen.bmj.com/site/about/guidelines.xhtml

predefined checklist to formally assess peer reviewer reports and we do not comment on the quality of peer review. Instead we simply looked for any mention that progression criteria were not present in the prepublication manuscript, and any queries about rationale for progression criteria used.

We did not aim to comment on the quality of the evidence from the studied randomised pilot trials. We aimed to comment only on the quality of reporting of progression criteria in this sample of external randomised pilot trial protocol and results publications.

Patient and public involvement

Patients or the public were not involved in the design, conduct, reporting, or dissemination plans of this research.

RESULTS

Study selection

Our search strategy identified 1030 publications. We screened their titles and abstracts, then assessed the full texts of 679 publications for eligibility. One hundred and sixty publications were eligible for our study. Figure 1 shows the full PRISMA flow chart of publications included and excluded at each stage. We excluded many publications during full text screening as we were unable to identify explicit progression criteria (n=251), or where publications only reported a recruitment or sample size target (n=118). Supplementary file 2 lists the included publications describing external pilot trial protocols and results. We found two instances where both the completed trial publication and protocol were identified. In these instances, both were included.

Study characteristics

Table 1 summarises the characteristics of the included publications. Most of the publications were pilot trial protocols (123/160, 77%) rather than completed pilot trial results (37/160, 23%). The journal with the most eligible publications was *Pilot and Feasibility Studies* (77/160, 48%). Most publications described external pilot trials that were two-arm (143/160, 89%), multicentre (102/160, 64%), non-industry funded (147/160, 92%) trials of counselling, lifestyle, or physiotherapy interventions (94/160, 59%). The reported trials covered 27 therapeutic areas and trials were from 18 countries, mostly from the UK (87/160, 54%).

Primary feasibility objectives were explicitly stated in 71/160 (44%) publications, and most publications reported feasibility outcomes in the methods that addressed all of the stated feasibility objectives (109/160, 68%). In 50/160 (31%) of the publications, the stated feasibility outcomes only somewhat addressed trial objectives, often because the objective stated was broad (e.g., "to determine whether a future trial is feasible") and did not define specific aspects of feasibility being assessed. With respect to data collection and assessment of feasibility outcomes, completely defined prespecified assessments or measurements were often stated (140/160, 88%). Most of the pilot trial publications that reported the intention to conduct hypothesis testing stated that this was exploratory or advised caution in interpretation. All but one publication reported multiple feasibility outcomes. The place in the publication where the specific uncertainties related to trial feasibility were first reported varied, but most often this was within the pilot trial feasibility objectives (72/160, 45%), or within the data collection section describing the feasibility outcomes (26/160, 16%) or pilot trial assessments or measurements (23/160, 14%).

Characteristics of progression criteria

For peer review only - http://bmjopen.bmj.com/site/about/guidelines.xhtml

Characteristics of progression criteria are presented in Table 2. The reported progression criteria generally addressed some (99/160, 62%) or all (53/160, 33%) of the pilot trial's feasibility outcomes. The pilot trial publications reported a mean of 4 (mean 4.05) progression criteria targets per pilot trial. Recruitment (113/160, 71%) and retention (106/160, 66%) were the most commonly reported indicators of feasibility to inform progression. In total we identified 58 distinct areas of trial feasibility that contributed to progression criteria, which we grouped into four domains: process, resource, management and scientific. The domains and areas are listed in Supplementary file 3. Most of the areas were process uncertainties (34/58, 59%), which dealt with the feasibility of processes that are key to the success of the future definitive RCT.[5]

Four publications reported progression criteria that were based on detecting potential efficacy, including determining non-inferiority of the intervention compared to a comparator, determining intervention superiority at follow-up, and finding a trend for difference between the intervention and comparator groups on clinical outcomes.

Progression criteria and quantitative indicators of feasibility

All of the pilot trial protocol and result publications reported using quantitative indicators of trial feasibility (e.g., rate of recruitment and amount of missing data) to inform at least one of the trial's progression criteria, with 78% (125/160) basing all progression criteria on quantitative indicators.

All but seven publications reported quantifiable numerical thresholds that were, or would be, used to assess the progression criteria. The seven remaining publications did not report specific quantifiable targets for progression criteria, but did report how the decision to progress from pilot to definitive RCT would be made and the feasibility indicators that would be considered when making this decision.

The quantifiable numerical targets used were most often reported as a distinct threshold (e.g., achieving a specified rate of recruitment, retention or data completion) (133/160, 83%). This was followed by a traffic light approach to reporting progression criteria (20/160, 13%) with thresholds correlating to different domains (e.g., above a higher threshold (green) indicating the definitive trial is feasible/proceed, within a mid/acceptable threshold (amber) indicating that changes to definitive trial are required, and below a lower threshold (red) indicating that the definitive trial is not feasible/not proceed).

Progression criteria and qualitative indicators of feasibility

Many publications reported planned or completed qualitative research as part of the randomised pilot trial (108/160, 68%). Although the findings from qualitative research conducted as part of a pilot trial are often reported in a separate publication, the intention to conduct qualitative research as part of a pilot trial should be made explicit before the pilot trial commences and be included in the pilot trial protocol.[10] The intention to conduct qualitative research was reported in protocols (89/123, 72%) more often than the results of qualitative research were reported in pilot trial result publications (19/37, 51%). However, qualitative indicators of trial feasibility, such as participants' or researchers' views of the acceptability of the trial or intervention collected in interviews, only informed progression criteria in 34 of the 108 (31%) publications that reported planned or completed qualitative research.

Two protocols reported multiple progression criteria for individual feasibility indicators, for example reporting both a target for number of participants recruited, and another target for number of participants recruited in a given timeframe. In one of these instances the authors reported that all criteria would need to be met or met within reasonable limits (within the green or amber traffic light domain) to progress to a full trial without major study re-design. It was unclear in the other protocol

For peer review only - http://bmjopen.bmj.com/site/about/guidelines.xhtml

For peer review only

whether meeting one criterion for each indicator of feasibility was sufficient justification for progression.

Process for establishing progression criteria

Twelve pilot trial publications reported how the progression criteria had been established, with most involving a trial steering or oversight committee (10/12, 83%; five reported having patient or public representation), working with funders (3/12, 25%) and/or a trial management group (5/12, 42%; two reported having patient or public representation). Other examples included agreeing progression criteria with a data monitoring and ethics committee (1/12, 8%; reported having patient or public representation) or study physicians (1/12, 8%) or establishing progression criteria based on the author's clinical experience (1/12, 8%).

Forty-four of the 160 publications (28%) reported rationale or justification for all or some of the stated progression criteria. For 29 publications the stated justification was previous related research, with 25 providing references to previous studies. Thirteen publications referenced sources of guidance and methodological research,[4,11–21] including three references to published guidance for internal pilot trials.[2] Four publications reported that contextual considerations had informed progression criteria (such as what would be an achievable recruitment rate, or intervention timeframe in the definitive trial), and three reported that clinical considerations had informed criteria (including medical chart reviews, clinical advice, and the nature of the population). Most of the pilot trial publications (116/160, 73%) did not report any rationale or justification for choice of progression criteria.

Process for assessing progression criteria

Nearly half of the publications (74/160, 46%) reported how progression criteria had or would inform the decision to progress to a future definitive RCT. This included whether changes to definitive RCT design would be considered if criteria were not strictly met (e.g., were met within reasonable limits or within the aforementioned "amber" traffic light range), or who was or would be involved in assessing progression criteria.

One publication reported a two-stage decision making process with different criteria assessed at each stage. Stage 1 was to decide on the best intervention route, and stage 2 was to decide whether to take the optimal intervention route forward to a definitive RCT. Another publication described the intention to hold a consensus conference of key stakeholders (patients, surgeons, public representatives and researchers) to agree whether a definitive RCT was feasible. Four pilot trials referred to the ADePT framework[11] to facilitate progression decision-making.

Nearly a quarter of publications reported who would be involved in assessing progression criteria (35/160, 22%). An independent trial steering committee was most commonly involved (26/35, 74%). Other reported parties included the research team or trial management group (13/35, 37%), data monitoring committee (7/35, 20%), trial sponsor (2/35, 6%), funder (1/35 3%), independent statistician (1/35, 3%), and other stakeholders, such as patients, clinicians and public representatives (3/35, 9%).

Intentions of completed randomised pilot trial publications

Most completed pilot trials reported that a future RCT would be feasible or the intention to proceed (30/37, 81%), including the seventeen completed pilot trials which met all of their progression criteria (Table 3). Thirteen pilot trials met some of their progression criteria, of these nine reported that a future RCT would be feasible, two reported that they would not proceed to a definitive RCT and two reported the intention to conduct further feasibility assessment. Four pilot trials did not meet their

For peer review only - http://bmjopen.bmj.com/site/about/guidelines.xhtml

progression criteria, of which three reported that a future RCT would still be feasible with changes to study design. The extent to which progression criteria was met was unclear for three trials, of these two reported the intention to conduct further feasibility assessment, and one reported that a future RCT would be feasible.

All but two of the completed pilot trials that reported that a future RCT would be feasible planned to make changes to their definitive RCT design (28/30, 93%). Of these, four reported the implications of the pilot trial findings in a table format, alongside whether progression criteria had or had not been met. Proposed changes included altering eligibility criteria, recruitment strategies (e.g., number of sites, recruitment materials, recruitment setting), randomisation design, blinding, outcome measures, follow up schedules and duration and seeking additional research team support (such as a dedicated trial manager, research coordinator and administrative team). It was unclear for two pilot trials whether changes would be made.

Four pilot trials reported definitive RCT funding intentions: Two NIHR Health Technology Assessment, one European and Developing Countries Clinical Trials Partnership, and one reported that a funding application had been prepared and submitted but did not specify the funder. One trial reported an anticipated progression timeframe, specifying a recruitment start year for the definitive RCT.

A priori progression criteria reporting of included randomised pilot trial publications

Trial protocols were available for 16 of the 37 (43%) completed randomised pilot trial publications (Table 3). Trial registrations were identified for 20 of the trials without a published protocol. We were unable to identify a published protocol or trial registration for the one remaining completed pilot trial.

Twenty-two published protocols or trial registrations for the 37 included completed pilot trials did not report progression criteria. An additional four protocols or trial registrations reported different progression criteria to the pilot trial result publication. Only one completed trial publication explained why the progression criteria had changed from the protocol: as the qualitative findings were reported in a separate publication, the progression criteria associated with acceptability were not included in the completed pilot trial result publication.

Progression criteria in prepublication peer reviewer reports

Prepublication peer review reports were available for 153 of the 160 (96%) included external pilot publications. Peer reviewer reports were not publicly available for the three *PLoS One* publications and peer review was not commissioned for four of the pilot trial protocols published in *BMJ Open*.

Table 2 shows that over half of the prepublication peer reviewer reports commented on progression criteria (86/153, 56%). Peer reviewer reports for 35 pilot trial publications (6 completed, 29 protocol) indicated that progression criteria were not reported in the submitted prepublication manuscript. Whether progression criteria were reported in the submitted prepublication manuscript was unclear for another five pilot trial publications.

Peer reviewer reports for 20 of the publications referred to the rationale or justification given for progression criteria. For example, they asked why a specific progression criterion was set, why progression criteria were given for specific outcomes, how the progression criteria were established, and how the progression decision was or will be made.

Peer reviewer reports for 26 pilot trials publications mentioned other aspects of progression criteria. For example, they mentioned changing where the progression criteria were reported in the manuscript (such as including the progression criteria in the publication abstract and not solely within

For peer review only - http://bmjopen.bmj.com/site/about/guidelines.xhtml

a supplementary file), clarifying ambiguous wording, adding percentages in brackets for clarity, correcting inconsistencies in the manuscript, and clarifying how specific criteria will be assessed. Reviewers also complemented authors for describing progression criteria well.

Not every author opted to update or add progression criteria to their manuscript after prepublication peer review. The authors of one publication argued that they could not alter their progression criteria because these criteria had been agreed by the trial management group, trial steering committee, and ethics committee. Other reasons that authors gave in response to peer review for not reporting quantifiable numerical targets for progression criteria included: they were not set during trial design; strict thresholds might be influenced by contextual variations that may not affect a future trial; progression criteria are best viewed as guidelines in line with the CONSORT extension statement; different perspectives could not be successfully captured by a set of criteria, and the trial is not an internal pilot.

DISCUSSION

Summary of main findings

Our study provides an assessment of the reporting of progression criteria in a large sample of external randomised pilot trials. We found that progression criteria varied widely and were not often justified, which agrees with recent research assessing the use of progression criteria in internal pilot trials.[22,23] Like internal pilots, many of the studied external randomised pilot trials reported the intention to proceed to a definitive RCT when they had not strictly met all progression criteria, demonstrating flexibility in approach to progression decision-making with many opting to make changes to the definitive trial design. It was unclear within the studied publications how, or by who, progression criteria are established and assessed.

Our findings suggest that guidance is needed to facilitate transparent and complete reporting of progression criteria a priori in external pilot trial protocols[3] and in pilot trial results publications.[24]

Strengths and weaknesses of the study

A study strength is our extensive recent sample of 160 publications reporting external randomised pilot trials in four key journals that are known to publish pilot trials.

A limitation of this study is that single screening was used, and double data extraction was only conducted for 25% of the included publications. However, since only minimal data extraction differences were identified we decided not to conduct double data extraction for all included publications.

We only included external randomised pilot trials and it is unclear whether these findings are generalisable to other external feasibility study designs, such as non-randomised pilot trials and non-pilot feasibility studies. In addition, our findings are limited to four included journals and we did not include publications of non-English language which could introduce potential language bias.[25]

In addition, our review of peer reviewer reports to assess the context in which progression criteria were mentioned was subject to interpretation. Prepublication peer review reports were not available for all included publications: *PLoS One* allows authors to opt-in to publish peer review reports, and peer review was not commissioned for four pilot trial protocols published in *BMJ Open* that had already been peer reviewed for ethical and funding approval before submission. Progression criteria

For peer review only - http://bmjopen.bmj.com/site/about/guidelines.xhtml

might also have been added or altered based on editorial review before peer review. Unlike peer reviewer reports, it is not common practice to make editorial review publicly available.

Meaning of the study: Possible explanations and implications for clinicians and policymakers

Our findings suggest that the research community are uncertain about how progression criteria should be applied to external randomised pilot trials and how this should be reported in protocol and results publications. We identified one instance within a peer reviewers report for a pilot trial protocol where authors had stated that progression criteria were not set because the trial was not an internal pilot and as such would not immediately progress to a fully powered RCT.

We found recruitment and retention rates to be the most common feasibility uncertainties to contribute towards progression criteria. This result is supported by a recent review finding recruitment to be the most common uncertainty evaluated in surgical pilot and feasibility studies,[26] and is unsurprising considering that recruiting to target is a challenge for many RCTs.[27] Fairhurst and colleagues suggested that researchers conducting feasibility studies might focus on feasibility uncertainties that are perceived to be important to funders.[26] In support of this suggestion, we found that other feasibility uncertainties that are equally as important to trial success, such as intervention acceptability, contributed to progression criteria much less often than recruitment and retention.

We found that peer review improved the reporting of pilot trials, for example by prompting authors to explain their progression criteria and rationale. However, we also identified instances where new progression criteria were likely added as a result of peer review, in both protocols and pilot trial result manuscripts. Adding post-hoc progression criteria, could introduce bias since progression criteria might be based on targets that have been met or exceeded to justify progression to a definitive RCT.

Unanswered questions and future research

The processes for establishing and assessing progression criteria were not commonly reported, leaving unanswered questions about how the decision to progress from pilot to definitive RCT is made in practice. This underreporting could be due to a lack of guidance around best practices for progression of external randomised pilot trials, and how this should be reported in pilot trial publications. To expand on these findings a qualitative research study is being conducted to explore different stakeholders' perspectives of using progression criteria to inform the decision to progress from an external randomised pilot trial to a definitive randomised controlled trial in practice.[28] Our findings also highlight the importance of journal editor and peer reviewer endorsement of evidence-based guidelines to improve reporting standards. The development of evidence-based guidance specific to the application and reporting of progression criteria in external pilot trials, for both protocols and completed trials, is a research priority. This finding is timely, as the UK's biggest funder of pilot and feasibility studies, NIHR Research for Patient Benefit, now stipulates that a clear route of progression (e.g., progression criteria) should be included in pilot and feasibility study funding applications.[29] A further possible area of investigation is whether research ethics committees can and should comment on progression criteria in research ethics applications. Researchers have an ethical obligation to conduct research with integrity and transparency. Defining a priori progression criteria and adequately reporting them helps to uphold the integrity and transparency of the external randomised pilot trial's progression.

Conclusions

For peer review only - http://bmjopen.bmj.com/site/about/guidelines.xhtml

We found heterogeneity in the reporting of progression criteria in external randomised pilot trial publications. It was often unclear how progression criteria were established, upon what justification or rationale they were based, how they were or will be assessed, and who is involved at each stage. Peer reviewers often commented on progression criteria, questioning whether these criteria were established a priori, as is recommended for good practice. Clear, evidence-based recommendations for the use and reporting of progression criteria in external randomised pilot trials are required. Guidance to this effect would benefit researchers, peer reviewers, journal editors, and funders of external randomised pilot trials, and inform the design of subsequent definitive trials. In the meantime we suggest researchers consider reporting how their progression criteria were established in their pilot trial protocol publications, and how their findings in relation to progression criteria have informed progression decision-making and the subsequent definitive trial design in pilot trial results publications.

ACKNOWLEDGEMENTS

We acknowledge Dr Jennifer A de Beyer, Centre for Statistics in Medicine, University of Oxford for English language editing.

FOOTNOTES

Twitter: @katiejmellor

Contributors: This study was conceived and designed as part of KMs doctoral thesis under the supervision of SH, SD and SE. CB, MC, GL and LT provided feedback on the study design during protocol development. KM collected and analysed the data. SEd and NP conducted double data extraction. KM drafted the manuscript. All authors reviewed and commented on manuscript drafts. All authors approved the final manuscript.

Funding: This work was supported by Medical Research Council Doctoral Training Partnership funding [grant number MR/N013468/1] awarded to KM.

Competing interests: KM, SEd, CB, MC, GL, LT, SE and SH contribute to a Pilot and Feasibility Studies working group. GL and LT are Editors in Chief of the Pilot and Feasibility Studies journal. SE, MC and CB are editors of the Pilot and Feasibility Studies journal.

Data sharing: Data will be made available upon request and included in a DPhil thesis published open access through the Oxford University Archive upon KM's DPhil completion.

Ethics approval statement: Ethics committee approval is not required for this review since only previously published data is included.

For peer review only

For peer review only - http://bmjopen.bmj.com/site/about/guidelines.xhtml

FIGURES AND TABLES

Figure 1 PRISMA flow chart

For peer review only

For peer review only - http://bmjopen.bmj.com/site/about/guidelines.xhtml

Table 1 Characteristics of the studied external randomised pilot trial publications

	Completed (n=37) n (%)	Protocol (n=123) n (%)	Total (n=160) n (%)
**Journal**			
British Medical Journal (BMJ) Open	11 (30%)	34 (28%)	45 (28%)
Pilot and Feasibility Studies (PAFS)	21 (57%)	56 (46%)	77 (48%)
Trials	2 (5%)	33 (27%)	35 (22%)
Public Library of Science (PLoS) One	3 (8%)	0 (0%)	3 (2%)
**Country**			
Australia	6 (16%)	4 (3%)	10 (6%)
Brazil	0 (0%)	1 (1%)	1 (1%)
Canada	4 (11%)	15 (12%)	19 (12%)
China	0 (0%)	4 (3%)	4 (3%)
Denmark	0 (0%)	1 (1%)	1 (1%)
Germany	1 (3%)	1 (1%)	2 (1%)
Korea	0 (0%)	1 (1%)	1 (1%)
Nepal	1 (3%)	2 (2%)	3 (2%)
New Zealand	2 (5%)	1 (1%)	3 (2%)
Norway	1 (3%)	0 (0%)	1 (1%)
Republic of Ireland	0 (0%)	5 (4%)	5 (3%)
Sweden	1 (3%)	1 (1%)	2 (1%)
Tanzania	0 (0%)	1 (1%)	1 (1%)
Thailand	0 (0%)	1 (1%)	1 (1%)
The Netherlands	0 (0%)	2 (2%)	2 (1%)
UK	19 (51%)	68 (55%)	87 (54%)
USA	2 (5%)	14 (11%)	16 (10%)
Zimbabwe	0 (0%)	1 (1%)	1 (1%)
**Funder**			
Industry	2 (5%)	2 (2%)	4 (3%)
Non-industry	32 (86%)	115 (94%)	147 (92%)
A combination	1 (3%)	4 (3%)	5 (3%)
Unknown	2 (5%)	1 (1%)	3 (2%)
Trial did not receive funding	0 (0%)	1 (1%)	1 (1%)
**Therapeutic areas**			
Alternative medicine	0 (0%)	1 (1%)	1 (1%)
Anaesthesia	1 (3%)	1 (1%)	2 (1%)
Cardiology	0 (0%)	3 (2%)	3 (2%)
Critical Care	1 (3%)	7 (6%)	8 (5%)
Endocrinology	0 (0%)	6 (5%)	6 (4%)
Gastroenterology	1 (3%)	3 (2%)	4 (3%)
Geriatrics	1 (3%)	4 (3%)	5 (3%)
Hepatology	0 (0%)	1 (1%)	1 (1%)
Infectious diseases	0 (0%)	3 (2%)	3 (2%)
Musculoskeletal	6 (16%)	4 (3%)	10 (6%)
Nephrology	0 (0%)	1 (1%)	1 (1%)
Neurology	3 (8%)	12 (10%)	15 (9%)
Obstetrics/Gynaecology	2 (5%)	6 (5%)	8 (5%)
Oncology	4 (11%)	7 (6%)	11 (7%)
Ophthalmology	0 (0%)	1 (1%)	1 (1%)
Orthopaedics	2 (5%)	1 (1%)	3 (2%)

For peer review only - http://bmjopen.bmj.com/site/about/guidelines.xhtml
For peer review only

Other	2 (5%)	3 (2%)	5 (3%)
Otolaryngology (ENT)	2 (5%)	1 (1%)	3 (2%)
Paediatrics	2 (5%)	3 (2%)	5 (3%)
Pain	1 (3%)	0 (0%)	1 (1%)
Palliative care	0 (0%)	3 (2%)	3 (2%)
Psychiatry/Psychology	2 (5%)	19 (15%)	21 (13%)
Public Health	2 (5%)	15 (12%)	17 (11%)
Respiratory	0 (0%)	2 (2%)	2 (1%)
Rheumatology	0 (0%)	1 (1%)	1 (1%)
Surgery	3 (8%)	8 (7%)	11 (7%)
Trauma	2 (5%)	7 (6%)	9 (6%)
**Intervention type**			
Drug	4 (11%)	9 (7%)	13 (8%)
Surgery/procedure	6 (16%)	13 (11%)	19 (12%)
Counselling/lifestyle/physiotherapy	22 (59%)	72 (59%)	94 (59%)
Equipment	4 (11%)	5 (4%)	9 (6%)
Other	1 (3%)	24 (20%)	25 (16%)
**Sample size target ***			
Mean (SD)	72.8 (62.5)	258.5 (1215.7)	217.3 (1074.9)
Median (IQR)	60 (32-90)	60 (40-100)	60 (40-100)
Min-Max	6-300	20-12000	6-12000
**Cluster randomised pilot trials**	**(n=3)**	**(n=18)**	**(n=21)**
Number of clusters			
Mean	7.3 (2.3)	9.7 (11.6)	9.3 (10.7)
Median (IQR)	6 (6-10)	6 (3-10)	6 (4-10)
Min-Max	6-10	2-45	2-45
**Number of arms**			
2	32 (86%)	111 (90%)	143 (89%)
>2	5 (14%)	12 (10%)	17 (11%)
**Number of centres**			
Single centre	19 (51%)	36 (29%)	55 (34%)
Multi-centre	18 (49%)	84 (68%)	102 (64%)
Unclear	0 (0%)	3 (2%)	3 (2%)
**Feasibility objective/s explicitly described as primary**			
Yes	9 (24%)	62 (50%)	71 (44%)
No	28 (76%)	61 (50%)	89 (56%)
**Trial outcomes address trial objectives**			
Yes	18 (49%)	91 (74%)	109 (68%)
No	0 (0%)	1 (1%)	1 (1%)
Somewhat**	19 (51%)	31 (25%)	50 (31%)
**Completely defined prespecified assessments or measurements stated**			
Yes	27 (73%)	113 (92%)	140 (88%)
Not for every outcome	10 (27%)	10 (8%)	20 (13%)
**Hypothesis testing**			
Yes	2 (5%)	16 (13%)	18 (11%)
Yes, exploratory/caution advised	18 (49%)	43 (35%)	61 (38%)
No	17 (46%)	64 (52%)	81 (51%)

For peer review only

**Number of uncertainties reported**			
One	0 (0%)	1 (1%)	1 (1%)
Multiple	37 (100%)	122 (99%)	159 (99%)
**Where uncertainties are first reported (excluding abstract)**			
Introduction	0 (0%)	1 (1%)	1 (1%)
Research question(s)	2 (5%)	4 (3%)	6 (4%)
Aim(s)	5 (14%)	16 (13%)	21 (13%)
Objective(s)	10 (27%)	62 (50%)	72 (45%)
Outcome(s)	9 (24%)	17 (14%)	26 (16%)
Outcome measure(s)	7 (19%)	16 (13%)	23 (14%)
Analysis	0 (0%)	2 (2%)	2 (1%)
Within the text under a feasibility/uncertainty heading	2 (5%)	1 (1%)	3 (2%)
Throughout the text, not in one specific area	2 (5%)	4 (3%)	6 (4%)

Percentages may not sum up to 100 due to rounding.

**Where publications reported a sample size target range (e.g., 12-16 participants), the lower bound of the target is included. A sample size target was not reported in two publications (both reporting completed pilot trials and including the actual number of recruited participants).*

***Trial objective was vague (e.g., to "assess feasibility") and the specific areas of feasibility were not explicitly stated.*

For peer review only

For peer review only - http://bmjopen.bmj.com/site/about/guidelines.xhtml

BMJ Open manuscript v2.0, resubmission

Table 2 Characteristics of progression criteria reported in external randomised pilot trial publications

	Completed (n=37) n (%)	Protocol (n=123) n (%)	Total (n=160) n (%)
**Feasibility outcomes informing progression criteria**			
All	14 (38%)	39 (32%)	53 (33%)
Some	22 (59%)	77 (63%)	99 (62%)
None	1 (3%)	2 (2%)	3 (2%)
Unclear*	0 (0%)	5 (4%)	5 (3%)
**Reported process for establishing progression criteria**			
**Who decided on progression criteria**			
Reported	4 (11%)	8 (6%)	12 (8%)
Not reported	33 (89%)	115 (94%)	148 (93%)
**Rationale for progression criteria**			
Reported for all progression criteria	8 (22%)	20 (16%)	28 (18%)
Reported for some criteria only	4 (11%)	12 (10%)	16 (10%)
Not reported	25 (68%)	91 (74%)	116 (73%)
**Progression criteria format**			
**Research method informing progression criteria**			
Quantitative	32 (86%)	93 (76%)	125 (78%)
Quantitative and qualitative (mixed methods)	5 (14%)	29 (24%)	34 (21%)
Unclear	0 (0%)	1 (1%)	1 (1%)
**Qualitative research contribution**			
Informs progression criteria	5 (14%)	29 (24%)	34 (21%)
Does not inform progression criteria	14 (38%)	60 (49%)	74 (46%)
Qualitative research methodology not used	18 (49%)	34 (28%)	52 (33%)
**Quantitative progression criteria target format**			
Distinct threshold	34 (92%)	99 (80%)	133 (83%)
Traffic light system	2 (5%)	18 (15%)	20 (13%)
Other	1 (3%)	6 (5%)	7 (4%)
**Reported process for assessing progression criteria to inform the progression decision**			
**Process for progression decision making**			
Reported	16 (43%)	58 (47%)	74 (46%)
Not reported	21 (57%)	65 (53%)	86 (54%)
**Who is involved in assessing progression criteria**			
Reported	5 (14%)	30 (24%)	35 (22%)
Not reported	32 (86%)	93 (76%)	125 (78%)
**Peer reviewers' reports**			
**Progression criteria mentioned in peer reviewers report**			
Yes	19 (51%)	67 (54%)	86 (54%)
Peer review comment theme			
Progression criteria were not specified	*6 (32%)*	*29 (44%)*	*35 (41%)*
Unclear whether progression criteria were specified	*1 (5%)*	*4 (6%)*	*5 (6%)*
Progression criteria rationale or justification query	*5 (26%)*	*15 (22%)*	*20 (23%)*
Other	*7 (37%)*	*19 (29%)*	*26 (30%)*
No	15 (41%)	52 (42%)	67 (42%)
Peer reviewers report unavailable	3 (8%)	4 (3%)	7 (4%)

Percentages may not sum up to 100 due to rounding.

For peer review only - http://bmjopen.bmj.com/site/about/guidelines.xhtml

**Feasibility uncertainties are not completely defined in the objectives and outcomes; key methodological uncertainties have been identified from those stipulated in the progression criteria*

For peer review only

For peer review only - http://bmjopen.bmj.com/site/about/guidelines.xhtml

Table 3 Intensions reported in completed external randomised pilot trial results publications

Data	Completed (n=37) n (%)
**Progression criteria met**	
All	17 (46%)
None	4 (11%)
Some	13 (35%)
Unclear	3 (8%)
**Progression decision**	
Proceed/future RCT is feasible	30 (81%)
With intended design	*0 (0%)*
With amendments	*28 (93%)*
Not reported whether changes will be made to definitive RCT design	*2 (7%)*
Funding intentions	
Funding for definitive RCT identified	*4 (13%)*
Non-industry	*3 (75%)*
Unclear	*1 (25%)*
Expected funding for definitive RCT not reported	*26 (87%)*
Timing intentions	
Time frame of expected progression reported	*1 (3%)*
Time frame for expected progression not reported	*29 (97%)*
Conduct further pilot/feasibility work	4 (11%)
Not proceed/future RCT is not feasible	3 (8%)
**Justification reported for the progression decision reported**	
Yes	36 (97%)
No	1 (3%)
**Comment on data quality (e.g., proportion of missing/incomplete data from questionnaires or results)**	
Yes	27 (73%)
No	10 (27%)
**Comment on refinement of hypotheses**	
Yes	1 (3%)
No	36 (97%)
**Published protocol available**	
Yes	16 (43%)
No	1 (3%)
Alternative available e.g., trial registration or REC submission	20 (54%)
**Progression criteria in earlier trial record (protocol or registration)**	
No change	10 (28%)
Yes changed	26 (72%)
Reasons for change reported	*1 (4%)*
No reason for change reported	*3 (12%)*
Progression criteria were not reported in the earlier trial record	*22 (85%)*

Percentages may not sum up to 100 due to rounding.

For peer review only

For peer review only - http://bmjopen.bmj.com/site/about/guidelines.xhtml

REFERENCES

1 Eldridge SM, Lancaster GA, Campbell MJ, *et al.* Defining Feasibility and Pilot Studies in Preparation for Randomised Controlled Trials: Development of a Conceptual Framework. *PLoS One* 2016;**11**:e0150205. doi:10.1371/journal.pone.0150205

2 Avery KNL, Williamson PR, Gamble C, *et al.* Informing efficient randomised controlled trials: Exploration of challenges in developing progression criteria for internal pilot studies. *BMJ Open* 2017;**7**. doi:10.1136/bmjopen-2016-013537

3 Mbuagbaw L, Kosa SD, Lawson DO, *et al.* The reporting of progression criteria in protocols of pilot trials designed to assess the feasibility of main trials is insufficient: a meta-epidemiological study. *Pilot feasibility Stud* 2019;**5**:120. doi:10.1186/s40814-019-0500-z

4 Eldridge SM, Chan CL, Campbell MJ, *et al.* CONSORT 2010 statement: Extension to randomised pilot and feasibility trials. *BMJ* 2016;**355**:i5239. doi:10.1136/bmj.i5239

5 Thabane L, Ma J, Chu R, *et al.* A tutorial on pilot studies: the what, why and how. *BMC Med Res Methodol* 2010;**10**:1. doi:10.1186/1471-2288-10-1

6 Mellor K, Hopewell S. An investigation of the current use of progression criteria in external randomised pilot studies: a systematic review protocol. *OSF* Published Online First: 7 May 2020. doi:10.17605/OSF.IO/BN35K

7 Moher D, Liberati A, Tetzlaff J, *et al.* Preferred Reporting Items for Systematic Reviews and Meta-Analyses: The PRISMA Statement. *PLoS Med* 2009;**6**:e1000097. doi:10.1371/journal.pmed.1000097

8 Schulz KF, Altman DG, Moher D. CONSORT 2010 Statement: Updated guidelines for reporting parallel group randomised trials. *BMJ* 2010;**340**:698–702. doi:10.1136/bmj.c332

9 Stata Statistical Software: Release 15. StataCorp. 2017.

10 Cooper C, O'Cathain A, Hind D, *et al.* Conducting qualitative research within Clinical Trials Units: Avoiding potential pitfalls. *Contemp Clin Trials* 2014;**38**:338–43. doi:10.1016/j.cct.2014.06.002

11 Bugge C, Williams B, Hagen S, *et al.* A process for Decision-making after Pilot and feasibility Trials (ADePT): Development following a feasibility study of a complex intervention for pelvic organ prolapse. *Trials* 2013;**14**:1. doi:10.1186/1745-6215-14-353

12 Perepletchikova F, Kazdin AE. Treatment integrity and therapeutic change: Issues and research recommendations. *Clin Psychol Sci Pract* 2005;**12**:365–83.

13 Sidani S, Braden CJ. *Design, evaluation, and translation of nursing interventions*. John Wiley & Sons 2011.

14 Tickle-degnen L. Nuts and bolts of conducting feasibility studies. *Am J Occup Ther* 2013;**67**:1–7. doi:10.5014/ajot.2013.006270

15 Hertzog MA. Considerations in Determining Sample Size for Pilot Studies. *Wiley Period Inc Res Nurs Heal* 2008;:180–191. doi:10.1002/nur.20247

16 Lee EC, Whitehead AL, Jacques RM, *et al.* The statistical interpretation of pilot trials: Should significance thresholds be reconsidered? *BMC Med Res Methodol* 2014;**14**. doi:10.1186/1471-2288-14-41

17 Julious S, Tan SB, Machin D. *An Introduction to Statistics in Early Phase Trials - Steven Julious,*

For peer review only - http://bmjopen.bmj.com/site/about/guidelines.xhtml

Say Beng Tan, David Machin - Google Books. Wiley-Blackwell 2010.

18 Eldridge SM, Costelloe CE, Kahan BC, *et al.* How big should the pilot study for my cluster randomised trial be? *Stat Methods Med Res* 2016;**25**:1039–56. doi:10.1177/0962280215588242

19 Altman DG, Bland JM. Measurement in Medicine: The Analysis of Method Comparison Studies. *J R Stat Soc Ser D (The Stat* 1983;**32**:307–17. doi:10.2307/2987937

20 Baltussen R, Niessen L. Priority setting of health interventions: The need for multi-criteria decision analysis. *Cost Eff Resour Alloc* 2006;**4**:14. doi:10.1186/1478-7547-4-14

21 Polit DF, Beck CT. *Nursing research: Principles and methods*. Lippincott Williams & Wilkins 2004.

22 Herbert E, Julious SA, Goodacre S. Progression criteria in trials with an internal pilot: an audit of publicly funded randomised controlled trials. *Trials* 2019;**20**:1–9. doi:10.1186/s13063-019-3578-y

23 Rosala-Hallas A, Gamble C, Blazeby J, *et al.* A review of current practice in the design and assessment of internal pilots in UK NIHR clinical trials. *Trials* 2019;**20**:1–5. doi:10.1186/s13063-019-3669-9

24 Hallingberg B, Turley R, Segrott J, *et al.* Exploratory studies to decide whether and how to proceed with full-scale evaluations of public health interventions: a systematic review of guidance. *Pilot Feasibility Stud* 2018;**4**:104. doi:10.1186/s40814-018-0290-8

25 Neimann Rasmussen L, Montgomery P. The prevalence of and factors associated with inclusion of non-English language studies in Campbell systematic reviews: A survey and meta-epidemiological study. *Syst Rev* 2018;**7**:1–12. doi:10.1186/s13643-018-0786-6

26 Fairhurst K, Blazeby JM, Potter S, *et al.* Value of surgical pilot and feasibility study protocols. *Br J Surg* 2019;**106**:968–78. doi:10.1002/bjs.11167

27 Walters SJ, Dos Anjos Henriques-Cadby IB, Bortolami O, *et al.* Recruitment and retention of participants in randomised controlled trials: A review of trials funded and published by the United Kingdom Health Technology Assessment Programme. *BMJ Open* 2017;**7**:e015276. doi:10.1136/bmjopen-2016-015276

28 Mellor K, Albury C, Hopewell S. Using progression criteria to determine feasibility of external randomised pilot trials: protocol for a qualitative study of stakeholder views. Published Online First: 4 December 2020. doi:10.17605/OSF.IO/5N2KZ

29 NIHR. Guidance on applying for feasibility studies. v1.0 Jul 2017. 2019.https://www.nihr.ac.uk/documents/nihr-research-for-patient-benefit-rfpb-programme-guidance-on-applying-for-feasibility-studies/20474 (accessed 13 Nov 2019).

For peer review only - http://bmjopen.bmj.com/site/about/guidelines.xhtml

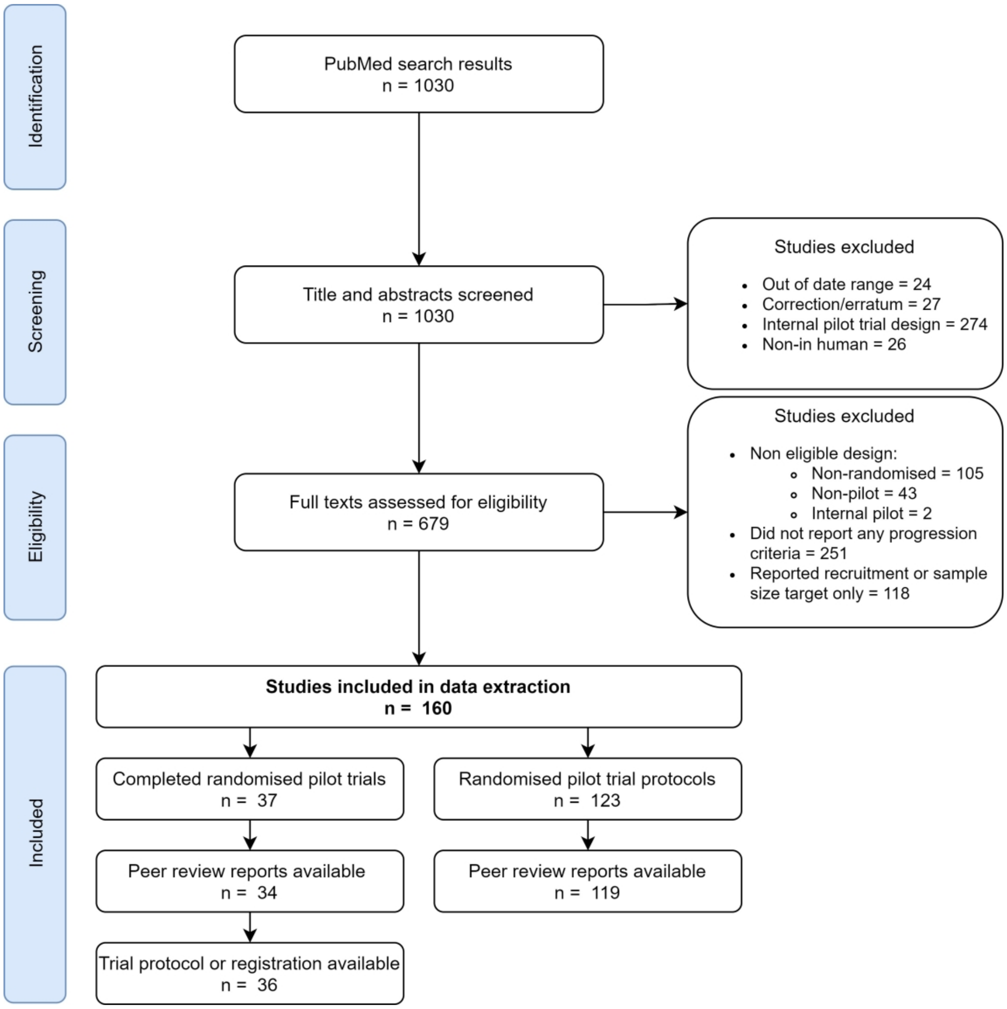

Figure 1 PRISMA flow chart

1148x1148mm (72 x 72 DPI)

SUPPLEMENTARY FILE 1: SEARCH STRATEGY

[PubMed search 06 Jan 2020]

No	Search terms	Results
1	**Pilot[Title]**	68704
2	**Feasibility[Title]**	31478
3	1 OR 2	98358
4	**Trial[Title/Abstract]**	576760
5	**Study[Title/Abstract]**	7478840
6	**Protocol[Title/Abstract]**	300466
7	4 OR 5 OR 6	7872797
8	**(((("Pilot and feasibility studies"[Journal])) OR "Trials"[Journal]) OR "BMJ open"[Journal]) OR "PloS one"[Journal]**	245189
9	3 AND 7 AND 8	2529
10	**9 AND 2018.01.01"[Date - Publication] : "2019.12.31"[Date - Publication]**	1030

For peer review only

SUPPLEMENTARY FILE 2: LIST OF INCLUDED STUDIES

First author	Year	Journal	Publication title
Ali	2018	*BMJ Open*	Individual cognitive stimulation therapy for people with intellectual disability and dementia: Protocol of a feasibility randomised controlled trial
Battle	2019	*BMJ Open*	A multicentre randomised feasibility STUdy evaluating the impact of a prognostic model for Management of BLunt chest wall trauma patients: STUMBL Trial
Collings	2019	*BMJ Open*	INSoles to Ease Pressure (INSTEP) Study: A multicentre, randomised controlled feasibility study to compare the effectiveness of a novel instant optimised insole with a standard insole for people with diabetic neuropathy: A study protocol
Dean	2018	*BMJ Open*	Community-based rehabilitation training after stroke: Results of a pilot randomised controlled trial (ReTrain) investigating acceptability and feasibility
Dinneen	2019	*BMJ Open*	NeuroSAFE robot-assisted laparoscopic prostatectomy versus standard robot-assisted laparoscopic prostatectomy for men with localised prostate cancer (NeuroSAFE PROOF): Protocol for a randomised controlled feasibility study
Edwards	2019	*BMJ Open*	Novel ACT-based eHealth psychoeducational intervention for students with mental distress: A study protocol for a mixed-methodology pilot trial
Froghi	2019	*BMJ Open*	Ward-based Goal-Directed Fluid Therapy (GDFT) in Acute Pancreatitis (GAP) trial: Study protocol for a feasibility randomised controlled trial
Furlano	2019	*BMJ Open*	Feasibility of a 6-month pilot randomised controlled trial of resistance training on cognition and brain health in Canadian older adults at-risk for diabetes: Study protocol
Geraghty	2018	*BMJ Open*	Using an internet intervention to support self-management of low back pain in primary care: Findings from a randomised controlled feasibility trial (SupportBack)
Griffin	2019	*BMJ Open*	Healthy Dads, Healthy Kids UK, a weight management programme for fathers: Feasibility RCT
Guagliano	2019	*BMJ Open*	Whole family-based physical activity promotion intervention: The Families Reporting Every Step to Health pilot randomised controlled trial protocol
Harper	2018	*BMJ Open*	Treatment of fatigue with physical activity and behavioural change support in vasculitis: Study protocol for an open-label randomised controlled feasibility study
Hawley-Hague	2019	*BMJ Open*	Can smartphone technology be used to support an effective home exercise intervention to prevent falls amongst community dwelling older adults?: The TOGETHER feasibility RCT study protocol

For peer review only

Hughes	2018	*BMJ Open*	Prediabetes in pregnancy, can early intervention improve outcomes? A feasibility study for a parallel randomised clinical trial
Jolly	2018	*BMJ Open*	Protocol for a feasibility trial for improving breast feeding initiation and continuation: Assets-based infant feeding help before and after birth (ABA)
Jones	2019	*BMJ Open*	Walk, Talk and Listen: A pilot randomised controlled trial targeting functional fitness and loneliness in older adults with hearing loss
Keene	2019	*BMJ Open*	Progressive functional exercise versus best practice advice for adults aged 50 years or over after ankle fracture: Protocol for a pilot randomised controlled trial in the UK - The Ankle Fracture Treatment: Enhancing Rehabilitation (AFTER) study
Lewis	2019	*BMJ Open*	Cuff Leak Test and Airway Obstruction in Mechanically Ventilated ICU Patients (COMIC): A pilot randomised controlled trial protocol
Limond	2019	*BMJ Open*	Clinical and cost-effectiveness of teen online problem-solving for adolescents who have survived an acquired brain injury in the UK: Protocol for a randomised, controlled feasibility study (TOPS-UK)
Lockstone	2019	*BMJ Open*	Non-Invasive Positive airway Pressure thErapy to Reduce Postoperative Lung complications following Upper abdominal Surgery (NIPPER PLUS): protocol for a single-centre, pilot, randomised controlled trial
Lorenzini	2019	*BMJ Open*	Measuring changes in device use of a head-mounted low vision aid after personalised telerehabilitation: Protocol for a feasibility study
McIntyre	2018	*BMJ Open*	FLUID trial: A protocol for a hospital-wide open-label cluster crossover pragmatic comparative effectiveness randomised pilot trial
Mcpherson	2019	*BMJ Open*	Children and teens in charge of their health (catch): A protocol for a feasibility randomised controlled trial of solution-focused coaching to foster healthy lifestyles in childhood disability
Morris	2019	*BMJ Open*	Dietary Approaches to the Management of type 2 Diabetes (DIAMOND): Protocol for a randomised feasibility trial
Munce	2019	*BMJ Open*	Ontario Brain Injury Association Peer Support Program: A mixed methods protocol for a pilot randomised controlled trial
Neves	2019	*BMJ Open*	Protocol for a feasibility study of a cohort embedded randomised controlled trial comparing NEphron Sparing Treatment (NEST) for small renal masses
O'Connor	2019	*BMJ Open*	SAFETEL randomised controlled feasibility trial of a safety planning intervention with follow-up telephone contact to reduce suicidal behaviour: Study protocol
Orkin	2019	*BMJ Open*	Protocol for a mixed-methods feasibility study for the surviving opioid overdose with naloxone education and resuscitation (SOONER) randomised control trial

For peer review only

Pai	2019	*BMJ Open*	Protocol for a double-blind, randomised, placebo-controlled pilot study for assessing the feasibility and efficacy of faecal microbiota transplant in a paediatric Crohn's disease population: PediCRaFT Trial
Papathanassoglou	2019	*BMJ Open*	Relaxation for Critically ill Patient Outcomes and S tress-coping Enhancement (REPOSE): A protocol for a pilot randomised trial of an integrative intervention to improve critically ill patients' delirium and related outcomes
Pennington	2019	*BMJ Open*	Internet delivery of intensive speech and language therapy for children with cerebral palsy: A pilot randomised controlled trial
Pouw	2018	*BMJ Open*	Hospital at Home care for older patients with cognitive impairment: A protocol for a randomised controlled feasibility trial
Quraishi	2019	*BMJ Open*	STOP-Colitis pilot trial protocol: A prospective, open-label, randomised pilot study to assess two possible routes of faecal microbiota transplant delivery in patients with ulcerative colitis
Reddington	2018	*BMJ Open*	Does early intervention improve outcomes in the physiotherapy management of lumbar radicular syndrome? Results of the POLAR pilot randomised controlled trial
Ribeiro	2019	*BMJ Open*	Effectiveness of a tailored rehabilitation versus standard strengthening programme for patients with shoulder pain: A protocol for a feasibility randomised controlled trial (the Otago MASTER trial)
Schults	2018	*BMJ Open*	Normal saline instillation versus no normal saline instillation and lung Recruitment versus no lung recruitment with paediatric Endotracheal Suction: The NARES trial. A study protocol for a pilot, factorial randomised controlled trial
Sharma	2018	*BMJ Open*	Pain education for patients with non-specific low back pain in Nepal: Protocol of a feasibility randomised clinical trial (PEN-LBP Trial)
Sharma	2019	*BMJ Open*	Results of a feasibility randomised clinical trial on pain education for low back pain in Nepal: The Pain Education in Nepal-Low Back Pain (PEN-LBP) feasibility trial
Steare	2019	*BMJ Open*	App to support Recovery in Early Intervention Services (ARIES) study: Protocol of a feasibility randomised controlled trial of a self-management Smartphone application for psychosis
Sugg	2018	*BMJ Open*	Morita Therapy for depression (Morita Trial): A pilot randomised controlled trial
Thyer	2018	*BMJ Open*	Randomised controlled feasibility trial of the Active Communication Education programme plus hearing aid provision versus hearing aid provision alone (ACE to HEAR): A study protocol
Wall	2018	*BMJ Open*	Safety and feasibility evaluation of tourniquets for total knee replacement (SAFE-TKR): Study protocol
Wiangkham	2019	*BMJ Open*	Pragmatic cluster randomised double-blind pilot and feasibility trial of an active behavioural physiotherapy intervention for acute non-specific neck pain: A mixed-methods protocol
Wootton	2019	*BMJ Open*	Telehealth and texting intervention to improve HIV care engagement, mental health and substance use outcomes in youth living with HIV: A pilot feasibility and acceptability study protocol

For peer review only

Yeung	2019	*BMJ Open*	Randomised controlled trial to investigate the effectiveness of thoracic epidural and paravertebral blockade in reducing chronic post-thoracotomy pain (TOPIC): A pilot study to assess feasibility of a large multicentre trial
Abokhrais	2018	*PAFS*	A pilot randomised double blind controlled trial of the efficacy of purified fatty acids for the treatment of women with endometriosis-associated pain (PurFECT): Study protocol
Artom	2019	*PAFS*	Cognitive-behavioural therapy for the management of inflammatory bowel disease-fatigue: A feasibility randomised controlled trial
Aunger	2019	*PAFS*	A novel behavioural INTErvention to REduce Sitting Time in older adults undergoing orthopaedic surgery (INTEREST): Protocol for a randomised controlled feasibility study
Bérubé	2019	*PAFS*	Feasibility of a tapering opioids prescription program for trauma patients at high risk of chronic consumption (TOPPtrauma): Protocol for a pilot randomized controlled trial
Bick	2019	*PAFS*	Protocol for a two-arm feasibility RCT to support postnatal maternal weight management and positive lifestyle behaviour in women from an ethnically diverse inner city population: The SWAN feasibility trial
Bjornstad	2019	*PAFS*	Healthy Parent Carers peer-led group-based health promotion intervention for parent carers of disabled children: Protocol for a feasibility study using a parallel group randomised controlled trial design
Blanton	2019	*PAFS*	A web-based carepartner-integrated rehabilitation program for persons with stroke: Study protocol for a pilot randomized controlled trial
Bostrøm	2019	*PAFS*	Clinical comparative effectiveness of acupuncture versus manual therapy treatment of lateral epicondylitis: Feasibility randomized clinical trial
Bourne	2019	*PAFS*	Electrically assisted cycling for individuals with type 2 diabetes mellitus: Protocol for a pilot randomized controlled trial
Bowyer-Crane	2019	*PAFS*	A randomised controlled feasibility trial and qualitative evaluation of an early years language development intervention: Study protocol of the 'outcomes of Talking Together evaluation and results' (oTTer) project
Bryant	2018	*PAFS*	Cluster randomised controlled feasibility study of HENRY: A community-based intervention aimed at reducing obesity rates in preschool children
Bui	2019	*PAFS*	App-based supplemental exercise during inpatient orthopaedic rehabilitation increases activity levels: A pilot randomised control trial
Carswell	2019	*PAFS*	Implementing an arts-based intervention for patients with end-stage kidney disease whilst receiving haemodialysis: A feasibility study protocol 11 Medical and Health Sciences 1117 Public Health and Health Services 11 Medical and Health Sciences 1103 Clini
Clark	2019	*PAFS*	Saline versus albumin fluid for extracorporeal removal with slow low efficiency dialysis (SAFER-SLED): Study protocol for a pilot trial

For peer review only

Coe	2018	*PAFS*	A protocol for a randomised double-blind placebo-controlled feasibility study to determine whether the daily consumption of flavonoid-rich pure cocoa has the potential to reduce fatigue in people with relapsing and remitting multiple sclerosis (RRMS)
Courtier	2018	*PAFS*	ACTIVE - A randomised feasibility trial study protocol of a behavioural intervention to reduce fatigue in women undergoing radiotherapy for early breast cancer: Study protocol
Cro	2018	*PAFS*	Measuring skin necrosis in a randomised controlled feasibility trial of heat preconditioning on wound healing after reconstructive breast surgery: Study protocol and statistical analysis plan for the PREHEAT trial
De Oliveira Braga	2019	*PAFS*	EMPOWER-PD - A physical therapy intervention to empower the individuals with Parkinson's disease: A study protocol for a feasibility randomized controlled trial
Deary	2018	*PAFS*	A psychosocial intervention for the management of functional dysphonia: Complex intervention development and pilot randomised trial
Ditai	2019	*PAFS*	BabyGel pilot: A pilot cluster randomised trial of the provision of alcohol handgel to postpartum mothers to prevent neonatal and young infant infection-related morbidity in the community
Downey	2018	*PAFS*	Trial of Remote Continuous versus Intermittent NEWS monitoring after major surgery (TRaCINg): Protocol for a feasibility randomised controlled trial
Drew	2019	*PAFS*	A protocol for a randomised controlled, double-blind feasibility trial investigating fluoxetine treatment in improving memory and learning impairments in patients with mesial temporal lobe epilepsy: Fluoxetine, Learning and Memory in Epilepsy (FLAME trial
Duncan	2018	*PAFS*	Physical therapy and deep brain stimulation in Parkinson's Disease: Protocol for a pilot randomized controlled trial
Dunn	2019	*PAFS*	Evaluating Augmented Depression Therapy (ADepT): Study protocol for a pilot randomised controlled trial
Fuller	2018	*PAFS*	The ACUTE (Ambulance CPAP: Use, Treatment effect and economics) feasibility study: A pilot randomised controlled trial of prehospital CPAP for acute respiratory failure
Golla	2018	*PAFS*	Home-based balance training using Wii Fit™: A pilot randomised controlled trial with mobile older stroke survivors
Hayes	2019	*PAFS*	We Can Quit2 (WCQ2): A community-based intervention on smoking cessation for women living in disadvantaged areas of Ireland - Study protocol for a pilot cluster randomised controlled trial
Hilari	2019	*PAFS*	Adjustment with aphasia after stroke: Study protocol for a pilot feasibility randomised controlled trial for SUpporting wellbeing through PEeR Befriending (SUPERB)
Horne	2019	*PAFS*	Regaining Confidence after Stroke (RCAS): A feasibility randomised controlled trial (RCT)
Jones	2019	*PAFS*	Rapid Analgesia for Prehospital hip Disruption (RAPID): Findings from a randomised feasibility study

For peer review only

Kebbe	2019	*PAFS*	Feasibility, user experiences, and preliminary effect of Conversation Cards for Adolescents© on collaborative goal-setting and behavior change: Protocol for a pilot randomized controlled trial
Kohrt	2018	*PAFS*	Reducing stigma among healthcare providers to improve mental health services (RESHAPE): Protocol for a pilot cluster randomized controlled trial of a stigma reduction intervention for training primary healthcare workers in Nepal
Lodder	2019	*PAFS*	Stigma of living as an autism carer: A brief psycho-social support intervention (SOLACE). Study protocol for a randomised controlled feasibility study
Logan	2018	*PAFS*	Standing Practice In Rehabilitation Early after Stroke (SPIRES): A functional standing frame programme (prolonged standing and repeated sit to stand) to improve function and quality of life and reduce neuromuscular impairment in people with severe sub-acute stroke-a protocol for a feasibility randomised controlled trial
Loughnan	2019	*PAFS*	A single-centre, randomised controlled feasibility pilot trial comparing performance of direct laryngoscopy versus videolaryngoscopy for endotracheal intubation in surgical patients
Malden	2019	*PAFS*	A feasibility cluster randomised controlled trial of a preschool obesity prevention intervention: ToyBox-Scotland
McGovern	2018	*PAFS*	Promoting Alcohol Reduction in Non- Treatment Seeking parents (PAReNTS): A protocol for a pilot feasibility cluster randomised controlled trial of alcohol screening and brief interventions to reduce parental alcohol use disorders in vulnerable families
McIntosh	2018	*PAFS*	On the Road to Recovery psychological therapy versus treatment as usual for forensic mental health patients: Study protocol for a randomized controlled feasibility trial
Mehta	2019	*PAFS*	A randomised controlled feasibility trial to evaluate local heat preconditioning on wound healing after reconstructive breast surgery: The preHEAT trial
Meiksin	2019	*PAFS*	Protocol for pilot cluster RCT of project respect: A school-based intervention to prevent dating and relationship violence and address health inequalities among young people
Milbury	2018	*PAFS*	A research protocol for a pilot randomized controlled trial designed to examine the feasibility of a couple-based mind-body intervention for patients with metastatic lung cancer and their partners
Milbury	2019	*PAFS*	A research protocol for a pilot, randomized controlled trial designed to examine the feasibility of a dyadic versus individual yoga program for family caregivers of glioma patients undergoing radiotherapy
Moore	2018	*PAFS*	Prehospital recognition and antibiotics for 999 patients with sepsis: Protocol for a feasibility study
Morgan	2019	*PAFS*	A pilot randomised controlled trial of physical activity facilitation for older adults: Feasibility study findings
Morton	2018	*PAFS*	Chlorhexidine vaginal preparation versus standard treatment at caesarean section to reduce endometritis and prevent sepsis - A feasibility study protocol (the PREPS trial)

For peer review only

Murphy	2018	*PAFS*	Supporting general practitioner-based care for poorly controlled type 2 diabetes mellitus (the DECIDE study): Feasibility study and protocol for a pilot cluster randomised controlled trial
Mutedzi	2019	*PAFS*	Improving bereavement outcomes in Zimbabwe: Protocol for a feasibility cluster trial of the 9-cell bereavement tool
Myers	2019	*PAFS*	Accelerometer-based assessment of physical activity within the Fun for Wellness online behavioral intervention: Protocol for a feasibility study
Negm	2018	*PAFS*	Getting fit for hip and knee replacement: A protocol for the Fit-Joints pilot randomized controlled trial of a multi-modal intervention in frail patients with osteoarthritis
Newlands	2019	*PAFS*	Pilot randomised controlled trial of Weight Watchers® referral with or without dietitianled group support for weight loss in women treated for breast cancer: The BRIGHT (BReast cancer weIGHT loss) trial
O'Regan	2019	*PAFS*	An evaluation of an intervention designed to help inactive adults become more active with a peer mentoring component: A protocol for a cluster randomised feasibility trial of the Move for Life programme
Paul	2019	*PAFS*	Vital sign monitoring with continuous pulse oximetry and wireless clinical notification after surgery (the VIGILANCE pilot study)- A randomized controlled pilot trial
Payne	2018	*PAFS*	Study protocol for a randomised pilot study of a computer-based, non-pharmacological cognitive intervention for motor slowing and motor fatigue in Parkinson's disease
Perman-Howe	2018	*PAFS*	The effect of alcohol strength on alcohol consumption: A randomised controlled cross-over pilot trial
Philip	2019	*PAFS*	A randomised phase II trial to examine feasibility of standardised, early palliative (STEP) care for patients with advanced cancer and their families [ACTRN12617000534381]: A research protocol
Pile	2018	*PAFS*	A brief early intervention for adolescent depression that targets emotional mental images and memories: Protocol for a feasibility randomised controlled trial (IMAGINE trial)
Ponsford	2018	*PAFS*	Study protocol for the optimisation, feasibility testing and pilot cluster randomised trial of Positive Choices: A school-based social marketing intervention to promote sexual health, prevent unintended teenage pregnancies and address health inequalities
Purcell	2018	*PAFS*	Eutectic mixture of local anaesthetics (EMLA®) as a primary dressing on painful chronic leg ulcers: A pilot randomised controlled trial
Qurashi	2019	*PAFS*	Glycopyrrolate in comparison to hyoscine hydrobromide and placebo in the treatment of hypersalivation induced by clozapine (GOTHIC1): A feasibility study
Rowe	2019	*PAFS*	A classroom-based intervention targeting working memory, attention and language skills in 4-5 year olds (RECALL): Study protocol for a cluster randomised feasibility trial

For peer review only

Sanfilippo	2019	*PAFS*	A study protocol for testing the feasibility of a randomised stepped wedge cluster design to investigate a Community Health Intervention through Musical Engagement (CHIME) for perinatal mental health in the Gambia
Sangraula	2018	*PAFS*	Protocol for a feasibility study of group-based focused psychosocial support to improve the psychosocial well-being and functioning of adults affected by humanitarian crises in Nepal: Group Problem Management plus (PM+)
Schlaeger	2018	*PAFS*	Double-blind acupuncture needles: A multi-needle, multi-session randomized feasibility study
Schmitz	2019	*PAFS*	Impact of endurance exercise and probiotic supplementation on the intestinal microbiota: A cross-over pilot study
Shvedko	2018	*PAFS*	Physical Activity Intervention for Loneliness (PAIL) in community-dwelling older adults: Protocol for a feasibility study
Slobogean	2019	*PAFS*	Fixation using alternative implants for the treatment of hip fractures (FAITH-2): Design and rationale for a pilot multi-centre 2 × 2 factorial randomized controlled trial in young femoral neck fracture patients
Snowden	2018	*PAFS*	Preoperative Behavioural Intervention versus standard care to Reduce Drinking before elective orthopaedic Surgery (PRE-OP BIRDS): Protocol for a multicentre pilot randomised controlled trial
Sosnowski	2018	*PAFS*	A feasibility study of a randomised controlled trial to examine the impact of the ABCDE bundle on quality of life in ICU survivors
Tan	2019	*PAFS*	The efficacy of foot orthoses in individuals with patellofemoral osteoarthritis: A randomised feasibility trial
Timko	2018	*PAFS*	Cognitive remediation therapy (CRT) as a pretreatment intervention for adolescents with anorexia nervosa during medical hospitalization: A pilot randomized controlled trial protocol
Totty	2019	*PAFS*	Assessing the effectiveness of dialkylcarbamoylchloride (DACC)-coated post-operative dressings versus standard care in the prevention of surgical site infection in clean or clean-contaminated, vascular surgery (the DRESSINg trial): Study protocol for a pilot feasibility randomised controlled trial
Volkmer	2018	*PAFS*	The 'Better Conversations with Primary Progressive Aphasia (BCPPA)' program for people with PPA (Primary Progressive Aphasia): Protocol for a randomised controlled pilot study
Vranceanu	2019	*PAFS*	Results of a feasibility randomized controlled trial (RCT) of the Toolkit for Optimal Recovery (TOR): A live video program to prevent chronic pain in at-risk adults with orthopedic injuries
Whitehead	2019	*PAFS*	HATRIC: A study of Pelargonium sidoides root extract EPs®7630 (Kaloba®) for the treatment of acute cough due to lower respiratory tract infection in adults-study protocol for a double blind, placebocontrolled randomised feasibility trial
Wiggins	2018	*PAFS*	Testing the effectiveness of REACH Pregnancy Circles group antenatal care: Protocol for a randomised controlled pilot trial

For peer review only

Wong	2018	*PAFS*	Thiamine versus placebo in older heart failure patients: Study protocol for a randomized controlled crossover feasibility trial (THIAMINE-HF)
Wurz	2019	*PAFS*	Exploring the feasibility and acceptability of a mixed-methods pilot randomized controlled trial testing a 12-week physical activity intervention with adolescent and young adult cancer survivors
Hilton	2018	*PLoS ONE*	Randomised feasibility trial to compare three standard of care chemotherapy regimens for early stage triple-negative breast cancer (REaCT-TNBC trial)
Karlsson	2019	*PLoS ONE*	Feasibility of preoperative supervised home-based exercise in older adults undergoing colorectal cancer surgery – A randomized controlled design
Wiangkham	2019	*PLoS ONE*	A cluster randomised, double-blind pilot and feasibility trial of an active behavioural physiotherapy intervention for acute whiplash-associated disorder (WAD)II
Ahnfeldt	2019	*Trials*	FortiColos - A multicentre study using bovine colostrum as a fortifier to human milk in very preterm infants: Study protocol for a randomised controlled pilot trial
Barrett	2018	*Trials*	Feasibility of a physical activity programme embedded into the daily lives of older adults living in nursing homes: Protocol for a randomised controlled pilot feasibility study
Brennan	2018	*Trials*	Prevention of striae gravidarum: Study protocol for a pilot randomised controlled trial
Browne	2019	*Trials*	Probiotics in pregnancy: Protocol of a double-blind randomized controlled pilot trial for pregnant women with depression and anxiety (PIP pilot trial)
Burroughs	2018	*Trials*	A feasibility study for NOn-Traditional providers to support the management of Elderly People with Anxiety and Depression: The NOTEPAD study Protocol
Cao	2018	*Trials*	Aerobic exercise-based cardiac rehabilitation in Chinese patients with coronary heart disease: Study protocol for a pilot randomized controlled trial
Chhetri	2019	*Trials*	Repetitive vascular occlusion stimulus (RVOS) versus standard care to prevent muscle wasting in critically ill patients (ROSProx):a study protocol for a pilot randomised controlled trial
Crawford	2018	*Trials*	Psychological Support for Personality (PSP) versus treatment as usual: Study protocol for a feasibility randomized controlled trial of a low intensity intervention for people with personality disorder
Deb	2018	*Trials*	Aggression Following Traumatic brain injury: Effectiveness of Risperidone (AFTER): Study protocol for a feasibility randomised controlled trial
Forster	2018	*Trials*	An intervention to support stroke survivors and their carers in the longer term (LoTS2Care): Study protocol for a cluster randomised controlled feasibility trial

For peer review only

Froghi	2018	*Trials*	Cardiac output Optimisation following Liver Transplant (COLT) trial: Study protocol for a feasibility randomised controlled trial
Greenwood	2018	*Trials*	The U&I study: Study protocol for a feasibility randomised controlled trial of a pre-cognitive behavioural therapy digital 'informed choice' intervention to improve attitudes towards uptake and implementation of CBT for psychosis
He	2018	*Trials*	Xue-Fu-Zhu-Yu capsule in the treatment of qi stagnation and blood stasis syndrome: a study protocol for a randomised controlled pilot and feasibility trial
Hutchings	2018	*Trials*	CONTRACT Study - CONservative TReatment of Appendicitis in Children (feasibility): Study protocol for a randomised controlled Trial
Lee	2018	*Trials*	Effect and safety of acupuncture for Hwa-byung, an anger syndrome: A study protocol of a randomized controlled pilot trial
Linnemayr	2018	*Trials*	Behavioral economics-based incentives supported by mobile technology on HIV knowledge and testing frequency among Latino/a men who have sex with men and transgender women: Protocol for a randomized pilot study to test intervention feasibility and acceptability
Littlewood	2019	*Trials*	Protocol for a multi-centre pilot and feasibility randomised controlled trial with a nested qualitative study: Rehabilitation following rotator cuff repair (the RaCeR study)
Macken	2018	*Trials*	Palliative long-term abdominal drains versus repeated drainage in individuals with untreatable ascites due to advanced cirrhosis: Study protocol for a feasibility randomised controlled trial
Marsh	2018	*Trials*	A novel integrated dressing to secure peripheral intravenous catheters in an adult acute hospital: A pilot randomised controlled trial
Marsh	2018	*Trials*	Expert versus generalist inserters for peripheral intravenous catheter insertion: A pilot randomised controlled trial
Mayo-Wilson	2019	*Trials*	Microenterprise intervention to reduce sexual risk behaviors and increase employment and HIV preventive practices in economically-vulnerable African-American young adults (EMERGE): Protocol for a feasibility randomized clinical trial
Nymberg	2018	*Trials*	Pilot study on increased adherence to physical activity on prescription (PAP) through mindfulness: Study protocol
Pace	2019	*Trials*	Cognitively-Based Compassion Training versus cancer health education to improve health-related quality of life in survivors of solid tumor cancers and their informal caregivers: Study protocol for a randomized controlled pilot trial
Payne Riches	2019	*Trials*	The Salt Swap intervention to reduce salt intake in people with high blood pressure: Protocol for a feasibility randomised controlled trial

For peer review only

Poolman	2019	*Trials*	CARer-ADministration of as-needed subcutaneous medication for breakthrough symptoms in homebased dying patients (CARiAD): Study protocol for a UK-based open randomised pilot trial
Pressman	2019	*Trials*	Conducting a pilot randomized controlled trial of community-based mindfulness-based stress reduction versus usual care for moderate-to-severe migraine: Protocol for the Mindfulness and Migraine Study (M&M)
Pyle	2019	*Trials*	Study protocol for a randomised controlled trial of CBT vs antipsychotics vs both in 14-18-year-olds: Managing Adolescent first episode Psychosis: A feasibility study (MAPS)
Russell	2018	*Trials*	Feasibility of an online mindfulness-based program for patients with melanoma: Study protocol for a randomised controlled trial
Selfe	2019	*Trials*	Acceptability and feasibility of a 12-week yoga vs. educational film program for the management of restless legs syndrome (RLS): Study protocol for a randomized controlled trial
Taylor	2019	*Trials*	Protocol for a randomised controlled feasibility study examining the efficacy of brief cognitive therapy for the Treatment of Anxiety Disorders in Adolescents (TAD-A)
Van Oostveen	2018	*Trials*	Prevention of Infections in Cardiac Surgery study (PICS): Study protocol for a pragmatic cluster-randomized factorial crossover pilot trial
Watt	2019	*Trials*	A counseling intervention to address HIV stigma at entry into antenatal care in Tanzania (Maisha): Study protocol for a pilot randomized controlled trial
Wright	2018	*Trials*	The clinical and cost effectiveness of adapted dialectical behaviour therapy (DBT) for bipolar mood instability in primary care (ThrIVe-B programme): A feasibility study
Youssef	2019	*Trials*	Addition of a new three-dimensional adjustable cervical thoracic orthosis to a multi-modal program in the treatment of nonspecific neck pain: Study protocol for a randomised pilot trial
Zeng	2019	*Trials*	Si-ni-tang (a Chinese herbal formula) for improving immunofunction in sepsis: Study protocol for a pilot randomized controlled trial

BMJ: *British Medical Journal*; *PAFS*: *Pilot and Feasibility Studies; PLoS*: *Public Library of Science*

For peer review only

SUPPLEMENTARY FILE 3: INDICATOR OF FEASIBILITY CONTRIBUTING TO PROGRESSION CRITERIA

Domain	Indicator of feasibility contributing to progression criteria	Number of publications
Management	Data completion or missing data	38
	Barriers or challenges to intervention implementation	7
	Develop or test training materials	4
	Protocol components work together	2
	Data analysis	2
	Data collection methods are suitable/fit for purpose	1
	Data management	1
	Time to Ethics approvals at each site	1
	Time to readiness to initiate the clinical trial	1
Process	Recruitment rates	113
	Retention or attrition rate	106
	Non(compliance) or adherence rate (participants)	66
	Intervention acceptability or evaluation (patients)	33
	Withdrawal or completion rate (trial or intervention)	22
	Intervention acceptability or evaluation (non-patients)	17
	Consent or refusal rate	16
	Randomisation acceptability or rate	15
	Intervention fidelity	15
	Understanding or acceptability of data collection tools/assessments/methods	14
	Non(compliance) or adherence rates (non-participants)	12
	Participant identification or screening	10
	Eligibility rate	9
	Trial acceptability or evaluation (patients)	8
	Characteristics or properties of trial outcome measures	7
	Randomisation adequacy	5
	Crossover or contamination between arms	4
	Enrolment rate	5
	Uptake or engagement rate	3
	Definitive study sample size is achievable	3
	Trial acceptability or evaluation (non-patients)	2
	Success or failure rate	2
	Understanding or acceptability of study instructions	2
	Blinding procedures	1
	Eligibility criteria	1
	Describe control group	2
	Recruitment process	2
	Understanding or acceptability of study information	1
	Allocation concealment	1
	Intervention credibility	1
	Intervention suitability	1
	Completeness of biological sample collection	1
	Interest in using the intervention post-study	1
	Positive expected net gain of sampling from a definitive trial	1

For peer review only

Resources	Centre or investigator recruitment, willingness or capacity	21
	Determining process time	7
	Collection of outcomes relevant to future economic evaluation	4
	Equipment or resource reliability	2
	Venue, location or setting appropriate	1
	Determining capacity	1
	Intervention agreement between methods	1
Scientific	Safety, adverse events, unintended consequences or harms	25
	Estimate of treatment effect	11
	Estimate of variance of treatment effect	6
	Patient response	5
	Signal of efficacy	4
	Estimate intracluster correlation coefficient	1
	Context and mechanisms of action	1
	Intervention tolerability	1

For peer review only

PRISMA 2009 Checklist

Section/topic	#	Checklist item	Reported on page #
**TITLE**			
Title	1	Identify the report as a systematic review, meta-analysis, or both.	1
**ABSTRACT**			
Structured summary	2	Provide a structured summary including, as applicable: background; objectives; data sources; study eligibility criteria, participants, and interventions; study appraisal and synthesis methods; results; limitations; conclusions and implications of key findings; systematic review registration number.	2
**INTRODUCTION**			
Rationale	3	Describe the rationale for the review in the context of what is already known.	4
Objectives	4	Provide an explicit statement of questions being addressed with reference to participants, interventions, comparisons, outcomes, and study design (PICOS).	4
**METHODS**			
Protocol and registration	5	Indicate if a review protocol exists, if and where it can be accessed (e.g., Web address), and, if available, provide registration information including registration number.	4
Eligibility criteria	6	Specify study characteristics (e.g., PICOS, length of follow-up) and report characteristics (e.g., years considered, language, publication status) used as criteria for eligibility, giving rationale.	4
Information sources	7	Describe all information sources (e.g., databases with dates of coverage, contact with study authors to identify additional studies) in the search and date last searched.	5
Search	8	Present full electronic search strategy for at least one database, including any limits used, such that it could be repeated.	5
Study selection	9	State the process for selecting studies (i.e., screening, eligibility, included in systematic review, and, if applicable, included in the meta-analysis).	5
Data collection process	10	Describe method of data extraction from reports (e.g., piloted forms, independently, in duplicate) and any processes for obtaining and confirming data from investigators.	5
Data items	11	List and define all variables for which data were sought (e.g., PICOS, funding sources) and any assumptions and simplifications made.	5
Risk of bias in individual studies	12	Describe methods used for assessing risk of bias of individual studies (including specification of whether this was done at the study or outcome level), and how this information is to be used in any data synthesis.	N/A
Summary measures	13	State the principal summary measures (e.g., risk ratio, difference in means).	5
Synthesis of results	14	Describe the methods of handling data and combining results of studies, if done, including measures of consistency (e.g., I^2) for each meta-analysis.	5

For peer review only - http://bmjopen.bmj.com/site/about/guidelines.xhtml

PRISMA 2009 Checklist

Section/topic	#	Checklist item	Reported on page #
Risk of bias across studies	15	Specify any assessment of risk of bias that may affect the cumulative evidence (e.g., publication bias, selective reporting within studies).	N/A
Additional analyses	16	Describe methods of additional analyses (e.g., sensitivity or subgroup analyses, meta-regression), if done, indicating which were pre-specified.	N/A
**RESULTS**			
Study selection	17	Give numbers of studies screened, assessed for eligibility, and included in the review, with reasons for exclusions at each stage, ideally with a flow diagram.	6
Study characteristics	18	For each study, present characteristics for which data were extracted (e.g., study size, PICOS, follow-up period) and provide the citations.	6
Risk of bias within studies	19	Present data on risk of bias of each study and, if available, any outcome level assessment (see item 12).	N/A
Results of individual studies	20	For all outcomes considered (benefits or harms), present, for each study: (a) simple summary data for each intervention group (b) effect estimates and confidence intervals, ideally with a forest plot.	6-10
Synthesis of results	21	Present results of each meta-analysis done, including confidence intervals and measures of consistency.	N/A
Risk of bias across studies	22	Present results of any assessment of risk of bias across studies (see Item 15).	N/A
Additional analysis	23	Give results of additional analyses, if done (e.g., sensitivity or subgroup analyses, meta-regression [see Item 16]).	N/A
**DISCUSSION**			
Summary of evidence	24	Summarize the main findings including the strength of evidence for each main outcome; consider their relevance to key groups (e.g., healthcare providers, users, and policy makers).	10
Limitations	25	Discuss limitations at study and outcome level (e.g., risk of bias), and at review-level (e.g., incomplete retrieval of identified research, reporting bias).	10
Conclusions	26	Provide a general interpretation of the results in the context of other evidence, and implications for future research.	11
**FUNDING**			
Funding	27	Describe sources of funding for the systematic review and other support (e.g., supply of data); role of funders for the systematic review.	12

From: Moher D, Liberati A, Tetzlaff J, Altman DG, The PRISMA Group (2009). Preferred Reporting Items for Systematic Reviews and Meta-Analyses: The PRISMA Statement. PLoS Med 6(7): e1000097. doi:10.1371/journal.pmed1000097

For more information, visit: www.prisma-statement.org.

For peer review only - http://bmjopen.bmj.com/site/about/guidelines.xhtml
